# Automated Covalent Microcontact Bioprinting of Lubricant‐Infused Microarrays for Biological Assays

**DOI:** 10.1002/smll.202506732

**Published:** 2025-10-22

**Authors:** Lubna Najm, Amid Shakeri, Fereshteh Bayat, Shaghayegh Moghimikandelousi, Akansha Prasad, Liane Ladouceur, Samantha Dacalos, Sakina Hussain, Inaam Chattha, Hareet Sidhu, Zeinab Hosseinidoust, Tohid F. Didar

**Affiliations:** ^1^ Department of Mechanical Engineering McMaster University 1280 Main Street West Hamilton Ontario L8S 4L7 Canada; ^2^ Institute of Biomedical Engineering University of Toronto Toronto Ontario M5S 3G9 Canada; ^3^ School of Biomedical Engineering McMaster University 1280 Main Street West Hamilton Ontario L8S 4L7 Canada; ^4^ Department of Chemical Engineering McMaster University 1280 Main Street West Hamilton Ontario L8S 4L7 Canada; ^5^ Department of Biochemistry and Biomedical Sciences McMaster University 1280 Main Street West Hamilton Ontario L8S 4K1 Canada; ^6^ Institute for Infectious Disease Research (IIDR) McMaster University 1280 Main St W Hamilton Ontario L8S 4L8 Canada; ^7^ Farncombe Family Digestive Health Research Institute McMaster University 1280 Main St W Hamilton Ontario L8S 4L8 Canada

**Keywords:** automation, bacteriophage, failure mode and effects analysis, lubricant‐infused surfaces, microarrays, microcontact bioprinting

## Abstract

Microcontact bioprinting (µCP), which utilizes elastomeric stamps to transfer biorecognition agents (bioinks) onto substrates, offers advantages such as customizability, cost‐effectiveness, and versatility for bioassays. Despite its prevalent use in laboratory settings, µCP faces challenges in achieving the repeatability and reproducibility required for industrial applications. Here, a µCP method is introduced that accommodates the immobilization of various biorecognition agents, while preserving bioactivity for use in bioassays. A key innovation lies in combining µCP with fluorosilanization to enable lubricant‐infused surfaces that prevent non‐specific attachment, while concurrently enhancing biomolecule immobilization via covalent attachment through a modified bioink formulation. Furthermore, an automated µCP system is integrated, taking critical steps toward industrial scalability. Following optimization with fluorescent proteins, bacterial viruses (bacteriophages) are printed. The bioactivity preservation is confirmed using *Pseudomonas aeruginosa* bacteriophage microarrays. A bacterial metabolic activity bioassay is conducted, whereby bacteriophage lytic activity led to a visible color difference after 3 h. The introduced µCP is high‐throughput, scalable, and highly customizable, demonstrating strong potential for industrial implementation.

## Graphical abstract

The microcontact bioprinting protocol, through industrial risk assessment and optimization, has allowed to achieve scalability and automation of lubricant‐infused microarray fabrication. Additionally, bacteriophage microarrays created with this protocol showed preserved lytic activity and self‐propagation ability after bioprinting through an 2,3‐bis‐(2‐methoxy‐4‐nitro‐5‐sulfophenyl)‐2H‐tetrazolium1‐5‐carboxanilide, referred to as an XTT bacterial bioassay. Thus, making the protocol repeatable, reproducible, versatile, and highly effective for industrial microarray manufacturing.

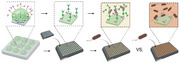

## 1 Introduction

Microarrays are commonly used in literature for their ease‐of‐integration within established biological and immunofluorescence assays (IFAs), the most common being sandwich enzyme‐linked immunosorbent assays (ELISAs).^[^ ^1^ ^]^ By combining microarrays and IFAs, target analytes and biomarkers are distinguishable, enhancing their applicability for diagnosing and monitoring disease in clinical and industrial settings.^[^ ^2^, ^3^, ^4^, ^5^, ^6^, ^7^, ^8^, ^9^, ^10^, ^11^ ^]^ A variety of fabrication methods, including both non‐contact and contact bioprinting techniques, are available for creating microarrays.^[^ ^10^, ^12^, ^13^ ^]^

Non‐contact bioprinting techniques, such as piezoelectric, continuous inkjet, and drop‐on‐demand printing, offer the advantage of precise, high‐speed deposition of bioinks onto substrates without direct contact.^[^ ^12^, ^14^, ^15^, ^16^, ^17^ ^]^ These methods are highly automated and suitable for large‐scale production; however, they often face limitations when printing highly viscous bioinks or large biorecognition agents due to issues like nozzle clogging and reduced resolution. Additionally, non‐contact systems can struggle to customize microarrays with intricate features, particularly when unique or complex patterns are required.^[^ ^14^, ^15^, ^18^ ^]^ In contrast, microcontact bioprinting (µCP), directly transfer bioinks from a patterned stamp onto the substrate, allowing for greater flexibility in feature design. µCP can handle a wider range of bioink viscosities, including large and complex biorecognition agents, and offer enhanced customizability in terms of pattern geometry.^[^ ^19^, ^20^ ^]^ However, contact bioprinting can face challenges with scalability and consistency in industrial settings due to the manual nature of the process.^[^ ^21^, ^22^ ^]^

µCP involves the fabrication of a stamp with micron features made from elastomers that are flexible, affordable, and easily accessible. The micron features are cast utilizing a silicon mold and can be predesigned with unique and fine detailed patterns and microarrays.^[^ ^8^, ^23^, ^24^, ^25^, ^26^, ^27^ ^]^ µCP involves the application of a bioink across the featured stamp surface, followed by physical force for direct biorecognition agent transfer onto substrates.^[^ ^8^, ^19^, ^21^, ^24^, ^28^ ^]^ The dimensions of the stamps allow µCP to cover a wide surface area upon a single print, making it faster than non‐contact printing or other contact methods such as pin or stencil printing, which often require multiple passes to print large areas.^[^ ^21^, ^23^ ^]^

Traditionally, µCP has two main limitations. It typically relies on the physical attachment of biorecognition agents to the surface, which reduces the robustness of the resulting microarrays. The introduction of cross‐linking agents within the bioink to induce covalent attachment as well as additives to control fluid evaporation rates can be investigated in µCP for microarray development.^[^ ^21^, ^24^, ^28^, ^29^ ^]^ µCP also has challenges in scalability for industry translation and is predominantly used in lab scale settings. This is mainly due to the lack of characterization and assessment of factors affecting the repeatability and reproducibility of µCP microarrays, resulting in low signal‐to‐noise ratios (SNRs) and high percentage coefficients of variance (%CV).^[^ ^12^, ^19^, ^30^, ^31^, ^32^, ^33^, ^34^, ^35^, ^36^ ^]^ Furthermore, the polydimethylsiloxane (PDMS) stamps used in the process can leave behind residue, leading to non‐specific attachment and ultimately reducing the sensitivity in bioassays.

Literature points to fluorosilanization, facilitating lubricant‐infusion, as a cost and resource‐effective solution to prevent non‐specific attachment, and thus increasing repeatability and reproducibility. Slippery surfaces created by fluorosilanization and lubrication ensure that non‐targeted molecules do not adhere to channel and chip surfaces. Slip chip microfluidic technology has enhanced sensitivity, precise microliter droplet formation and controlled fluid flow, allowing for single and multiplexed detection for a wide range of target analytes, including proteins, viruses, and nucleic acids.^[^ ^37^, ^38^, ^39^, ^40^, ^41^, ^42^, ^43^ ^]^ In µCP, incorporating slip chip fabrication strategies and lubricant‐infusion techniques have shown significant enhancement in sensitivity, for detecting cytokines in blood, ensuring cell adhesion for cell‐based assays, and conducting various types of bioassays.^[^ ^8^, ^44^, ^45^, ^46^, ^47^ ^]^ Characterization and optimization of lubricant‐infused microarrays after µCP is key in translating bioprinted microfluidic and sensing technologies from benchtop to industrial facilities.

Here, we propose a fully characterized µCP protocol meeting industry viable levels of repeatability and reproducibility, for industrial manufacturing applications of microfluidic and biosensing technologies. We developed a bioink formulation that enables cost‐effective, customizable and scalable covalent immobilization of a wide variety of biorecognition agents during µCP, that vary in size, function and structure, such as proteins, monoclonal antibodies and bacteriophages. Finally, we conduct a bacterial bioassay using bacteriophage microarrays, showing the versatility and applicability of our optimized µCP process while preserving bacteriophage activity.

## 2 Results and Discussion

In the following section, we established a fully optimized protocol for an industrialized µCP manufacturing process that incorporates both covalent carbodiimide chemistry and lubricant‐infused surfaces (LIS), which has not been previously reported in combination. We are the first study to leverage industry approved risk assessment tools in the optimization of µCP, allowing us to automate and standardize this bioprinting method for industry translation. By exploring the role of surface interactions and covalent binding in biorecognition agent immobilization and optimizing the impact of external factors on the µCP process, we achieved SNRs exceeding 15, along with intra‐assay and inter‐assay %CVs below 10%. Additionally, we report, for the first time, a fully functional microarray of lytic bacteriophage bioink, whereby the lytic bacteriophages retain their infectivity in their tail fibers after being µCP in a high‐throughput manner.

### 2.1 Stamp Fabrication, Substrate Functionalization, and Bioprinting Protocol

In creating the stamp mold, micron scale, and square pillar features were etched into a silicon wafer through photolithography, a highly consistent, robust technique. Square pillars were chosen as the feature design due to their ease of fabrication and image analysis. The square pillars dimensions of 150 µm × 150 µm × 1 µm and spaced equally apart by 150 µm, were chosen for their mechanical integrity with low deformation after multiple castings, resulting in higher consistency between stamp batches. Additionally, the edges and corners of the square pillars were slightly rounded out, whereby the degree of rounding was designed to ensure consistent polymer pooling each time new stamps were made, while maintaining the square pattern, minimizing batch‐to‐batch variability (Figure S1, Supporting Information).

Uncured PDMS at the ratio of 10:1, base: curing agent, was cast and cured on the fabricated mold to create flexible stamps for µCP using a highly repeatable and accurate soft lithography approach. PDMS, upon solidification, becomes a flexible elastomer with intermediate liquid absorption characteristics, making it ideal for absorbing bioink and facilitating bioink transfer onto functionalized substrates.^[^ ^23^, ^44^, ^48^, ^49^, ^50^, ^51^ ^]^ Before PDMS casting occurred, the mold was fluorosilanized, ensuring smooth peeling of the solidified PDMS and preventing PDMS residue from sticking to the mold after each use. A 3D printed chamber for accurate pouring, as well as cleaning the mold each time with isopropyl alcohol to prevent damage, ensured each stamp batch was as consistent as possible. Each stamp has a dimension of 2 cm × 1.8 cm × 0.3 cm as a result (Figure S1, Supporting Information). The stamps were also imaged each time before printing under the microscope to ensure consistency between batches.

Poly (methyl methacrylate) (PMMA) slides, were used as substrates in µCP to ensure the cost‐effectiveness for industry scale usability.^[^ ^52^ ^]^ Medical grade high quality PMMA was chosen, and each lot was tested for consistent surface properties, such as hydrophobicity, using contact angle measurements, each time before any functionalization occurred, to ensure minimal lot‐to‐lot variability. The substrates were then functionalized with CO_2_ plasma treatment, depositing activated carboxyl groups and rendering the substrates hydrophilic to ensure maximal bioink transfer.^[^ ^8^, ^51^, ^53^, ^54^, ^55^, ^56^, ^57^, ^58^, ^59^, ^60^ ^]^ The initial contact angle (CA) of untreated PMMA was 66.03°, indicating a relatively hydrophobic surface. By performing carbon dioxide (CO_2_) plasma treatment, for 3 minutes (min) to 15 min, the CAs significant decreased to 55.7° to 38.9°, respectively, making the substrate sufficiently hydrophilic for µCP. Extending the plasma treatment to 30 min caused the CA to drop further to 19.9°. We realized that this prolonged treatment resulted in over‐etching, damaging the already formed carboxyl groups and ultimately reducing the effectiveness of covalent attachment during µCP.^[^ ^61^, ^62^ ^]^ Therefore, 15 min plasma treatment was chosen as the optimum time to provide a degree of hydrophilicity while ensuring activated carboxyl groups remain on the functionalized PMMA substrates (Figures S2 and S3, Supporting Information).

X‐ray photoelectron spectroscopy (XPS) was also conducted before and after CO_2_ plasma treatment (Figures S4–S9, Supporting Information), and peak area percentages (peak area %) were quantified for each functional group. From Table S1 (Supporting Information), the carbon‐to‐carbon or carbon‐to‐hydrogen (C─C/C─H) bonds, at binding energy 284.69 eV, decreased as the peak area % drop from 58.99% before plasma treatment to 50.23% after plasma treatment. Meanwhile, the peak area % increased for the O─C═O bond, at binding energy 288.52 eV, from 17.04% before plasma treatment to 24.34% after plasma treatment, indicative of carboxyl group deposition.

To induce covalent cross‐linking through µCP, our unique protocol uses 1‐Ethyl‐3‐(3‐dimethylaminopropyl) carbodiimide/N‐Hydroxysuccinimide (EDC/NHS) along with the biorecognition agent of interest in the bioink formulation. We chose EDC/NHS due to its ease of accessibility, simple implementation within bioink, cost‐effective outsourcing, effective carbodiimide chemistry, and ease‐of‐integration with LIS. In comparison, literature reported µCP protocols, as shown in Table S2 (Supporting Information), are highly costly, require multiple synthesis steps and incubations, and have complex adhesion chemistry, preventing industry translation.^[^ ^63^, ^64^, ^65^, ^66^, ^67^, ^68^, ^69^, ^70^, ^71^, ^72^, ^73^, ^74^ ^]^

**Figure 1:**
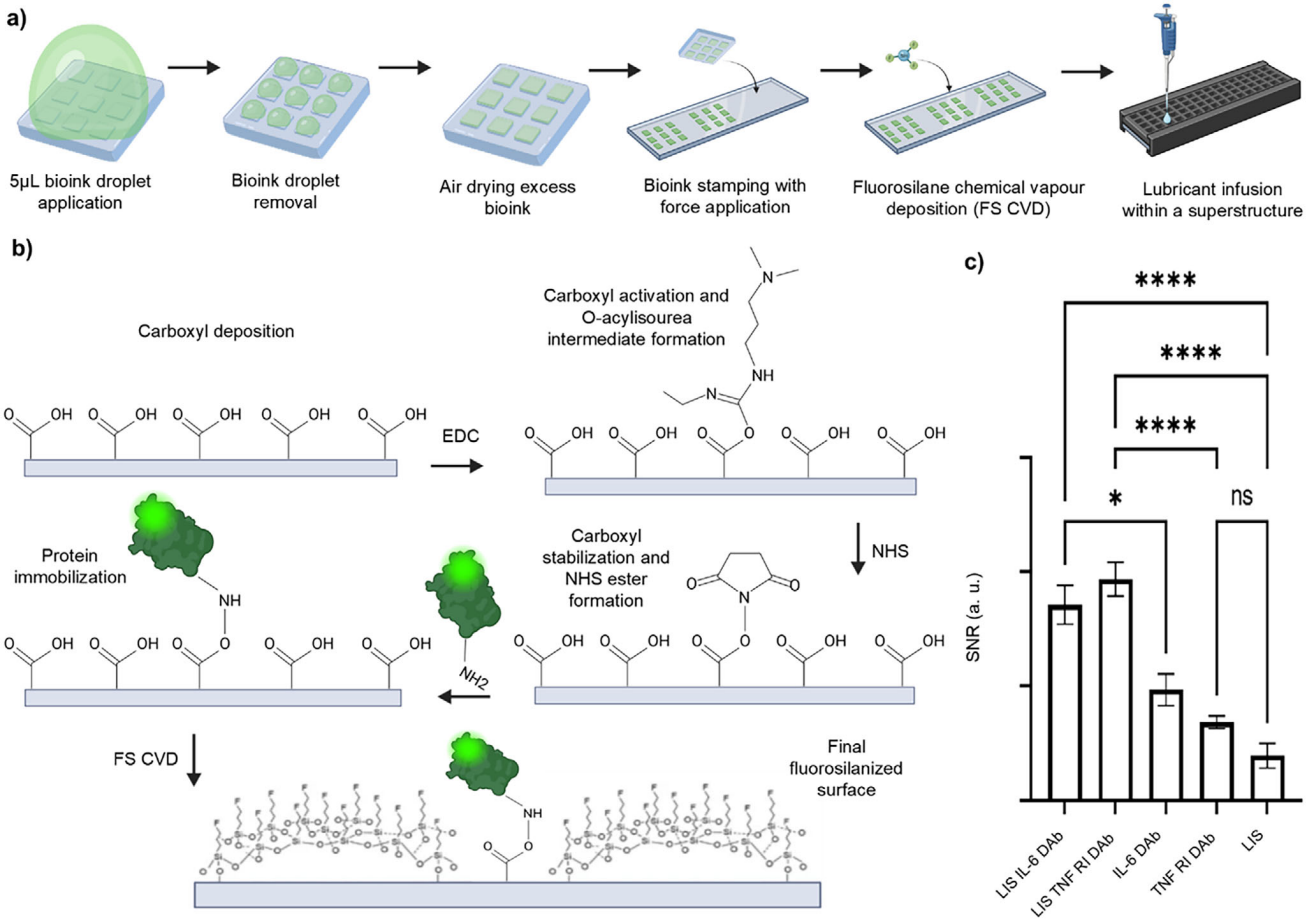
Overview of µCP protocol. a) Depicts the key steps in the µCP protocol, starting from bioink application, removal, air drying, stamping with force application, FS CVD, and lubricant‐infusion. Schematics created in Biorender.com. b) Depicts the surface chemistry of the EDC/NHS covalent cross‐linking for protein immobilization, whereby the O‐acylisourea intermediate is stabilized with NHS esters that facilitate the carbodiimide bond. Afterward, fluorosilanization via CVD leads to the formation of FS self‐assembled monolayer, ready for lubricant‐infusion. Schematics created in Biorender.com. c) SNRs after µCP IL‐6 and TNF RI DAb with and without LIS, compared to control of an LIS microarray without any biorecognition agents printed (*n* = 18). Error bars calculated using standard error of the mean. Statistical analysis was conducted using one‐way ANOVA followed by Tukey's post hoc test, with ns statistical values where *P* = 0.5, and 1‐star showing significance with *P* < 0.1, 2‐star with *P* < 0.01, 3‐star with *P* < 0.001 and 4‐star with *P* < 0.0001.

To illustrate the efficacy of µCP in each step, as shown in **Figure** **1**a, we utilized bovine serum albumin‐fluorescein isothiocyanate (BSA‐FITC) as the desired biorecognition agent in our bioink. Here, carbodiimide chemistry was important in the surface immobilization of the proteins and antibodies (Figure 1b) The bioink, containing EDC/NHS and BSA‐FITC, was first applied on the PDMS stamps in the form of 5 µL droplets. The droplets were incubated on the stamp's square pillar features, allowing bioink to get absorbed into the material of each feature. After incubation, removal of excess volume and subsequent air drying was conducted, allowing for evaporation of the bioink (Figure S10, Supporting Information). The elastomeric stamp was then placed onto the functionalized PMMA substrate. Force was applied onto the stamp for physical transfer of the bioink from the stamp features onto the substrate, facilitating biorecognition agent immobilization (Figure 1a). We measured the fluorescence signal of the patterns and background (noise) after printing. The results were indicated as SNRs for comparing different conditions. When comparing short and long incubation times after adding the bioink droplets onto the stamps, 2 min droplet incubation showed significantly higher SNR value compared to a longer incubation of 30 min. Thus, 2 min was sufficient for bioink absorption (Figure S11, Supporting Information).

The PDMS stamps also showed durability and reusability, making them effective for up to 10 consecutive prints before needing to be replaced, referred to as ‘aged stamps’ (Figure S12, Supporting Information). To increase the efficiency of µCP, we tested different stamp treatment methods to prevent transferring PDMS residue and optimize the surface energy of the stamp. After evaluating 3 min oxygen (O_2_) plasma treatment, fluorosilane (FS) treatment using chemical vapor deposition (CVD),^[^ ^8^, ^29^, ^53^, ^54^, ^75^ ^]^ and (3‐Aminopropyl) triethoxysilane (APTES) treatment,^[^ ^76^ ^]^ the untreated stamps proved to have better functionality, significantly higher SNRs and more uniform fluorescence patterns (Figures S13 and S14, Supporting Information).

In comparing treatment types, hydrophobic stamps, such as FS treated or untreated stamps (as PDMS is naturally hydrophobic^[^ ^77^ ^]^), out‐performed hydrophilic stamp treatments. Upon droplet application, in the case of hydrophilic stamps, the bioink spread and was further absorbed into the PDMS material, making it difficult for bioink transfer during force application without smearing or introducing residue (Figure S15, Supporting Information). It was observed that after printing, high quantities of bioink residue remained on the stamp surface after only one print.

Charge also influences bioink transfer, as shown by the significant difference in SNRs observed between charged and non‐charged stamps, specifically the untreated condition as PDMS is naturally a charge‐neutral material (Figures S13 and S14, Supporting Information).^[^ ^78^ ^]^ The introduction of charge results in electrostatic interactions between the treated PDMS stamps and the charged components of the biomolecules in the bioink upon droplet application.^[^ ^79^, ^80^, ^81^ ^]^ For instance, FS and O_2_ plasma treatment leads to charged stamp surfaces, which attract amino acid pockets of the opposite charge on proteins and antibodies, resulting in increased adsorption. In turn, bioink transfer becomes difficult due to strengthened adsorption, which does not occur when the surface is noncharged.^[^ ^79^, ^81^, ^82^, ^83^ ^]^

After µCP, the printed substrates were fluorosilanized using CVD followed by superstructure assembly and the introduction of perfuoroperhydrophenanthrene (PFPP) lubricant (Figure 1a).^[^ ^8^, ^53^, ^54^, ^59^ ^]^ The CAs after fluorosilanization increased significantly, showing that the printed PMMA was rendered hydrophobic. There was also significant difference between the CA of unprinted and printed fluorosilanized PMMA, indicating that washing the substrate allows for the removal of potential fluoro‐organic compound contamination on the printed microarrays (Figure S3, Supporting Information). Additionally, XPS data shows that fluorocarbon groups are only introduced to the PMMA substrates after fluorosilanization (Figures S4–S9 and Table S1, Supporting Information).

The LIS was leveraged as a blocking strategy for the microarray. The lubricant‐infused microarrays with dual‐immobilization showed enhanced SNRs and reduced background noise when biotinylated monoclonal antibodies were covalently µCP (Figure 1c). To assess the functionality of the lubricant‐infused microarray as a blocking agent for SNR enhancement, biotinylated monoclonal detector antibodies (DAb) for interleukin‐6 (IL‐6) and tumor necrosis factor receptor 1 (TNF R1) were printed separately, with one set of wells containing PFPP lubricant layer and one set of wells without lubricant. These were compared to a control of only PFPP, and streptavidin cyanine‐5 (Cy5) was used for fluorescence detection. As shown in Figure 1c, the SNRs of both lubricant‐infused antibody microarrays significantly increased compared to standard microarrays without lubricant, in the cases of IL‐6 DAb and TNF R1 DAb, respectively.

Failure Mode and Effects Analysis (FMEA) was conducted for each of the µCP steps, assessing the possibility of failure modes that could occur within our protocol.^[^ ^8^, ^84^, ^85^, ^86^, ^87^, ^88^ ^]^ The steps within our protocol deemed to have highest risk priority number (RPN) based on the product of severity (SEV), frequency of occurrence (OCC), and detectability of failure (DET), were as follows: 1) plasma treatment procedure (RPN = 70); 2) the formulation of the bioink (RPN = 140); 3) the droplet removal process (RPN = 280); 4) the conditions during airdrying (RPN = 392); and 5) force application (RPN = 128) (Table S3, Supporting Information). These five steps were then addressed and optimized in the bioprinting of BSA‐FITC, for enhancing repeatability and reproducibility of microarrays using µCP. Additionally, by creating a streamlined method of optimization, our FMEA allows for customizability, easily translating our protocol to the immobilization of antibodies and bacteriophages.

### 2.2 Optimization of Bioink Formulation

The first high‐risk step determined by FMEA was the optimization of the bioink formulation. Without covalent attachment, and only relying on physical biorecognition agent transfer, the immobilization was not strong enough to withstand washing stages necessary after lubrication (Figure S16, Supporting Information). Compared to conditions where no cross‐linking agent was present or only EDC was included in the bioink, we observed enhanced SNRs when both EDC and NHS were incorporated. As opposed to the O‐acylisourea intermediate formed by EDC, the NHS ester offers greater stability, which facilitates more efficient binding to the primary amines of biorecognition agents (Figure 1b). This is shown in SNRs between no EDC/NHS, only EDC and both EDC/NHS (Figure S17, Supporting Information).

In literature, molar ratios for EDC:NHS vary within the range of 1:1 – 1.5:1, and as such, we tested multiple stoichiometric ratios when optimizing EDC/NHS ratios.^[^ ^89^, ^90^, ^91^, ^92^ ^]^ The molar ratio of EDC to NHS that led to the most carbodiimide cross‐linking on the surface after washing, shown as the highest SNR values, was a 1.23:1 molar ratio of EDC to NHS (5mg EDC to 3mg NHS), among tested ratios of 1.05:1, 1.11:1, 1.23:1, 1.35:1, and 1.48:1 (Figure S18, Supporting Information). Furthermore, the concentration of BSA‐FITC, as the biorecognition agent of interest for printing, was optimized to 200µg/mL resulting in the highest SNR (Figure S19, Supporting Information).

**Figure 2:**
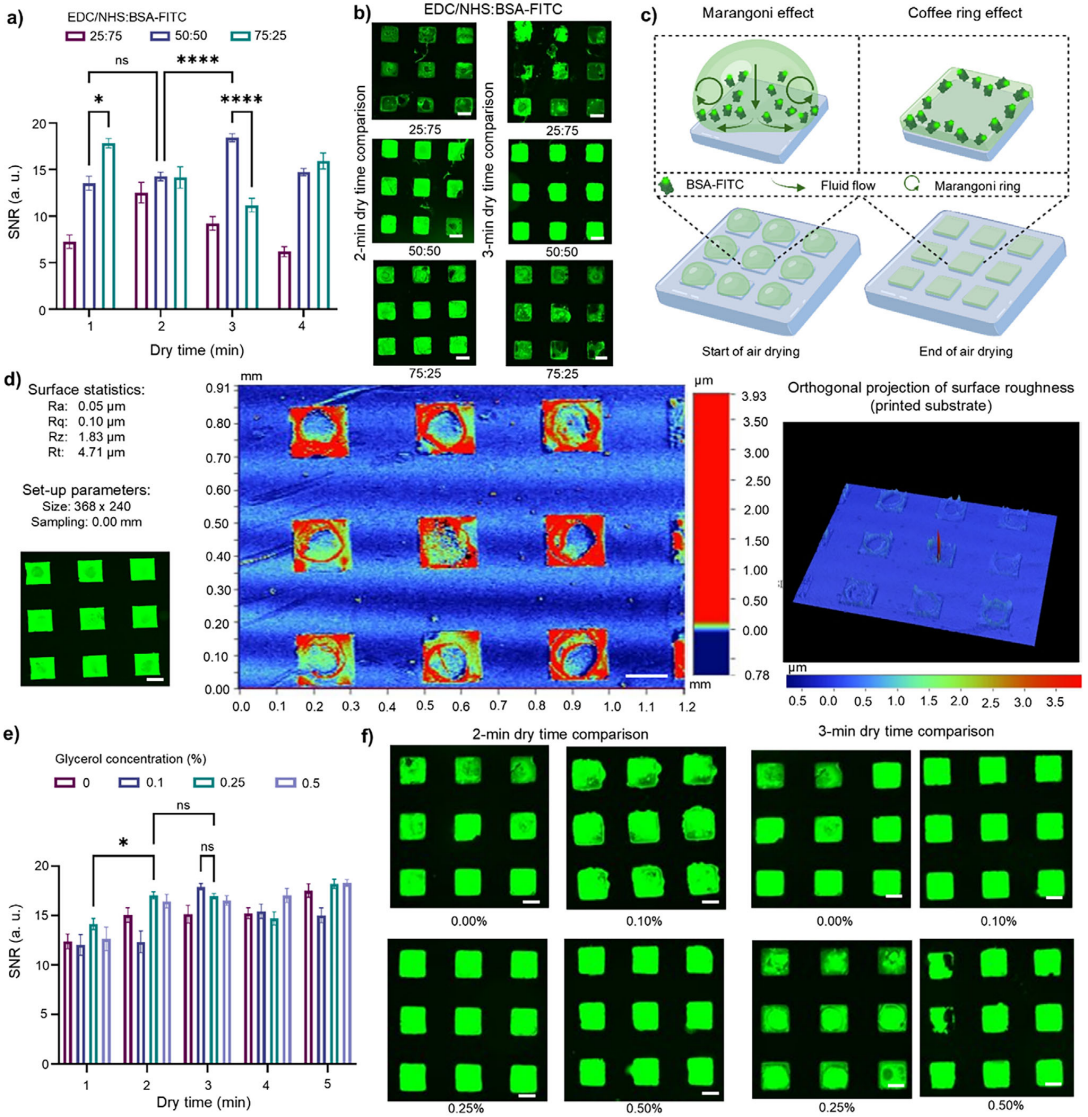
Optimization of bioink formulation. a) SNRs after µCP bioinks with EDC/NHS ratios of 25:75, 50:50, and 75:25, at dry times of 1, 2, 3, and 4 min, with b) fluorescence microscopy images associated with 2 and 3 min dry times (*n* = 18). Error bars calculated using standard error of the mean. Statistical analysis was conducted using two‐way ANOVA followed by Sidak's post hoc test, where ns statistical values, *P* = 0.5, and 1‐star showing significance with *P* < 0.1, 2‐star with *P* < 0.01, 3‐star with P <0.001 and 4‐star with *P* < 0.0001. c) Depicts the Marangoni Effect as stamps air dry, affecting the fluid flow and drying of bioink on individual stamp features, resulting in coffee ring effect. Schematics created in Biorender.com. d) Profilometry profile depicting coffee ring effect on printed PMMA slides, with fluorescent image and orthogonal projection of surface roughness, as comparison (*n* = 9). Scale bars were 100 µm. e) SNRs after µCP bioinks with glycerol concentrations of 0%, 0.1%, 0.25%, and 0.5% at dry times of 1, 2, 3, 4, and 5 min (*n* = 18). Error bars calculated using standard error of the mean. Statistical analysis was conducted using two‐way ANOVA followed by Sidak's post hoc test, where ns statistical values, *P* = 0.5, and 1‐star showing significance with *P* < 0.1, 2‐star with *P* < 0.01, 3‐star with P <0.001 and 4‐star with *P* < 0.0001. f) Sample fluorescence images of each glycerol concentration at 2‐min and 3‐min dry time. Scale bars were 100 µm.

The ratios of EDC and NHS to BSA‐FITC were assessed at 25:75, 50:50, and 75:25, as shown in **Figures** **2**a, 2b and S20 (Supporting Information). Among all three conditions, when EDC/NHS was less than BSA‐FITC, at a ratio of 25:75, the SNRs were significantly reduced across all dry times. This was expected, as less EDC/NHS to BSA‐FITC resulted in overall reduced covalent cross‐linking. The majority of the BSA‐FITC were subsequently printed solely with physical immobilization through force application, and not all the BSA‐FITC proteins were covalently cross‐linked, making the cross‐linking non‐uniform. When EDC/NHS levels were higher than BSA‐FITC levels in the bioink ratio 75:25, initial SNRs after 1 min airdrying were the highest among the three conditions, but then significantly reduced when airdrying time increased. This is because EDC/NHS is highly reactive, and as such, having higher levels of EDC/NHS caused either protein aggregation or salt formation, when left to airdry longer than 1 min. This is further shown in pockets with high fluorescent intensities within each square at the longer airdrying times, indicative of the formation of circular aggregates (Figure S20, Supporting Information). As highlighted in the FMEA, protein aggregation and salt formation from EDC/NHS reactivity are challenging to control. These issues lead to inconsistent fluorescence images, contributing to the high‐risk RPN for the bioink formulation step of µCP. The %CVs for all conditions remained above 10% for inter‐assay measurements, indicating non‐repeatable and non‐reproducible signals, which are unsuitable for industry applications (Table S4, Supporting Information).

We also tested µCP EDC/NHS separately, using a flat featureless stamp, before printing BSA‐FITC, with a featured stamp. This could potentially decrease the chance of biorecognition agent aggregation in the bioink during the incubation time on the stamp. However, separate printing proved to be not as effective for BSA‐FITC and required additional drying in the context of BSA‐FITC bioprinting but can be leveraged for the printing of other biorecognition agents by which may be initially inhibited by bioink mixing with EDC/NHS (Figure S21, Supporting Information).

When optimizing the bioink formulation, understanding the effects of fluid flow during the droplet incubation and airdrying processes of µCP pointed to the use of an additive or agent within the bioink to prevent failure modes. In sessile droplets, after the application of the bioink to the stamp, the biorecognition agents tend to flow toward the periphery of the droplet due to surface tension gradients along the liquid‐air interface, known as the Marangoni effect (Figure 2c).^[^ ^93^, ^94^ ^]^ This occurred whether higher or equal EDC/NHS to BSA‐FITC were used. To assess how the Marangoni effect affects bioprinting and BSA‐FITC immobilization after µCP, profilometry was conducted and surface topography was mapped on the printed substrate. As shown in the profilometry images (Figure 2d; Figure S22, Supporting Information) and roughness profiles (Figure S23, Supporting Information), the immobilized biorecognition agents accumulate along the edges, resulting in higher roughness values, with less biorecognition agents present in the center of the feature, known as a “coffee ring effect.” By increasing viscosity, the Marangoni effect can be suppressed and the coffee ring effects minimized, for more uniform immobilization of biorecognition agents.^[^ ^93^, ^94^, ^95^, ^96^, ^97^, ^98^, ^99^, ^100^ ^]^

Two additives of (3‐Glycidyloxypropyl)trimethoxysilane (GLYMO) and glycerol were assessed for the optimization of the bioink formulation. GLYMO is an epoxy‐based silane coupling reagent that can facilitate conjugation and chemical attachment of biorecognition agents to the surface through epoxy ring opening mechanism. However, in our experiment, epoxy showed reduced SNR of µCP microarrays, as it also prolonged the airdrying step (Figure S24, Supporting Information) The increased wetness from the epoxy‐based additive may stem from overly increasing the viscosity, resulting in a gel‐like droplet, making the droplet removal step inconsistent. The extreme wetness prevented rapid airdrying of the droplet, making the µCP unsuitable for industry fabrication, which requires high speed, seamless and efficient production. This suggested that an alternative additive was needed to increase the viscosity of the bioink and reduce Marangoni effect, without increasing wetness or inducing highly gel‐like bioink consistency.

Application of glycerol resulted in an increase in the SNR values. This could be attributed to the effect of glycerol in reducing Marangoni effects and air‐drying times compared to GLYMO. For biological applications, glycerol is preferred as, at low concentrations, it does not harm the integrity of biorecognition agents, such as proteins, antibodies and bacteriophages, but in fact, stabilizes them for longer time frames.^[^ ^101^, ^102^, ^103^, ^104^ ^]^ In the case of proteins and antibodies, aggregation and non‐specific attachment are minimized.^[^ ^101^, ^104^ ^]^ Additionally, unlike glucose, epoxy‐based additives, and poly(ethylene) glycol, other adjuvants reported in literature, glycerol has the highest viscosity and hygroscopic properties, as well as having no reported deliquescence point, making it effective up to 100% relative humidity.^[^ ^99^, ^105^, ^106^, ^107^, ^108^, ^109^, ^110^, ^111^ ^]^ Thus, glycerol showed more applicability in the µCP protocol for industrial fabrication as a more efficient and timelier bioink additive.^[^ ^95^, ^96^, ^100^ ^]^

We further investigated the optimum concentration of glycerol in the bioink (Figure 2d). While the SNR values showed no significant difference among various glycerol concentrations, the 0.25% glycerol bioink, compared to the 0.1% glycerol bioink, showed lower %CV overall within the same print (intra‐assay %CV), and between multiple prints (inter‐assay %CV), indicating better repeatability and reproducibility. Specifically, the intra‐assay %CVs were 8.81% and 13.58% for the 0.1% glycerol bioink, while for 0.25% glycerol bioinks, the intra‐assay %CVs were 8.05% and 5.65%, after 3 min dry time. Meanwhile, the inter‐assay %CVs were 11.19% and 6.85%, for the 0.1% and 0.25% glycerol bioinks, respectively (Table S5, Supporting Information). These differences in %CV are visible in the fluorescent images of all glycerol bioinks, as the 0.25% glycerol bioink had consistent square shapes and low background noise (Figure 2e,f; Figure S25, Supporting Information).

When assessing the airdrying time, 1 min airdrying showed significantly lower SNR results compared to 2 min, indicating that the excess bioink was not sufficiently evaporated with a 1 min airdrying time. The SNRs between 2 and 3 min were not significantly different, requiring further assessment using %CVs. The inter‐assay %CVs of 0.25% glycerol for the 2 and 3 min airdrying were 12.77% and 6.85%, respectively (Table S5, Supporting Information). The %CVs showed that 3 min airdrying had higher repeatability and reproducibility, despite 2 and 3 min conditions having similar SNR values. Through the assessment of glycerol, having uniform viscosity and fluid flow was essential for improving the immobilization of biorecognition agents throughout the printed substrates. The glycerol levels of 0.25% concentration, at 3 min airdrying, were shown to have maximal SNR and minimal %CV.

### 2.3 Optimization of Droplet Removal, External Factors, and Force Application

Three droplet removal strategies were assessed to determine the most effective method. These included wicking the droplet with a Kimwipe, applying uniform centrifugal force for even droplet spreading, and removing the droplet at its edge with a pipette. SNR and fluorescence images showed that pipette removal was the most accurate and precise droplet removal strategy, resulting in uniform and repeatable printed features (Figures S26 and S27, Supporting Information). The CV values for droplet removal strategies also showed that pipette droplet removal had the lowest inter‐assay %CV, of 8.22%, compared to Kimwipe (51.36%) and centrifuge (42.08%) (Table S6, Supporting Information).

Environmental factors, such as temperature and humidity control, play a key role in the airdrying protocol of our µCP process. Temperature and humidity have both been shown to affect the evaporation of drying sessile droplets.^[^ ^100^ ^]^ As such, confining the stamps within a controlled environment and assessing the effects of temperature and relative humidity was performed. Increasing temperature resulted in significant SNR reduction, pointing to the role of temperature on droplet evaporation, as shown in Figure S28 (Supporting Information).

**Figure 3:**
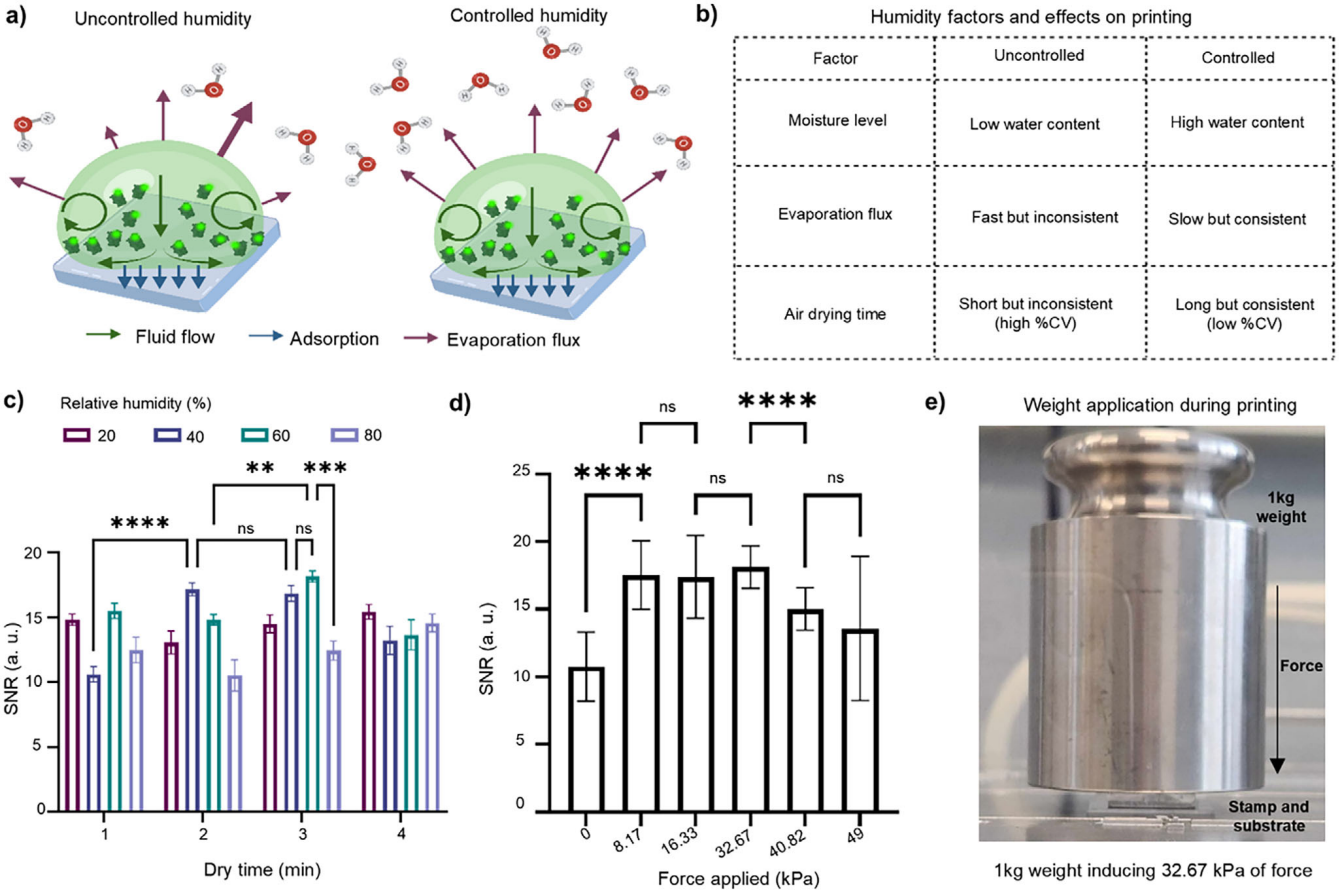
Optimization of external humidity and force application. a) Depicts the fluid flow, bioink adsorption and evaporation flux of bioink under uncontrolled and controlled humidity levels, during air drying on each stamp feature. Schematics created in Biorender.com. b) A comparison of moisture level, evaporation flux and air‐drying time between uncontrolled and controlled humidity environments. Schematics created in Biorender.com. c) SNRs after µCP stamps under controlled relative humidity levels of 20%, 40%, 60%, and 80%, at dry times of 1, 2, 3, and 4 min (*n* = 18). Error bars calculated using standard error of the mean. Statistical analysis was conducted using two‐way ANOVA followed by Sidak's post hoc test, where ns statistical values, *P* = 0.5, and 1‐star showing significance with *P* < 0.1, 2‐star with *P* < 0.01, 3‐star with *P* < 0.001 and 4‐star with *P* < 0.0001. d) SNRs after µCP stamps using applied forces of 0, 8.17, 16.33, 32.67, 40.82, and 49 kPa (*n* = 18). Error bars calculated using the mean of standard deviation. Statistical analysis was conducted using one‐way ANOVA followed by Tukey's post hoc test, with ns statistical values where *P* = 0.5, and 1‐star showing significance with *P* < 0.1, 2‐star with *P* < 0.01, 3‐star with *P* < 0.001 and 4‐star with *P* < 0.0001. e) Image depicting the weight application during printing of 32.67 kPa force.

By controlling and increasing the environmental humidity to slow the evaporation rate, the consistency of the flux significantly improved compared to conditions with uncontrolled humidity, as outlined in **Figure** **3**a,b. Various levels of controlled humidity were evaluated, with the highest SNR observed at 60% relative humidity for 3 min drying time. However, there was no significant difference in SNR between 40% and 60% relative humidity for the same drying time. This suggests that the minimum humidity required to achieve SNR above 15 was 40%. At extreme humidity levels, such as 20% or 80%, the SNRs did not surpass 15 (Figure 3c). This outcome was likely due to the insufficient moisture at low humidity to establish an effective evaporation flux, or excessive moisture at high humidity, which hindered evaporation and prevented proper drying.

The relative humidity (40% and 60%) were assessed for consistency at the 2 and 3 min dry times to determine ideal environmental humidity (Table S7, Supporting Information). The intra‐assay %CVs of two wells for the 2 min dry time at 40% relative humidity were 4.60% and 16.45%, with the inter‐assay %CV of 12.27%. In comparison, when increasing the dry time to 3 min, the intra‐assay %CVs were reported as 16.17% and 7.41%, while the inter‐assay %CV increased to 15.34%. The results showed that at 40% relative humidity, a 2 min drying time provided more consistency; however, with %CV values exceeding 10%, this condition was not suitable for achieving the industrial standards of repeatability and reproducibility. In comparison, 60% of the relative humidity at 3 min was able to achieve lower intra‐assay %CVs of 9.25% and 11.13%, with an inter‐assay %CV of 9.94%. This was also evident in the fluorescent images, where 60% of the relative humidity had the nicest shaped squares and least visible background noise (Figure S29, Supporting Information). The higher consistency, despite the longer dry time, makes 60% relative humidity at 3 min dry time the most repeatable and reproducible for industry applications. The longer drying time was due to the more balanced humidity environment, which created a uniform evaporation flux that slowed down compared to 40% relative humidity. Thus, 60% relative humidity and 3 min dry time were chosen as the optimized factors.

The last step for optimization of the µCP protocol was determining the maximal force application for the highest bioink physical transfer and BSA‐FITC immobilization (Figure 3d,e; Figure S30, Supporting Information). The assessment was done by application of 0 and 49 kPa pressure on the stamps. The SNR values demonstrate that without force application (0 kPa), the SNR value was minimum (Figure 3d). The SNR values significantly increased to values higher than 15 after the application of force. This determines that the force application ensures more effective physical bioink transfer from the stamp to the substrate. Inconsistencies of the %CVs also exist when no pressure is applied during the stamping process, at intra‐assay %CVs of 31.04% and 14.01%, and inter‐assay %CV of 23.75% (Table S8, Supporting Information). The fluorescence images for 0 kPa further depict the low bioink transfer (Figure S30, Supporting Information).

Among the pressures tested, the range of 8.17 to 32.67 kPa produced the highest SNRs, with no significant differences in SNR across this range (Figure 3d). Meanwhile, the %CV values were lowest when 32.67 kPa was applied, at intra‐assay %CVs of 6.76% and 2.47%, as well as inter‐assay %CVs of 4.99%. This shows that the value of 32.67 kPa was most repeatable and reproducible for industrial applications. Although 16.33 kPa resulted in intra‐assay %CVs of 5.69% and 10.05%, and inter‐assay %CVs of 8.91%, which are within acceptable industrial ranges for repeatability and reproducibility, 32.67 kPa demonstrated greater consistency overall, with lower %CVs (Table S8, Supporting Information). Additionally, among all the fluorescence images in Figure S30 (Supporting Information), 32.67 kPa produced the most uniform and complete squares.

Exceeding 32.67 kPa resulted in a significant reduction in SNR. At these high pressures, stamps also experienced more buckling, higher damage to the PDMS features and higher occurrences of double printing. Additionally, the inter‐assay values begin to increase and exceed the industrial threshold, reaching 10.48% and 27.65% for 40.82 and 49 kPa, respectively (Table S8, Supporting Information). While all squares were successfully printed, buckling of the stamp features caused visible holes in the fluorescent images, resulting in non‐uniform fluorescence and increased inconsistency (Figure S30, Supporting Information).

### 2.4 Uniform Droplet Dispensing and Force Application for Preliminary Automation

Through industry‐standard FMEA analysis, we were able to pinpoint key risk factors and work toward a fully optimized and enhanced µCP process. Regardless, manual µCP limits the translation of µCP to industry‐scale manufacturing. Automation of µCP would allow for rapid high‐throughput fabrication of lubricant‐infused microarrays, making it possible to integrate with current manufacturing machinery in industry.^[^ ^112^, ^113^, ^114^ ^]^ To standardize the µCP protocol and to achieve industry‐level scalability, we took the preliminary steps toward the automation of our optimized µCP protocol.

**Figure 4:**
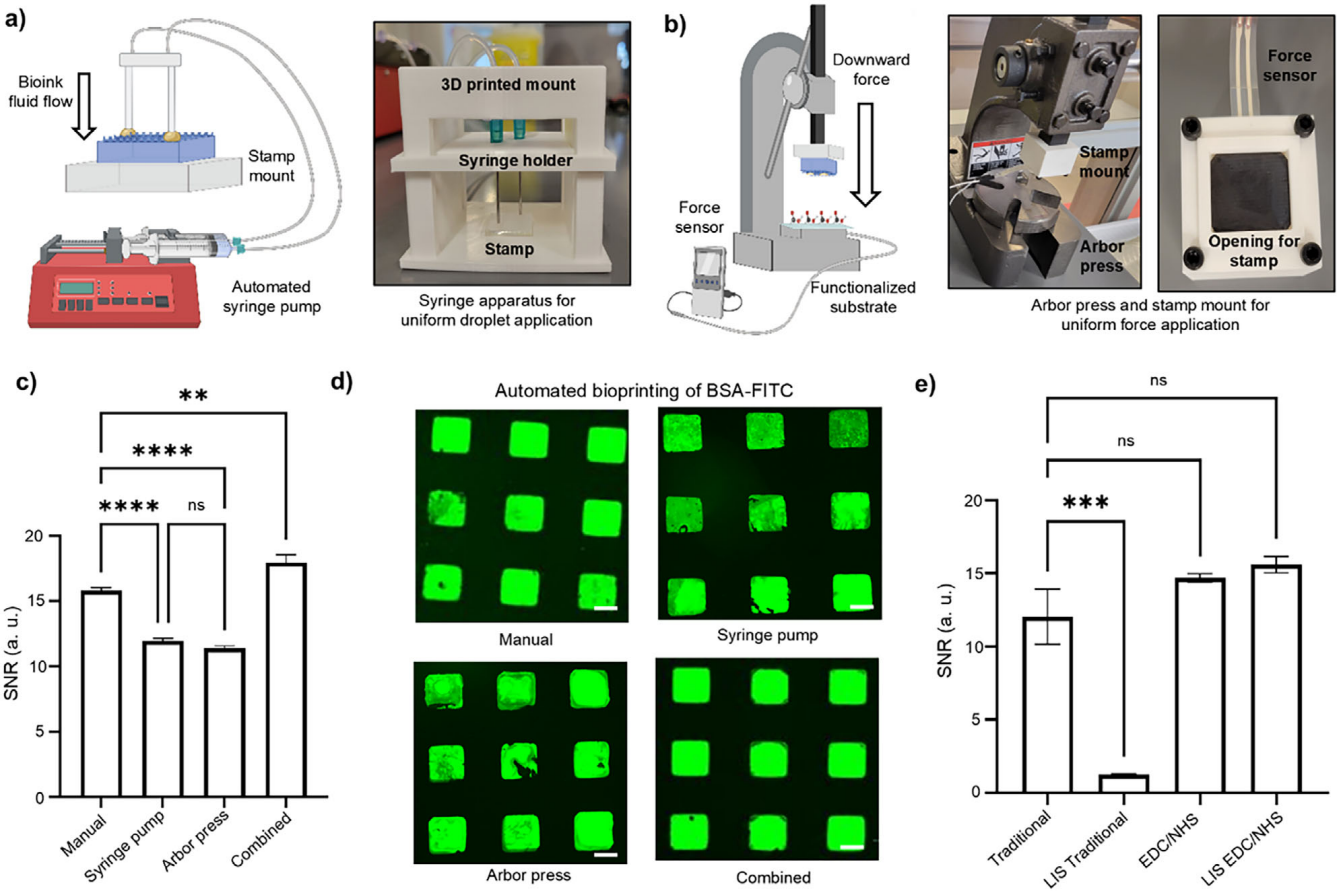
Depicts the standardization and preliminary automation steps of the optimized µCP protocol. a) Depicts the use of an automated syringe pump, stamp mount and syringe apparatus to control fluid flow upon droplet application and removal on the PDMS stamp. Schematics created in Biorender.com. b) Depicts the use of an arbor press, with the PDMS stamp mount loaded for physical bioink transfer to the functionalized substrate. A standardized force sensor was incorporated to ensure the application of constant downward force. Schematics created in Biorender.com. c) SNRs after µCP with optimized protocol conditions using manual printing, applying only the syringe pump, applying only the arbor press, and a combined syringe pump and arbor press set up (*n* = 54). Error bars calculated using standard error of the mean. Statistical analysis was conducted using one‐way ANOVA followed by Tukey's post hoc test, with ns statistical values where *P* = 0.5, and 1‐star showing significance with *P* < 0.1, 2‐star with *P* < 0.01, 3‐star with *P* < 0.001 and 4‐star with *P* < 0.0001. d) Fluorescent images of manual, syringe pump, arbor press, and combined set ups. Scale bars were 100 µm. e) SNRs of non‐lubricated and lubricated traditional microarrays, utilizing O_2_ plasma treatment and physical attachment only, compared to our optimized µCP microarrays, leveraging CO_2_ plasma treatment and dual‐immobilization attachment (*n* = 18). Error bars calculated using standard error of the mean. Statistical analysis was conducted using one‐way ANOVA followed by Tukey's post hoc test, with ns statistical values where *P* = 0.5, and 1‐star showing significance with *P* < 0.1, 2‐star with *P* < 0.01, 3‐star with *P* < 0.001 and 4‐star with *P* < 0.0001.

To automate the droplet addition and removal process, we developed a syringe pump‐operated mechanism. This system uses a 3D printed syringe apparatus connected to the pump via 1.5 mm diameter tubing for precise droplet dispensing. As shown in **Figure** **4**a, the syringe pump induced an automatic and consistent droplet dispensing and pick‐up rate to standardize the speed of droplet application and removal. Furthermore, weight application was also standardized through the incorporation of an arbor press to allow for consistent force application, as seen in Figure 4b. 3D printing of a syringe apparatus and stamping mount allowed to fine tune the processes for the optimized bioink volumes, viscosity and stamp dimensions (Figure 4a,b).

Three conditions were tested in comparison to manual printing: 1) syringe pump only, with manual stamping conducted; 2) Arbor press only, with manual droplet application and removal; and 3) a combined system of both syringe pump and arbor press. When compared to manual, as shown in Figure 4c, the combined system in which both syringe pump and arbor press were incorporated in the µCP protocol led to enhanced SNR with all optimized parameters. The %CV results shown in Table S9 (Supporting Information) indicate that the combined automated system had the lowest %CVs within industrial ranges, with intra‐assay %CVs of 7.08% and 3.29%, and inter‐assay %CV of 5.18%. Compared to manual printing, with intra‐assay %CVs of 9.79% and 5.66%, as well as inter‐assay %CV of 7.73%, the application of a fully automated system further reduced the inconsistency of µCP.

However, when only syringe pump for droplet addition and removal, or only the arbor press for force application, were used individually, the SNR was not enhanced as the failure modes identified in the FMEA remained possible. In fact, the SNRs were significantly reduced compared to manual printing, which could be due to the operator switching between manual and automated steps, prolonging the air‐drying process between droplet removal and stamping (Figure 4c). When air‐drying exceeded 3 min, as shown before, the SNR significantly decreased due to the over drying of the bioink, leading to inconsistencies in the final print. This was observed in the fluorescent images, where increased non‐uniformity is observed in the printed squares of the syringe pump‐only and arbor press‐only conditions (Figure 4d). The high inter‐assay values of 22.18% and 17.90%, for the syringe pump‐only and arbor press‐only, respectively, also depict the increase in inconsistency when relying on manual steps (Table S9, Supporting Information).

We then sought to compare our protocol to the traditional µCP protocols reported in literature that rely on physical attachment (Table S2, Supporting Information).^[^ ^63^, ^64^, ^65^, ^66^, ^67^, ^68^, ^69^, ^70^, ^71^, ^72^, ^73^, ^74^ ^]^ For the traditional µCP comparison, we performed 5 min O_2_ plasma treatment, and then bioprinted BSA‐FITC in the absence of any cross‐linker. This was compared to our µCP protocol, where we performed 15 min CO_2_ plasma treatment, and bioprinted a combination of BSA‐FITC and EDC/NHS.

The SNR with EDC/NHS was 14.68 for non‐lubricated microarrays and 15.59 for lubricant‐infused microarrays. In comparison, traditional µCP resulted in SNRs of 12.04 and 1.25 for nonlubricated and lubricant‐infused microarrays, respectively (Figure 4e). The microarrays with dual immobilization were overall more stable, as shown by the maintained SNRs between LIS and nonlubricated substrates. The dually immobilized microarrays were also able to maintain the microarray pattern in fluorescent microscopy images after applying a lubricant coating, without detachment, unlike the microarrays that were only immobilized physically, which quickly detached after lubricant‐infusion (Figure S31, Supporting Information). Similarly, after washing the printed substrates, the traditional microarrays had a significantly lower SNR of 3.23, compared to EDC/NHS µCP microarrays, at SNR value of 9.16 (Figure S32, Supporting Information). Again, this shows that covalent cross‐linking reduces detachment after three washing cycles, making dual immobilized microarrays more suitable for bioassays that have many washing and incubating steps compared to traditional counterparts.

When assessing consistency before lubricant‐infusion, the intra‐assay %CVs for the traditional microarrays were 22.15% and 26.65%, compared to 9.69% and 6.19% for microarrays bioprinted with EDC/NHS. When comparing inter‐assay %CVs, EDC/NHS µCP microarrays have 8.41%, which is more than 4X lower compared to their traditional counterparts, at 66.12% (Table S10, Supporting Information). The %CV values show that, in addition to enhancing the SNR strength and overall stability of the microarrays, EDC/NHS cross‐linking increases the repeatability and reproducibility of the stamping process.

### 2.5 Applications for Optimized Microarrays

Having established a customizable protocol leveraging FMEA to immobilize proteins and antibodies, we show the versatility of our µCP protocol for a more complex bioactive agent, specifically bacterial viruses, known as bacteriophages.

Bacteriophages are bactericidal viruses that are able to hijack and lyse bacteria when immobilized onto functionalized substrates.^[^ ^115^ ^]^ Bacteriophages can be highly specific in targeting bacterial cells, including multidrug resistant bacteria.^[^ ^115^, ^116^, ^117^ ^]^ Wild type or genetically modified bacteriophages immobilized onto surfaces can serve as a powerful tool for applications in biosensing, bacterial capture, peptide screening, antimicrobial surface fabrication and therapeutic coatings.^[^ ^118^, ^119^ ^]^ From an industrial perspective, bacteriophages are cheaper to use in bioassay manufacturing, are more stable under various conditions, nontoxic for clinical use and are highly abundant and available in nature, making them suitable for industrial scalability and optimization.^[^ ^116^, ^117^ ^]^ An intriguing feature of bacteriophages is their ability to self‐amplify by injecting their genome into target bacterial cells, hijacking the host's reproductive machinery to produce and assemble new virions. This process leads to bacterial cell lysis and the release of newly formed virions, known as the bacteriophage lytic cycle.^[^ ^115^ ^]^

**Figure 5:**
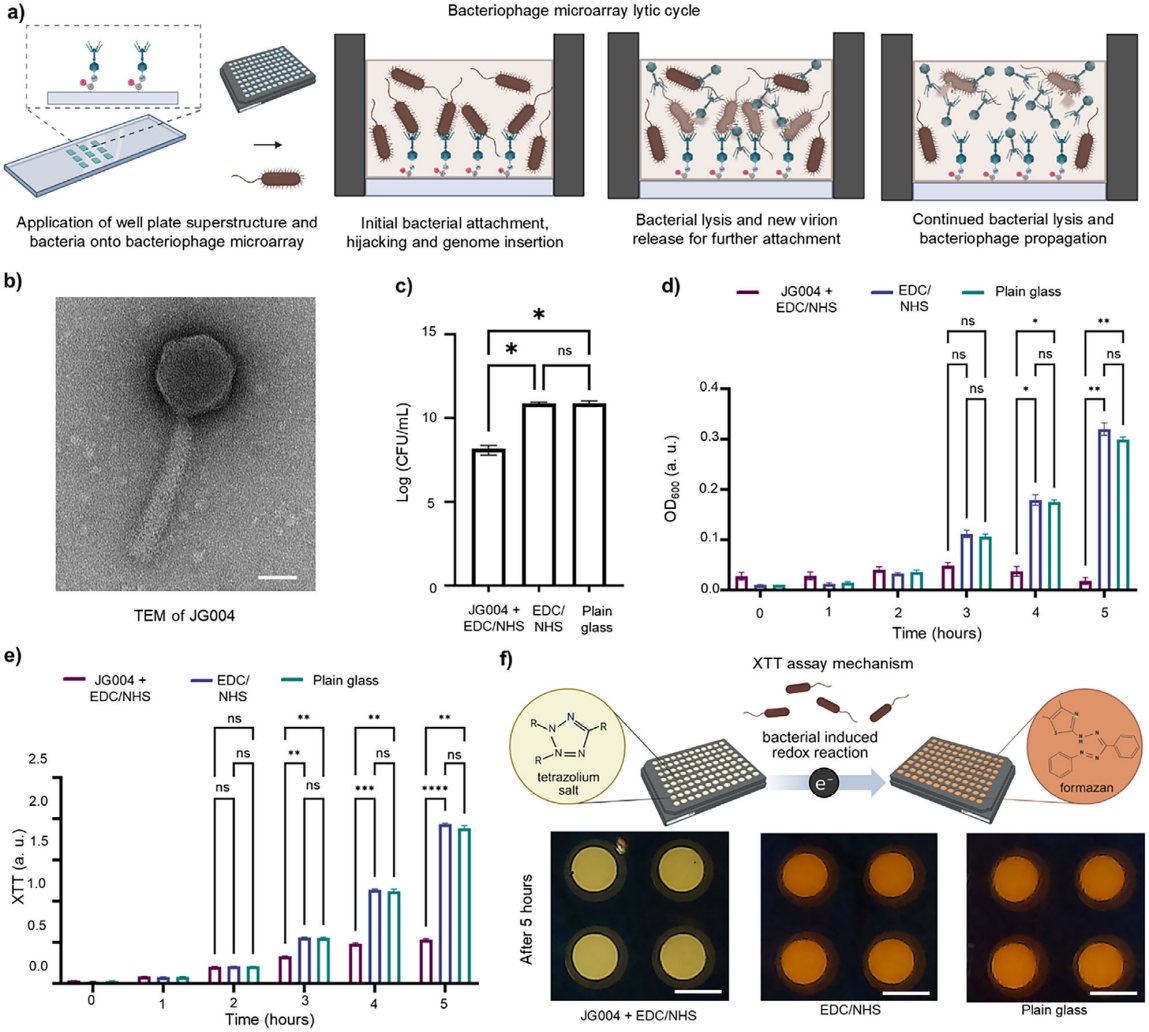
Depicts bacterial bioassay for bacteriophage microarrays. a) Depicts immobilized bacteriophage microarrays with attached well plate superstructure, whereby activity within wells is maintained, showing the initial bacterial attachment with genome insertion, followed by bacterial lysis, release of new virions and bacteriophage propagation. Schematics created in Biorender.com. b) TEM of JG004. Scale bar is 50 nm. c) CFU of JG004 microarrays covalently attached with EDC/NHS, compared to EDC/NHS only and plain glass substrates after 5 h (*n* = 4). Error bars calculated using standard error of the mean. Statistical analysis was conducted using unpaired T test, where ns statistical values, *P* = 0.5, and 1‐star showing significance with *P* < 0.1, 2‐star with *P* < 0.01, 3‐star with *P* <0.001 and 4‐star with *P* < 0.0001. d) OD600 absorbance from 0 – 5 h for JG004 + EDC/NHS microarrays, EDC/NHS only and plain glass substrates (*n* = 4). Error bars calculated using standard error of the mean. Statistical analysis was conducted using two‐way ANOVA followed by Sidak's post hoc test, where ns statistical values, *P* = 0.5, and 1‐star showing significance with *P* < 0.1, 2‐star with *P* < 0.01, 3‐star with *P* < 0.001 and 4‐star with *P* < 0.0001. e) XTT absorbance at 490 nm over 5 h for JG004 + EDC/NHS microarrays, EDC/NHS only and plain glass substrates (*n* = 4). Error bars calculated using standard error of the mean. Statistical analysis was conducted using two‐way ANOVA followed by Sidak's post hoc test, where ns statistical values, *P* = 0.5, and 1‐star showing significance with *P* < 0.1, 2‐star with *P* < 0.01, 3‐star with *P* < 0.001 and 4‐star with *P* < 0.0001. f) Depicts the XTT assay color difference between wells containing JG004 microarrays (light yellow), compared to wells without JG004 (dark orange), caused by the redox reactivity of XTT in the presence of bacteria (reduction from tretrazolium salt to formazan). Scale bars are 5 mm for well plate images. Error bars calculated using standard error of the mean for all graphs. Schematics created in Biorender.com.

In our study, as a proof‐of‐concept, we covalently immobilized a lytic *Pseudomonas aeruginosa (P. aeruginosa)* bacteriophage in a microarray using µCP, leveraging the amine groups present on the phage protein capsid. These microarrays contain biologically active bacteriophages that can hijack bacteria while immobilized and initiate the bacteriophage lytic cycle (**Figure** **5**a). Specifically, we immobilize a microarray containing JG004, a myophage,^[^ ^120^ ^]^ specific to PAO1 bacterial strain of *P. aeruginosa*, composed of an isometric head and a contractile tail, as shown in the transmission electron microscopy (TEM) image in Figure 5b.

Revisiting the FMEA's highest risk process steps, one challenge that arose in optimization is the mixing of EDC/NHS with JG004 in one bioink solution, as it can lead to inactivation of bacteriophages. Literature reports that EDC/NHS can block bacteriophage receptors when mixed with bacteriophages in the same bioink.^[^ ^121^ ^]^ This is due to the difference in size, morphology, function, structure, number of functional groups and bioactivity of bacteriophage compared to BSA‐FITC or monoclonal antibodies.^[^ ^122^, ^123^, ^124^, ^125^ ^]^ For instance, bacteriophages contain thousands of amine groups compared to proteins and antibodies, that have hundreds, which makes bacteriophages more sensitive to EDC/NHS chemistry.^[^ ^123^, ^124^, ^125^ ^]^ Additionally, their tail fiber receptors are much more complex structurally compared to the binding sites of antibodies.^[^ ^122^ ^]^ For the bacterial bioassay, we were able to easily translate our optimization strategies toward a separate printing approach, which showcases the versatility of our FMEA and optimization protocol. Here, substrates functionalized with carboxyl groups were incubated in EDC/NHS for 1 h before JG004 bioprinting.^[^ ^121^ ^]^

Through our industry viable risk assessment procedure, we were able to easily customize and adjust our existing protocol for fluorescent JG004, stained with SYBR‐GOLD, as described in the Experimental Section. Air‐drying time was also optimized for, similar to BSA‐FITC and antibodies, as shown in Figure S33 (Supporting Information), resulting in an ideal dry time of 12 min. EDC/NHS cross‐linking also significantly improved the JG004 microarray SNRs compared to traditional microarrays, increasing the SNR from 6.61 to 12.7 (almost 2X), achieving more uniformity in fluorescent patterns, and decreasing intraassay %CV from 16.78% to 7.10%. Traditionally, without EDC/NHS, 2 or more layers of droplet application would be necessary to achieve similar binding as EDC/NHS with one layer, showing that EDC/NHS enhanced bacteriophage immobilization as it did with BSA‐FITC (Figures S34 and S35, Supporting Information).

To assess the functionality of bacteriophage post‐printing, and after 96‐well plate adhesive superstructure application on top of the printed microarrays, PAO1 suspensions were added to each well and incubated for 5 h at 37 °C. Each well contained JG004+EDC/NHS microarrays, EDC/NHS only, or plain glass. After 5 h incubation with *P. aeruginosa* PAO1, the colony‐forming units (CFU) were lower by 3 logs in the JG004+EDC/NHS microarrays compared to controls with only EDC/NHS or plain glass substrates, showing preserved bacteriophage bioactivity (Figure 5c). This finding was also validated with optical density at 600 nm (OD_600_) measurements, showing lower turbidity because of bacteriophage‐mediated PAO1 cell lysis, resulting in decreased absorbance values over time (Figure 5d). Self‐propagation properties was also observed in the JG004 microarrays, as the plaque‐forming units per mL (PFU mL^−1^) were 4.85 × 10^11^ after 5 h, starting from an initial bacteriophage bioink concentration of 5  ×  10^9^ PFU mL^−1^ before printing. It is worth noting that the printing process results in a very thin layer of phages at a low concentration, yet significant amplification occurred over time.

Next, the bacterial metabolic activity bioassay was conducted using an 2,3‐bis‐(2‐methoxy‐4‐nitro‐5‐sulfophenyl)‐2H‐tetrazolium‐5‐carboxanilide (XTT) assay. XTT is a light‐yellow redox‐sensitive tetrazolium salt which is reduced to formazan due to cellular respiration of bacterial cells, resulting in a dark orange colour.^[^ ^115^ ^]^ After 5 h, JG004 + EDC/NHS microarrays showed significantly less metabolic activity of *P. aeruginosa* PAO1 (Figure 5e), with a light‐yellow color (Figure 5f). Meanwhile, EDC/NHS and plain glass controls were dark orange, indicating high bacterial activity after 5 h (Figure 5e,f). To note, EDC/NHS did not affect the activity of the bacteria, as no significant difference is shown in the EDC/NHS condition for both OD_600_ and XTT compared to plain glass, making it a feasible cross‐linking method for bacteriophage microarray fabrication for various bioassays.

Overall, the bioassay conducted with *P. aeruginosa* PAO1 indicates preserved bacteriophage activity after µCP evidenced by reduced bacterial concentration, metabolic activity, and increased phage concentration through self‐propagation. The distinct color difference between bacteriophage+ EDC/NHS microarrays (light yellow) and controls (dark orange), further confirms the bacteriophage activity and successful bioassay performance. By immobilization using µCP, bacteriophage microarrays can be prepared in a high throughput manner that has the potential to be implemented in industrial‐scale bioassay fabrication.

Our proof‐of‐concept platform of immobilized bacteriophage microarrays can be adapted and integrated into diagnostic portable devices for clinical screening and testing applications. This is because immobilized bacteriophages are increasingly implemented in biosensors due to their ability to selectively capture bacterial targets, enabling rapid and specific pathogen detection, even against antimicrobial‐resistant strains. When coupled with transducer platforms, such as electrodes, optical fibers, or nanomaterials, phage–bacteria interactions can be converted into measurable electrochemical, optical, or piezoelectric signals. Practical examples that our proof‐of‐concept can be expanded into include phage‐coated quartz crystal microbalance, surface plasmon resonance, and electrochemical impedance spectroscopy devices, which have demonstrated sensitive detection of viable bacteria in clinical, food, and environmental samples.^[^ ^116^, ^119^ ^]^ Beyond bacterial detection, phages can be genetically engineered to display peptides or proteins with affinity for disease‐specific biomarkers such as tumor antigens or inflammatory mediators. When immobilized on sensor surfaces, such as in the microarray fashion we present, these engineered phages act as stable and low‐cost alternatives to antibodies, enabling highly specific recognition in immunoassays. Thus, our proof‐of‐concept for holds promise for applications in cancer diagnostics, biomarker detection, and broader clinical testing where reliable and scalable biosensing tools are needed.^[^ ^126^ ^]^

Thus, our unique proof‐of‐concept design that combines both covalent carbodiimide cross‐linking and LIS addresses the key challenges of repeatability and reproducibility, marking a significant step toward automating µCP for large‐scale production of versatile biorecognition agents. To date, a fully optimized, customizable and standardized system for high‐throughput µCP has not been reported for translating lab‐scale processes to industrial manufacturing. We also achieve, for the first time, the immobilization of lytic bacteriophages into microarrays with maintained infectivity and self‐propagation properties. Our FMEA optimization process was, however, able to pinpoint high‐risk procedural steps to alleviate and reduce the time and effort needed to optimize and scale µCP. Thus, an automated µCP machine can be customizable with our developed FMEA as a guideline for µCP optimization, achieving high versatility.

## 3 Conclusion

In this work, we developed and optimized a µCP protocol using industrial risk assessment tools, being one of the first studies to do so.^[^ ^84^, ^85^, ^86^, ^87^, ^88^ ^]^ The use of a lubricant‐infused surface, in a proof‐of‐concept microarray containing immobilized BSA‐FITC, which minimized non‐specific attachment, while incorporating a cross‐linker, that enhanced the stability of biorecognition agent immobilization through covalent bonding. For this microarray, we established an optimized bioink ratio of 50:50 between cross‐linker and biorecognition agents. It was also determined that 0.25% glycerol is the most effective additive in reducing the highly common coffee ring effect, which significantly improved the SNRs of our printed lubricant‐infused microarrays. Finally, environmental factors, such as temperature, relative humidity and force application were all optimized, at 20°C, 60% relative humidity and 32.67kPa, respectively.

From this optimized protocol, we developed a preliminary model for a manufacturing process, using automated syringe droplet application and removal, as well as standardized arbor press and force sensor pressure application, with 3 min airdrying in between. Additionally, we show the applicability of our protocol and optimization approach in bioprinting microarrays with a variety of biorecognition agents. Leveraging carbodiimide cross‐linking, we successfully bioprinted BSA‐FITC, antibodies and bacteriophages, despite their different sizes and degrees of biological activity. Through FMEA optimization, we were able to confirm the preserved bacteriophage functionality by various bacterial bioassays, such as CFU, turbidity (OD_600_), and conducted a successful XTT metabolic activity measurement bioassay.

Regardless, key limitations exist in the design that will require iterative testing. Mainly, our optimized µCP protocol, while versatile for printing antibodies, bacteriophages and other immunoassay probes, will need to be slightly readjusted depending on the attributes of the biorecognition agents within the bioink. The different sizes, reactivity profiles and molecular weights these biorecognition agents possess could necessitate minor adjustments to surface functionalization, bioink incubation, dry times, and weight applications of our optimized protocol. or industrialization purposes, long‐term durability, storage stability, and leaching or contamination risks associated with fluorosilanization, need to be investigated, over weeks and months, at both room‐temperature and 2–8 °C storage conditions. Future steps of this work will be to conduct scaled up testing in industrial facilities, as well as its shelf‐life and integration into production pipelines.

## 4 Experimental Section

### Materials

The reagents and materials utilized for the experimental methods, such as surface functionalization, bioink formulation, and IFA preparation, include the following: SYLGARD 184 Silicone Elastomer Base for Polydimethylsiloxane (PDMS) (Dow Silicones Corporation, Michigan, USA), SYLGARD 184 Silicone Elastomer Curing Agent (Dow Silicones Corporation, Michigan, USA), Poly(methyl methacrylate) (PMMA) slides (McMaster University, Hamilton, ON, Canada), bovine serum albumin–fluorescein isothiocyanate conjugate (BSA‐FITC) (Sigma‐Aldrich, Oakville, ON, Canada), Glycerol, for molecular biology, ≥99% (Sigma‐Aldrich, Oakville, ON, Canada), 1‐Ethyl‐3‐(3‐dimethylaminopropyl)carbodiimide (EDC) (Sigma‐Aldrich, Oakville, ON, Canada), N‐Hydroxysuccinimide, Solid, 98%, Poly bottle (NHS) (Sigma‐Aldrich, Oakville, ON, Canada), (3‐Glycidyloxypropyl)trimethoxysilane (GLYMO), print solution: 1X PBS + BSA + sugar (McMaster University, Hamilton, ON, Canada), Reconstitution Buffer 1 (PBS) (R&D Systems, Minnesota, USA), trichloro(1H,1H,2H,2H‐perfluorooctyl) silane (TPFS) (Sigma‐Aldrich, Oakville, ON, Canada), Perfluoroperhydrophenanthrene, selectophore (PFPP) (Sigma‐Aldrich, Oakville, ON, Canada), biotinylated IL‐6 monoclonal antibody (MQ2‐39C3, anti‐IL‐6 DAb) (ThermoFisher Scientific, ON, Canada), human TNF RI/TNFRSF1A biotinylated antibody (anti‐TNF‐R1 DAb) (R&D Systems, Minnesota, USA), Streptavidin‐Cy5 (Vector Laboratories, California, USA), Quantikine ELISA Wash Buffer (WB) (R&D Systems, Minnesota, USA), general assay diluent (ImmunoChemistry Technologies, California, USA), SYBR™ Gold Nucleic Acid Gel Stain (10,000X Concentrate in DMSO) (SYBR‐GOLD) (ThermoFisher Scientific, ON, Canada), Hanks' Balanced Salt Solution (HBSS) without Ca2+, Mg2+ for Pierce™ Primary Cell Isolation Kits (HBSS) (ThermoFisher Scientific, ON, Canada), JG004 (DSMZ, Germany), *Pseudomonas Aeruginosa PAO1* (DSMZ, Germany), 2,3‐bis‐(2‐methoxy‐4‐nitro‐5‐sulfophenyl)‐2H‐tetrazolium‐5‐carboxanilide (XTT) (Sigma‐Aldrich, Oakville, ON, Canada), menadione (Sigma‐Aldrich, Oakville, ON, Canada), Luria Broth (LB) (ThermoFisher Scientific, ON, Canada), McConkey Agar (ThermoFisher Scientific, ON, Canada), Electron Microscopy Sciences 1% Uranyl Acetate Solution (ThermoFisher Scientific, ON, Canada).

### Fabrication of PDMS Stamps

To fabricate the stamps, a pre‐designed mold was created with the desired square pillar pattern. The square pillars had dimensions of 150 µm width, 150 µm length, and 1 µm height, with ‘L’ shaped landmarks to pinpoint specific areas for bioink application. The design was first modelled in Fusion 360, whereby a silicon mold with the predetermined design was fabricated utilizing photolithography. Afterward, uncured PDMS (40 mg) was mixed with a PDMS curing agent (4 mg) at a ratio of 10:1 for 20 min via stirring. The mixture was then placed under vacuum in a desiccator at −0.08 MPa pressure for 45 min to allow for degassing of the mixture. The degassed mixture was poured into the silicon mold and heated for 24 h at 60 °C, or until the mixture solidified. The stamps were removed from the mold and cut to dimensions of 1.8 mm width, 2 mm length, and 1 mm height. To clean the mold of debris, the stamps were sonicated for 5 min. The stamps were regularly imaged under brightfield using the Nikon ECLIPSE Ti2 Series Inverted Microscope to track the quality of the stamps overtime, and 10 uses were determined as the threshold of when the stamps became ‘aged’ stamps.

### Substrate Preparation and Functionalization

Before functionalization, high‐purity grade PMMA, was prepared using casting and multistage purification, creating solid sheets with thickness 1mm. These PMMA sheets have the following polymer characteristics: molar mass of ≈120 kDa, 92% visible light transmission, 1.49 refractive index, and tensile strength of 80 MPa. Each sheet was cut to 25 mm × 29 mm. The cut PMMA substrates were prepared by handwashing with soap (0.25 mL) and deionized water, carefully sprayed with 100% ethanol (0.25 mL), and blow‐dried with nitrogen gas to remove any streaks or impurities. Then the PMMA slides were CO_2_ – plasma treated for 15‐min for activated carboxyl group deposition using a PlasmaEtch PE‐100.

### Surface Characterization

For each stage of functionalization, surface characterization was conducted on the PMMA. First, contact angle measurements were collected using the Kruss Drop Shape Analyzer DSA30S, to capture images of and assess the hydrophilicity of the functionalized PMMA. Five microliters droplets of water were introduced on multiple surface locations of plain, CO_2_ plasma‐treated, printed, fluorosilanized, and printed followed by fluorosilanized PMMA slides. High‐resolution XPS was also performed, using a PHI Quantera II, at the Biointerfaces Institute, McMaster University, to confirm the chemical bonds on the surface of plain, CO_2_ plasma‐treated and fluorosilanized PMMA slides. C1s peaks were recorded (see Figures S4–S6, Supporting Information) and deconvoluted using the CasaXPS application (see Figures S7 – S9 and Table S1, Supporting Information).

### Bioink Preparation

The bioink used in this study was composed of the following: print solution (or PBS for control) glycerol, EDC/NHS and either BSA‐FITC or DAb. First GLYMO epoxy or glycerol was serially diluted in print solution or PBS. Then EDC was measured at 5 mg (± 0.3 mg) and NHS was measured at 3 mg (± 0.3 mg). The EDC/NHS combination was then dissolved in solution of glycerol and print solution (125 µL). After each of the mentioned steps, the solutions were mixed via vortex at maximum speed for 10–20 s. Finally, the EDC/NHS solution was mixed with BSA‐FITC, also diluted in print solution, with a stock concentration of 3 mg mL^−1^ during the optimization tests. For anti‐IL‐6 and/or anti‐TNF RI DAb, they were prepared at a concentration of 200 µg mL^−1^. The final solution, containing either BSA‐FITC or DAb was mixed via pipette. In the separate printing test, EDC/NHS and BSA‐FITC were not mixed in this method, but individually printed, either immediately after one another or with a 1, 2, 3, or 4 min incubation between separate prints.

### Immobilization of Biorecognition Agents through µCP

The bioink was applied to each PDMS stamp within the ‘L’ shape of the two landmark regions at 5 µL volume per droplet, applied manually via pipette. The droplets were incubated for 2 min to allow for the bioink to be absorbed into the PDMS pillar shapes, before removal with pipette, Kimwipe or centrifugal force. The excess bioink was air dried in which during optimization and air‐drying times were assessed at different controlled humidity environments. After air drying, the stamp was flipped and placed on the functionalized PMMA substrate, and a weight was manually applied to facilitate physical transfer of the bioink from the PDMS stamp to the PMMA substrate. After 1 min of weight application, the weight and stamp were removed, and the PMMA substrates incubated in a controlled ≈100% relative humidity environment for 1‐h. CVD using TPFS was then performed for 15 min to allow for fluorosilanization of the printed PMMA substrates. Wells were created on the substrates using a 16‐well superstructure assembly, whereby PFPP lubricant was applied, and three consecutive 10 min washes were conducted, controlling relative humidity of ≈100%. For the commonly reported µCP protocols, the substrate was functionalized with 5 min O_2_ (instead of 15 min CO_2_ plasma treatment), and only BSA‐FITC was incorporated into the bioink (EDC/NHS was excluded). The process of printing and the lubricant‐infusion via fluorosilanization was performed on these physically attached microarrays, to compare them to the dually immobilized microarrays created by the protocol. Three washes with Quantikine ELISA Wash Buffer were conducted for all conditions.

### Profilometry

A Wyko NT1100 Profilometer was utilized for mapping the rough surface of the printed substrates. The system was calibrated with a modulation threshold of 3%, at vertical scanning interferometry mode. The parameters set were at 368 × 240 mm window size for imaging all 9 squares, with 0.00 mm sampling. Images were taken at 5X objective, and X/Y profile graphs as well as 3D projections were acquired.

### SNR and CV Calculations

A Nikon ECLIPSE Ti2 Series Inverted Microscope was utilized for imaging of the results. Afterward, ImageJ software was utilized to quantify the signal and relative background noise of 9 individual square replicates for each well printed. The SNR of each square was calculated by dividing signal over noise, and a mean average was calculated among all 9 squares, or replicates, referred to as the intra‐assay SNR. Averages were taken of the intra‐assay SNR for all wells printed from each stamp to determine the inter‐assay SNR. Similarly, an intra‐assay %CV was calculated for each well, which had 9 replicates each, and the inter‐assay %CV was quantified by calculated %CV among all wells from a single stamp. Equation (1) was utilized for the calculation of these %CVs.

(1) $$\%CV=\frac{standard\;deviation\;of\;each\;SNR\;value}{average\;SNR}\left(100\%\right)$$

### Optimization of Protocol

To optimize the µCP protocol, an FMEA was utilized to assess the risk of failure at each experimental step. Each step was evaluated based on the severity of the failure in impacting the results, the frequency of failure occurrence, and the difficulty of detecting failure upon occurrence, by which the product of these factors determined the overall RPN. Optimization of BSA‐FITC targeted the high‐risk steps in the protocol determined by the FMEA. For the bioink preparation, concentrations of glycerol, EDC/NHS and BSA‐FITC were assessed. 0%, 0.1%, 0.25%, and 0.5% glycerol concentrations, as well as EDC values of 4.5, 5, and 5.5 mg, and NHS values of 2.5, 3, and 3.5 mg, were all assessed as contents of the bioink. The ratio of EDC/NHS: BSA‐FITC solution was also varied by ratios of 75:25, 50:50, and 25:75, to determine ideal ratio for chemical cross‐linking and attachment. For removal of droplets, pipette removal was assessed compared to Kimwipe and centrifuge removal. Finally, for air drying step, various dry times were assessed, including 1, 2, 3, and 4 min at different humidity‐controlled environment conditions of 20%, 40%, 60%, and 80%. Furthermore, weight application after air drying and amount of weight, between 0–1.5 kg, were also assessed to determine ideal amount of force for physical bioink transfer. These weights were converted from kg to kPa using Equation (2). Fluorescence microscopy was conducted under FITC at exposure 300 ms for look‐up tables of 100–350. SNR, statistical analyses and CVs were also calculated for each optimization. For the commonly reported µCP protocols, the substrate was functionalized with 5 min O_2_ (instead of 15 min CO_2_ plasma treatment), and only BSA‐FITC was incorporated into the bioink (EDC/NHS was excluded). The process of printing and the lubricant‐infusion via fluorosilanization was performed on these physically attached microarrays, to compare them to the dually immobilized microarrays created by the protocol. Three washes with Quantikine ELISA Wash Buffer were conducted for all conditions. Bacteriophages bioinks were similarly optimized via FMEA. For bacteriophages this resulting in a specialized µCP protocol involving 5 min droplet incubation on stamps using a cover slip, followed by a 12 min dry time and 3 min weight application time with 0.5 kg.

(2) $$Pressure\;Applied\;\left(kPA\right)=\frac{Weight\;\left(kg\right)*9.8\frac{m}{s^{2}}}{Area\;of\;Stamp*1000}$$

### Automation and Standardization of µCP Protocol

Automation of the µCP process focused on the droplet application and removal process as well as the force application process. First, a droplet application dispensing mechanism was modelled in Fusion 360 3D modelling software, and 3D printed with resin using the B9 Core 530 3D Printer. The outer diameter for the droplet dispensing apparatus was 2.5 mm, and the inner syringe diameter was 1.5 mm, with 1mm thick walls. The apparatus was connected to a NE‐1600 Six Channel Programmable Syringe Pump via tubing. Through this method, the syringe pump was programmed to dispense and pick up 5 µL of bioink, replacing the pipette in the manual method. Furthermore, a 0.5 Inch Dia FSR402 Resistive Thin Film Pressure Sensor (Force Sensing Resistor 0–10 kg) and Arduino Uno R3 were utilized to measure the exact force application placed on each stamp. This force sensor was connected to a HHIP 8600‐0031 Heavy Duty Arbor Press (0.5‐ton capacity, 10″ height) to standardize the method of force application to prevent double printing of the stamp, which could occur in the manual protocol.

### Bacterial Culture and Bacteriophage Propagation

To prepare for the bioassay, frozen samples of *P. aeruginosa* PAO1 were prepared by inoculating LB media (3 mL) containing glycerol (25% v/v). These samples were then placed in a shaking incubator overnight to promote the growth of *P. aeruginosa* PAO1, at 37 °C and 180 rpm. These *P. aeruginosa* PAO1 stocks were stored at −80 °C for use in the bacterial assay, as well as utilized in phage propagation. In bacteriophage propagation, 10 µL of JG004 stock solution was added to *P. aeruginosa* PAO1 stock, which had been allowed to reach mid‐exponential growth. The combined solution of *P. aeruginosa* PAO1 and JG004 was again incubated at 37 °C and 180 rpm, for 6 h, to ensure lysis and release of bacteriophages. Bacteriophages were removed from the lysate solution, first by centrifugation at 7000 rcf for 20 min, followed by sterilization through a filter with 0.2 µm pores. The result was a purified JG004 solution at 10^10^ PFU mL^−1^, which was stored at 4 °C for use in the bioassay.

### Bacterial Detection Bioassay with Bacteriophage Microarrays

In preparing the bioink, the 10^10^ PFU mL^−1^ concentration of non‐stained JG004 was diluted in 0.25% glycerol (in a 1:1 ratio) for the bacteriophage bioink. For bacteriophage optimization with SNR, JG004 was fluorescently stained with SYBR‐GOLD (diluted in HBSS) as per Bichet et al.’s protocol.^[^ ^127^ ^]^ The following modifications were made: droplet volume was increased from 5 to 10 µL for increased number of virions, droplet incubation increased from 2 to 5 min, to allow successful absorption into PDMS stamps, and weight from 1 to 0.5 kg was adjusted to prevent damage to the bacteriophage structure. SNRs were adjusted for 450 – 900 LUT and 300 ms exposure for this optimization. For the bioassay, JG004 was not stained. This JG004 bioink containing 0.25% glycerol was printed, in two layers, onto carboxyl functionalized glass substrates that had undergone 1 h incubation in EDC/NHS solution (as per protocol established by Hosseinidoust et al.).^[^ ^121^ ^]^ Substrates only incubated in EDC/NHS and plain glass slides were used for comparison. Afterward, a Grace Bio‐Labs ProPlate MP™ microtiter plate superstructure was added on top of the substrates to form wells. A previously prepared overnight culture of *P. aeruginosa* PAO1 bacterial stock was also diluted, in a 1 to 100 ratio, in fresh LB media, and incubated for 1hr in shaking incubator at 37 °C and 180 rpm. Hundred microliters of the subculture of *P. aeruginosa* PAO1 was added to the wells, followed by 100 µL of XTT solution, composed of final concentrations of 0.2 mg mL^−1^ for XTT salt and 0.1 mm for menadione. For OD measurements the solution composed of 100 µL of the subculture of *P. aeruginosa* PAO1 and 100 µL of fresh LB. Measurements of absorbance at 490 and 600 nm were taken in a Synergy Neo2 BioTek plate reader set at 37  °C (*n* = 3), which collected data every 5 min over a 5 h period, to determine XTT and OD_600_. LB media absorbance was subtracted from these values. Distinct color differences of the wells were captured via iPhone in an in‐house designed light‐controlled chamber. Samples were collected from the wells after 5 h and were then plated on gram negative selective McConkey agar plates to determine CFU. Phage overlay, as described by Green & Sambrook, was utilized to measure PFU, which was then averaged.^[^ ^128^ ^]^

### Transmission Electron Microscopy

JG004, at titers of 10^9^ PFU mL^−1^, was absorbed and applied onto copper grids coated with carbon, which had undergone plasma cleaning. 1% uranyl acetate was then used to negatively stain JG004. The grids containing JG004, were then air dried and imaged using a Talos L120C microscope.

### Statistical Analysis

Statistical analyses for all graphs and figures were conducted using ImajeJ and GraphPad software. The following statistical analyses were used throughout the manuscript: 1) two‐way analysis of variance (ANOVA) (or mixed effects analysis with Geiser – Greenhouse corrections) followed by Sidak's post hoc analysis where nonsignificant (ns) statistical values, *P* = 0.5, and 1‐star showing significance with *P* < 0.1, 2‐star with *P* < 0.01, 3‐star with *P* < 0.001 and 4‐star with *P* < 0.0001; 2) unpaired T tests for separately comparing conditions, where ns statistical values, *P* = 0.5, and 1‐star showing significance with *P* < 0.1, 2‐star with *P* < 0.01, 3‐star with *P* < 0.001 and 4‐star with *P* < 0.0001 (specifically for CFU results); 3) Mann‐Whitney U test, ns statistical values, *P* = 0.5, and 1‐star showing significance with *P* < 0.1, 2‐star with *P* < 0.01, 3‐star with *P* < 0.001 and 4‐star with *P* < 0.0001; and 4) one‐way ANOVA (or non‐parametric/mixed Brown‐Forsythe and Welch ANOVA model) followed by Tukey's post hoc test, ns statistical values, *P* = 0.5, and 1‐star showing significance with *P* < 0.1, 2‐star with *P* < 0.01, 3‐star with *P* < 0.001 and 4‐star with *P* < 0.0001. Contact angle measurements and profilometry had 9 replicates for each condition (*n* = 9), XPS raw data had 3 replicates per condition (*n* = 3), SNRs had 9, 12, or 18 replicates per condition (*n* = 9, 12, and 18), except automation which had 54 replicates per condition (*n* = 54), and bacteriophage bioassays, specifically OD_600_, CFU and XTT had 4 replicates per condition (*n* = 4). Error bars were calculated using either mean of standard deviation or standard error of the mean. Statistical analysis information, number of replicates, and error calculation method for each figure were added to the captions.

## Conflict of Interest

The authors declare no conflict of interest.

## Supporting information



Supporting Information

## Data Availability

The initial data that support the findings of this study are openly available in ChemRxiv at https://chemrxiv.org/engage/chemrxiv/article‐details/66fd8a3212ff75c3a1359c45, reference number https://doi.org/10.26434/chemrxiv‐2024‐hgwv3.
