# Supplementary material for: Automated Covalent Microcontact Bioprinting of Lubricant‐Infused Microarrays for Biological Assays
Source: Small. 2025 Oct 22;21(49):e06732. doi: 10.1002/smll.202506732 (PMC12696803; doi:10.1002/smll.202506732)
Supplement: Supplementary file 1 — Supporting Information [file SMLL-21-e06732-s001.docx]

**Supporting Information**

**Automated Covalent Microcontact Bioprinting of Lubricant-Infused Microarrays for Biological Assays**

Lubna Najm, Amid Shakeri^*^, Fereshteh Bayat, Shaghayegh Moghimikandelousi, Akansha Prasad, Liane Ladouceur, Samantha Dacalos, Sakina Hussain, Inaam Chattha, Hareet Sidhu, Zeinab Hosseinidoust, Tohid F. Didar^*^

L. Najm, F. Bayat, L. Ladouceur, T.F. Didar: Department of Mechanical Engineering, McMaster University, 1280 Main Street West, Hamilton, Ontario L8S 4L7, Canada

A. Shakeri: Institute of Biomedical Engineering, University of Toronto, Toronto, Ontario, M5S 3G9; Canada

S. Moghimikandelousi, A. Prasad, Z. Hosseinidoust, T.F. Didar,: School of Biomedical Engineering, McMaster University, 1280 Main Street West, Hamilton, Ontario L8S 4L7, Canada

A. Prasad, Z. Hosseinidoust: Department of Chemical Engineering, McMaster University, 1280 Main Street West, Hamilton, Ontario L8S 4L7, Canada

S. Dacalos, S. Hussain, I. Chattha, H. Sidhu: Department of Biochemistry and Biomedical Sciences, McMaster University, 1280 Main Street West, Hamilton, Ontario L8S 4K1, Canada

Z. Hosseinidoust, T.F. Didar: Institute for Infectious Disease Research (IIDR), 1280 Main St W, McMaster University, Hamilton, Ontario L8S 4L8, Canada

Z. Hosseinidoust: Farncombe Family Digestive Health Research Institute, McMaster University, 1280 Main St W, Hamilton, Ontario L8S 4L8, Canada

* Address correspondence to [didart@mcmaster.ca](mailto:didart@mcmaster.ca) and amid.shakeri@utoronto.ca


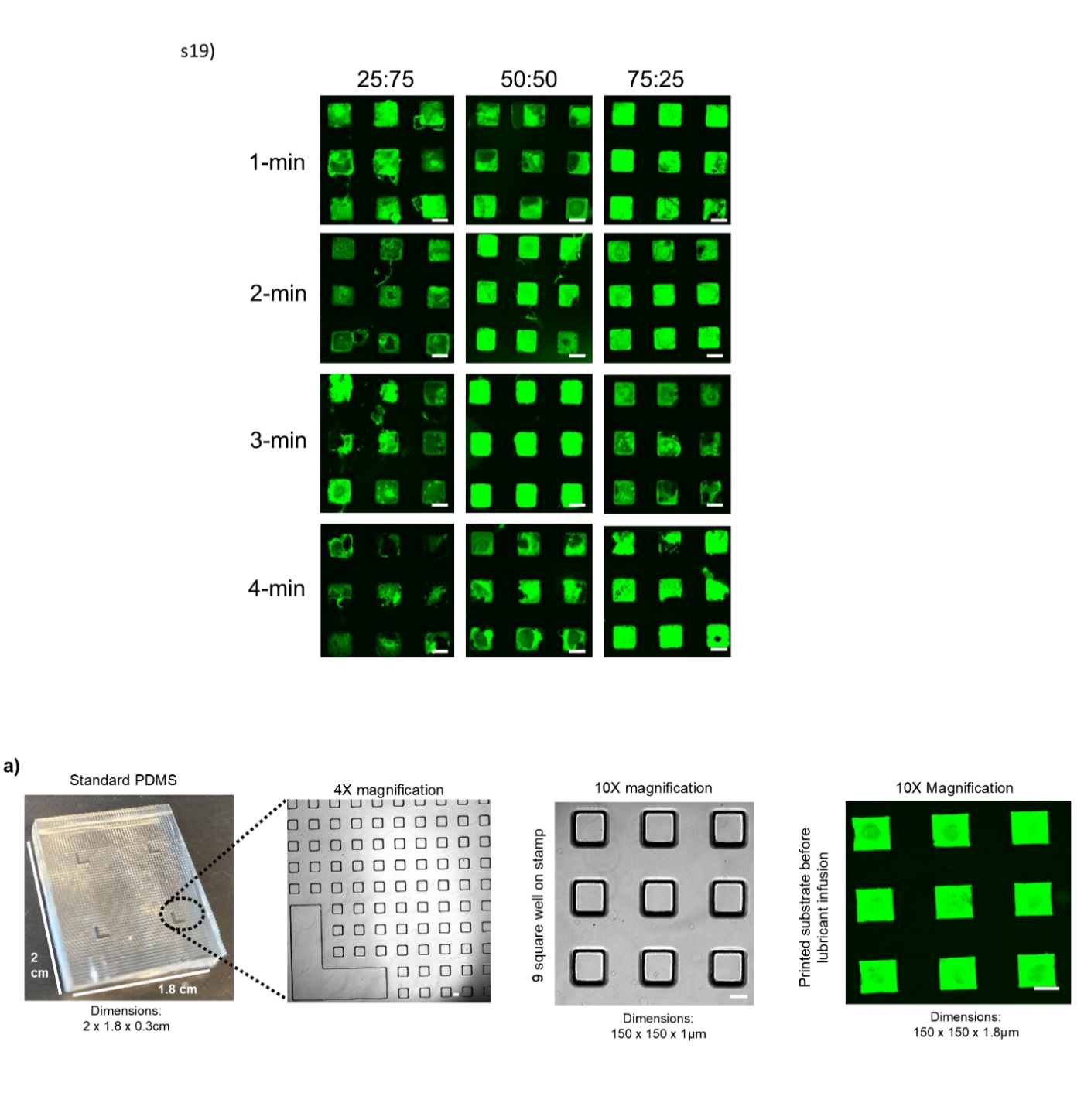


**Figure S1.** Dimensions of the PDMS stamps and individual features, imaged via brightfield microscopy, as well as final printed substrate, imaged via fluorescence microscopy. Scale bars were 100 µm.

**
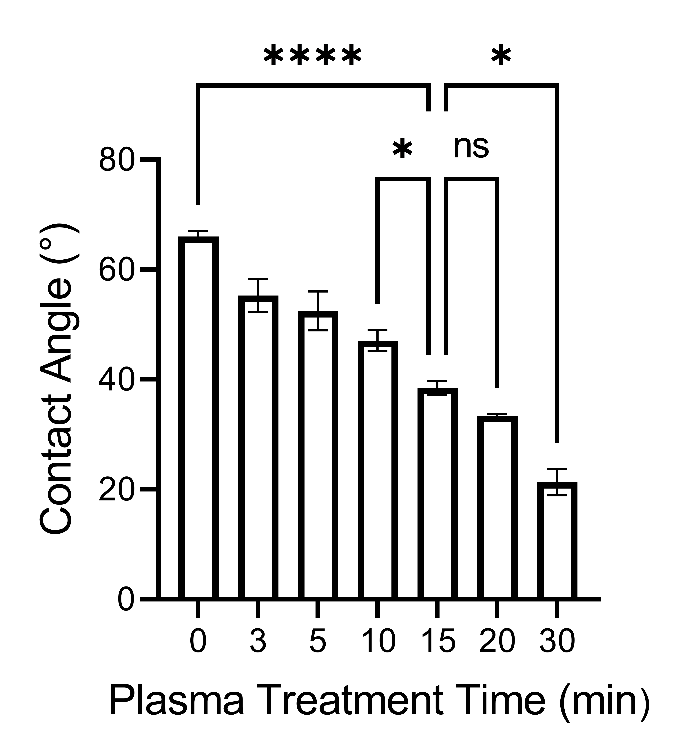
**

**Figure S2.** Depicts contact angle measurements as CO_2_ plasma treatment time increases (n = 3). Error bars calculated using the mean of standard deviation. Statistical analysis was conducted using one-way ANOVA followed by Tukey’s post hoc test, with nonsignificant (ns) statistical values where P = 0.5, and 1-star showing significance with P<0.1, 2-star with P<0.01, 3-star with P <0.001 and 4-star with P<0.0001.


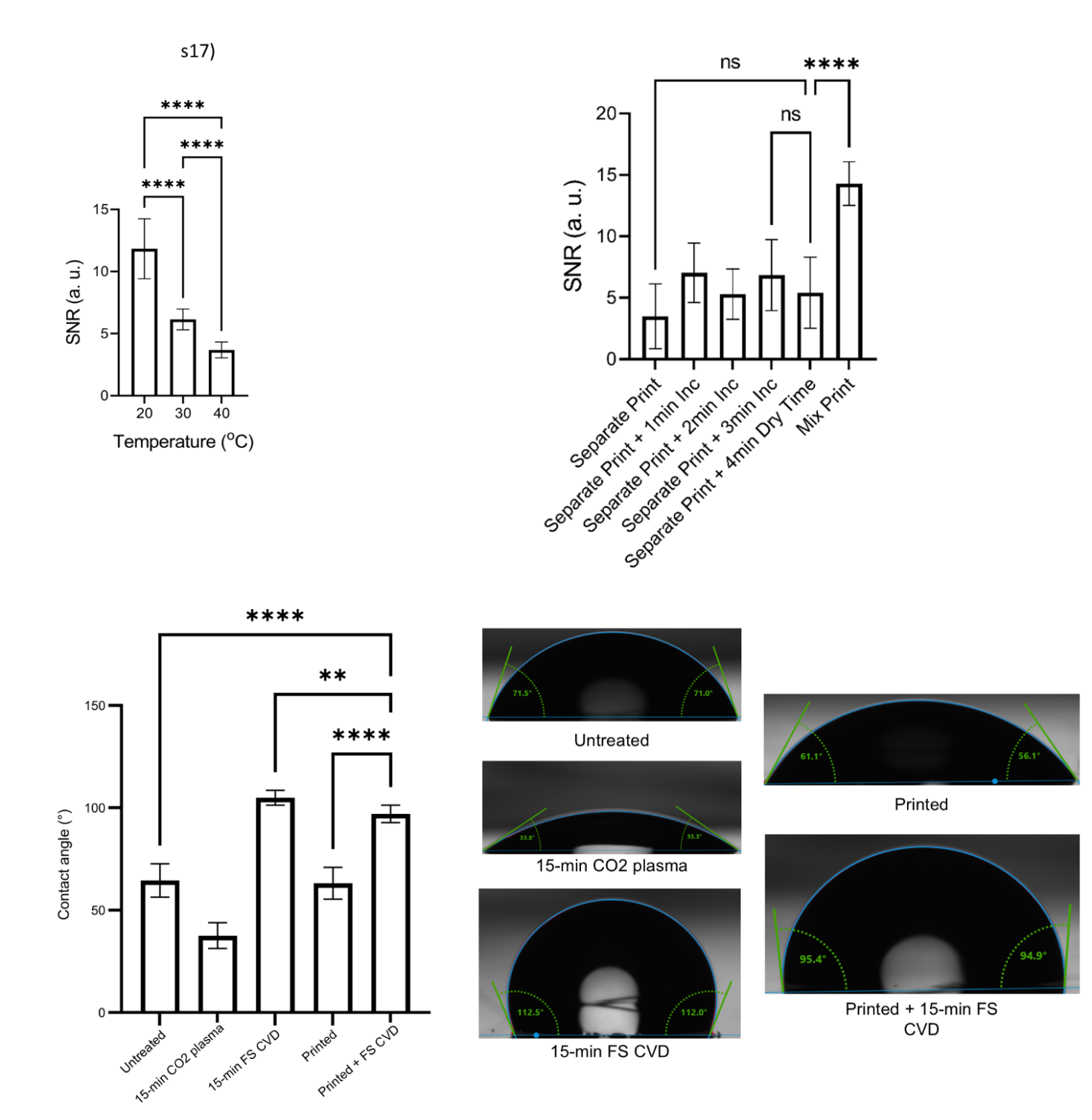


**Figure S3.** Depicts contact angle measurements and sample droplet images of PMMA slides that are untreated, 15-min CO_2_ plasma treated, 15-min FS CVD treated, printed, and printed followed by 15-min FS CVD treatment (n = 9). Error bars calculated using the mean of standard deviation. Statistical analysis was conducted using one-way ANOVA followed by Tukey’s post hoc test, with nonsignificant (ns) statistical values where P = 0.5, and 1-star showing significance with P<0.1, 2-star with P<0.01, 3-star with P <0.001 and 4-star with P<0.0001.


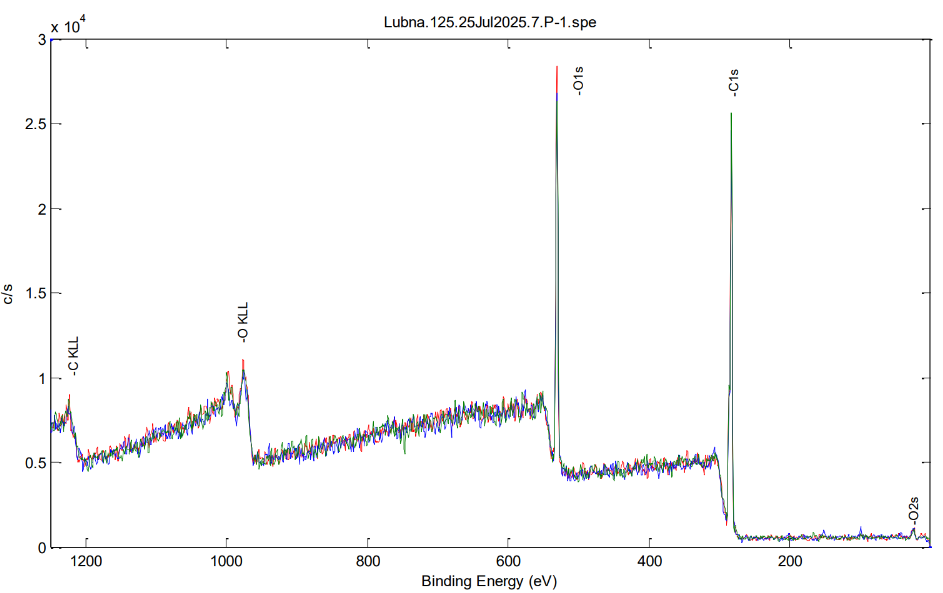


**Figure S4.** Depicts experimental XPS data for PMMA slides that are untreated (n = 3).


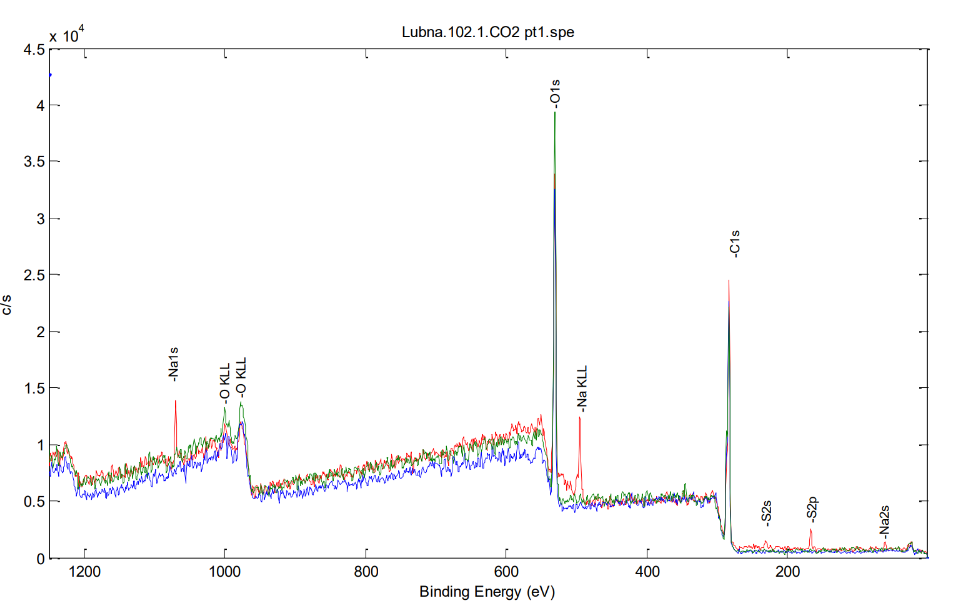


**Figure S5.** Depicts experimental XPS data for PMMA slides treated with 15-min CO_2_ plasma (n = 3).


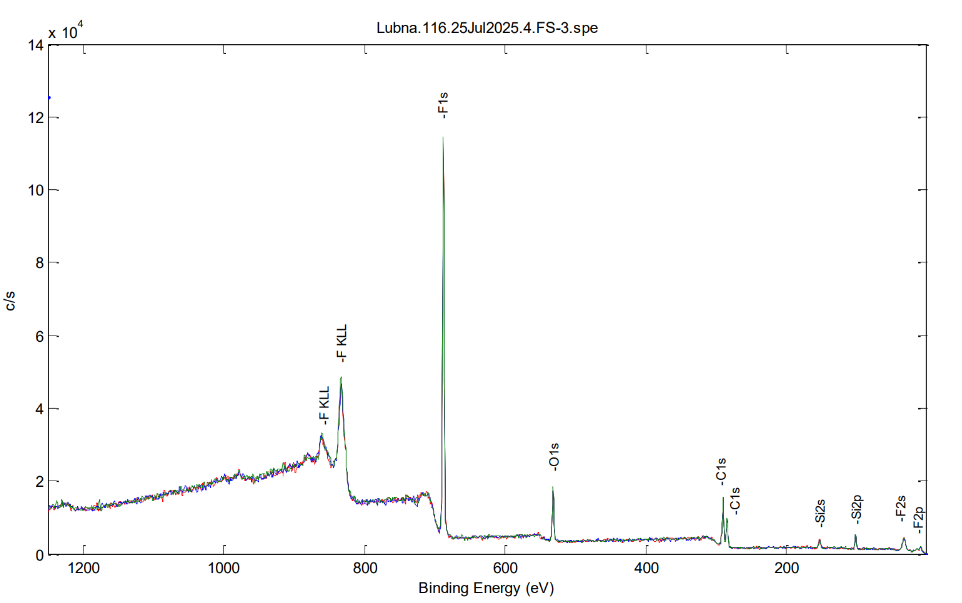


**Figure S6.** Depicts experimental XPS data for PMMA slides were treated with 15-min CO_2_ plasma, followed by 15-min FS CVD (n = 3).

**Figure S7.** Depicts sample high resolution, deconvoluted XPS data for untreated PMMA slides.

**Figure S8.** Depicts sample high resolution, deconvoluted XPS data for PMMA slides that are 15-min CO_2_ plasma treated.

**Figure S9.** Depicts sample high resolution, deconvoluted XPS data for PMMA slides were treated with 15-min CO_2_ plasma, followed by 15-min FS CVD.


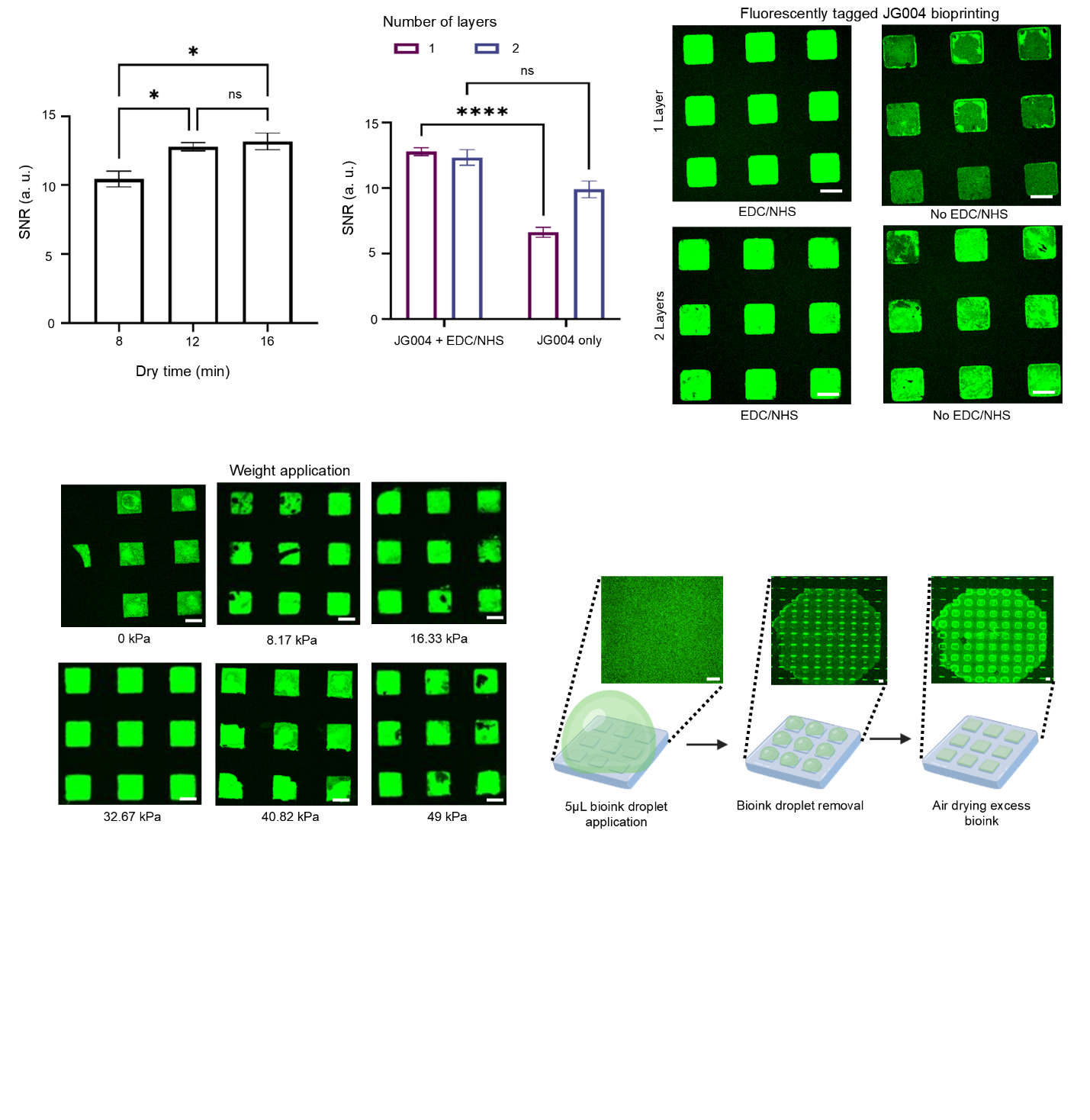


**Figure S10.** Depicts the application of a 5µL bioink droplet, followed by droplet removal and air drying to remove excess bioink, with associated fluorescence images. Scale bars were 100 µm. Schematics created in Biorender.com.

**
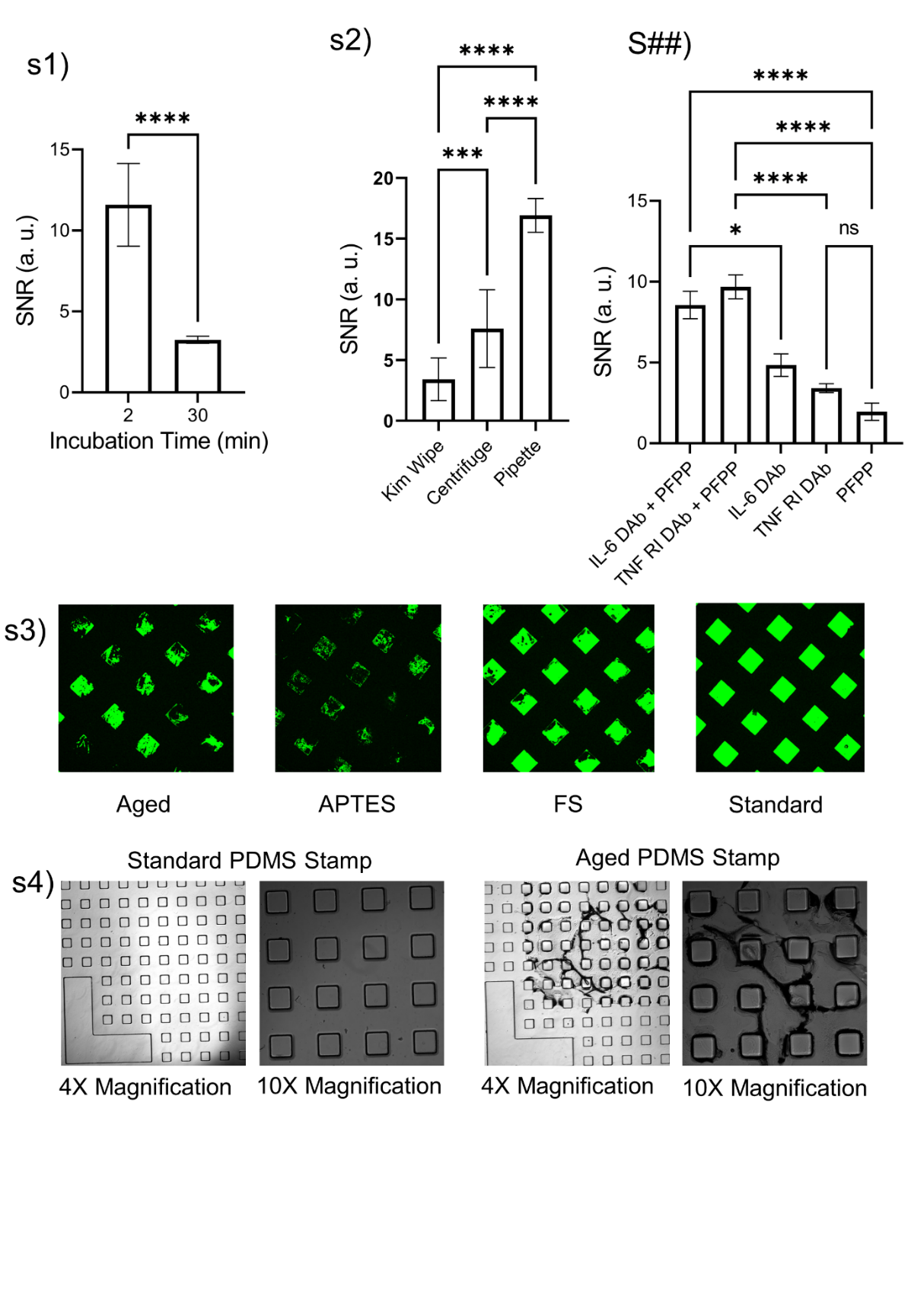
**

**Figure S11.** SNRs after µCP where 2-minute droplet incubation vs. 30-minute droplet incubation were assessed, before droplet removal (n = 18). Error bars calculated using the mean of standard deviation. Statistical analysis was conducted using Mann-Whitney U test, nonsignificant (ns) statistical values, P = 0.5, and 1-star showing significance with P<0.1, 2-star with P<0.01, 3-star with P <0.001 and 4-star with P<0.0001.


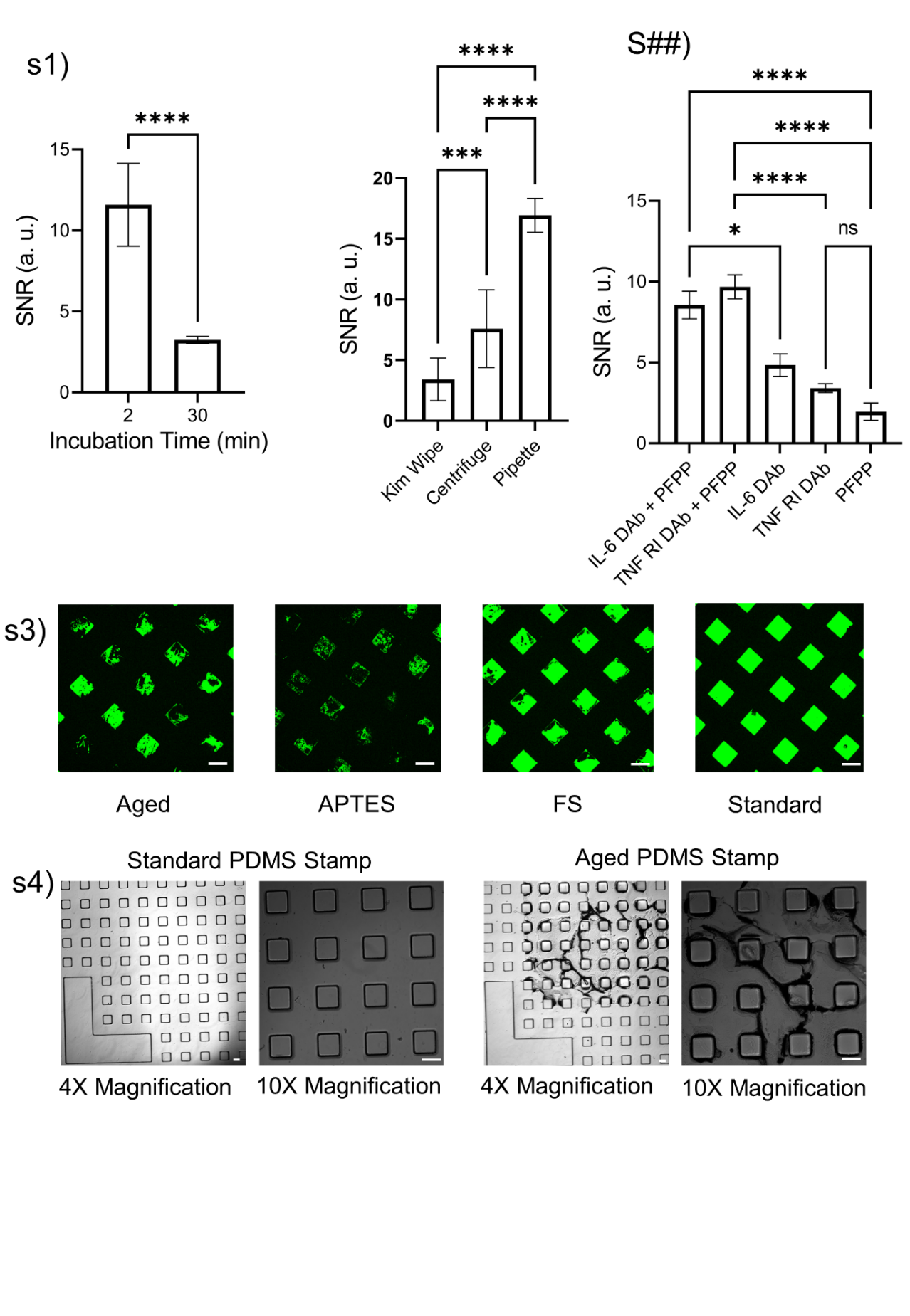


**Figure S12.** Brightfield images of new standard PDMS stamps compared to aged PDMS stamps after 10 uses, at 4X and 10X magnification. Landmark ‘L’ is shown in the 4X images. Scale bars were 100 µm.


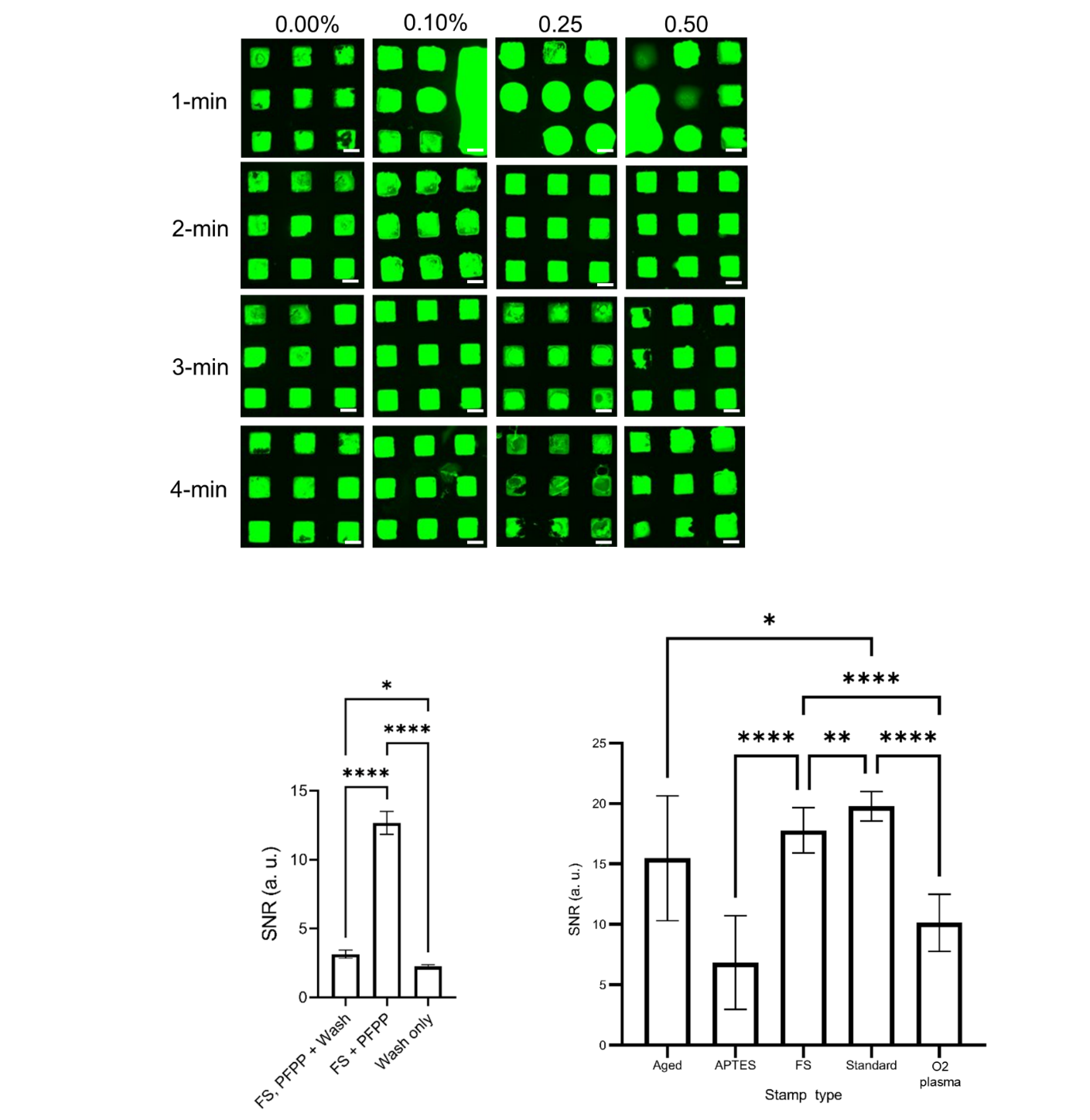


**Figure S13.** SNRs after µCP with aged, APTES-treated, FS-treated, standard and stamps (n=18). Error bars calculated using the mean of standard deviation. Statistical analysis was conducted using one-way ANOVA followed by Tukey’s post hoc test, with nonsignificant (ns) statistical values where P = 0.5, and 1-star showing significance with P<0.1, 2-star with P<0.01, 3-star with P <0.001 and 4-star with P<0.0001.


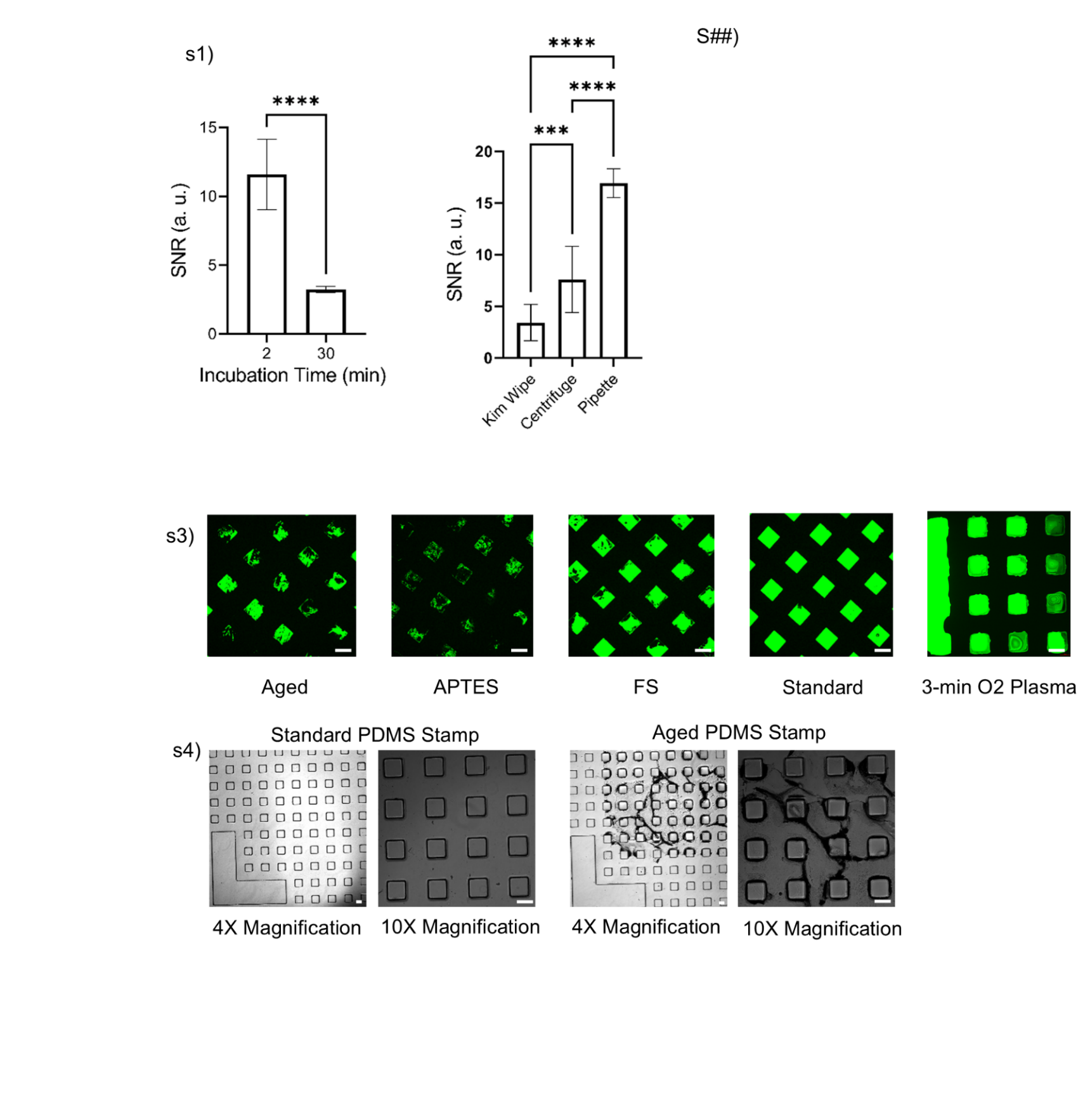


**Figure S14.** Fluorescence images, at 10X magnification, of printed substrates after µCP with aged, APTES-treated, FS-treated, new standard untreated, and 3-min O_2_ plasma treated stamps. Scale bars were 100 µm.


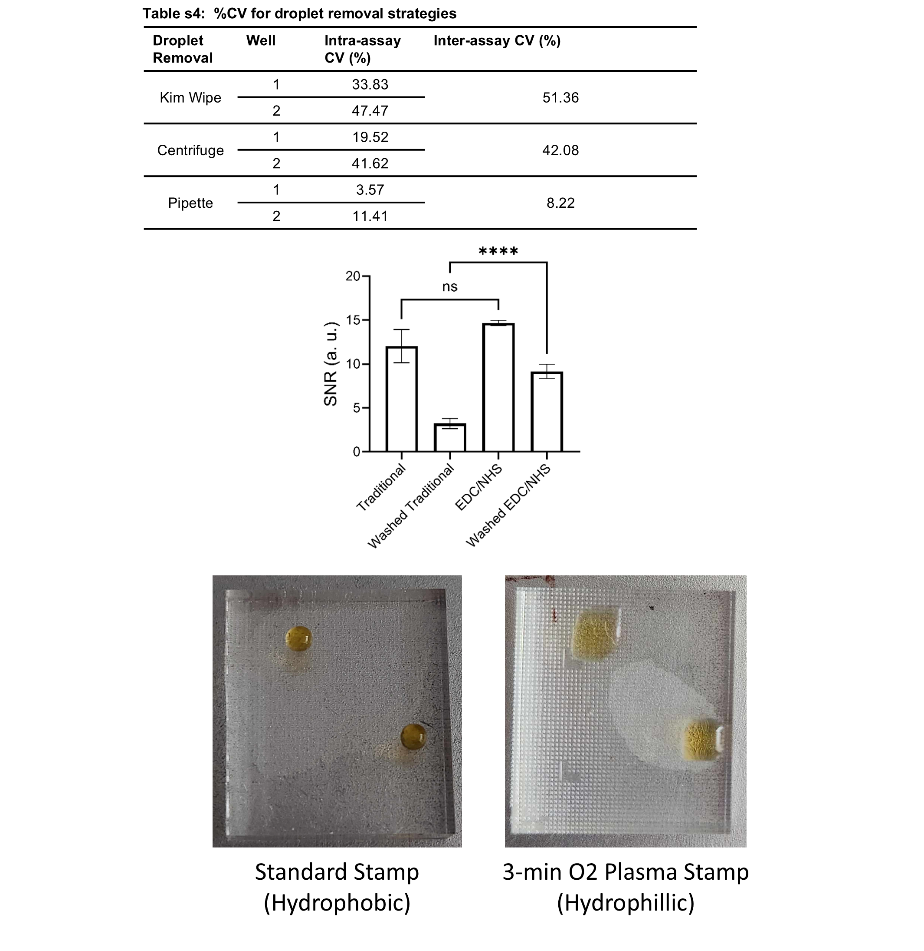


**Figure S15.** Images of droplets incubating on a standard stamp *vs* 3-min O_2_ plasma treated stamp.


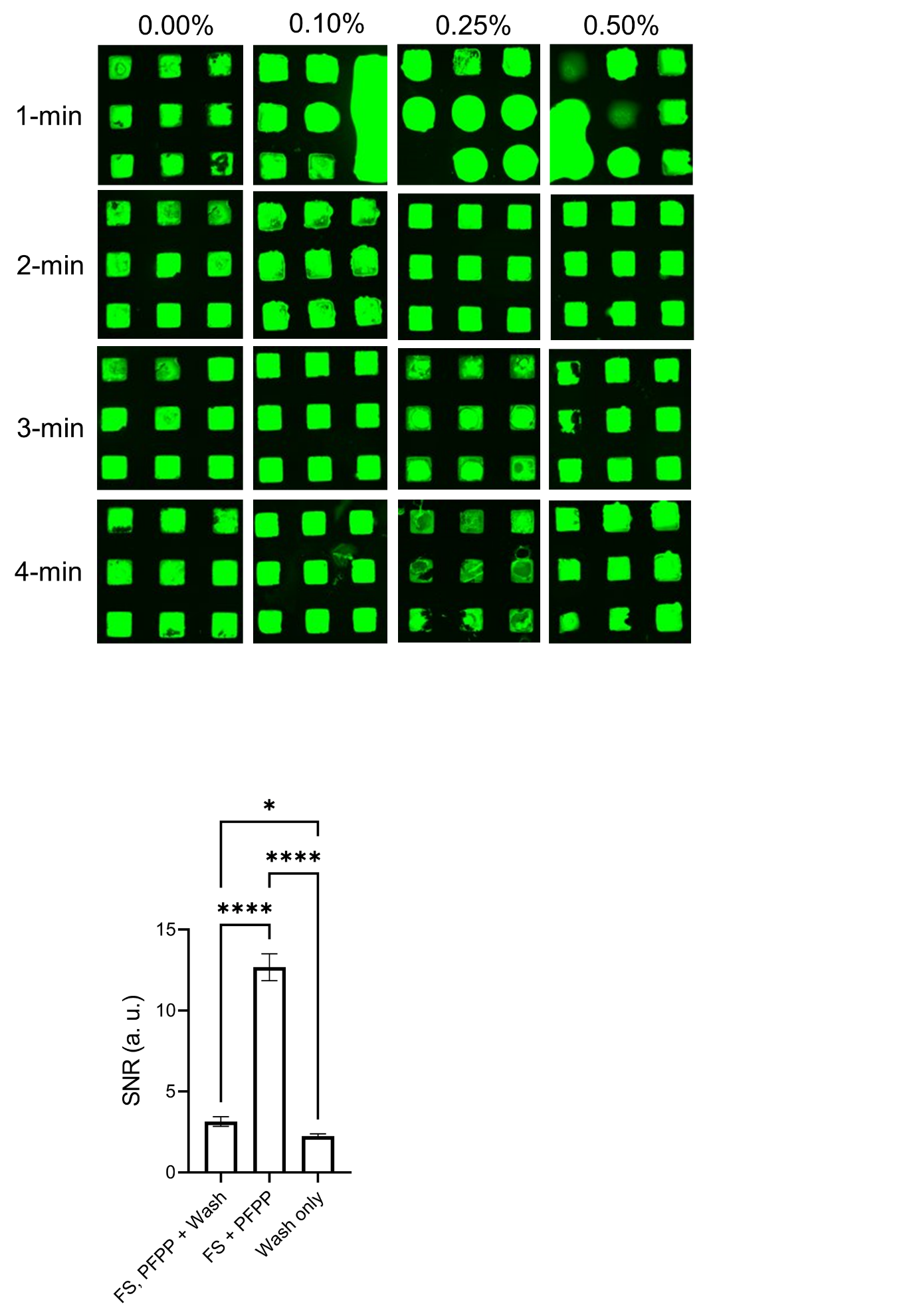


**Figure S16.** SNRs after µCP of BSA-FITC comparing the effects of lubrication, washing and a combination of both (n = 18). Error bars calculated using standard error of the mean. Statistical analysis was conducted using one-way ANOVA followed by Tukey’s post hoc test, with nonsignificant (ns) statistical values where P = 0.5, and 1-star showing significance with P<0.1, 2-star with P<0.01, 3-star with P <0.001 and 4-star with P<0.0001.


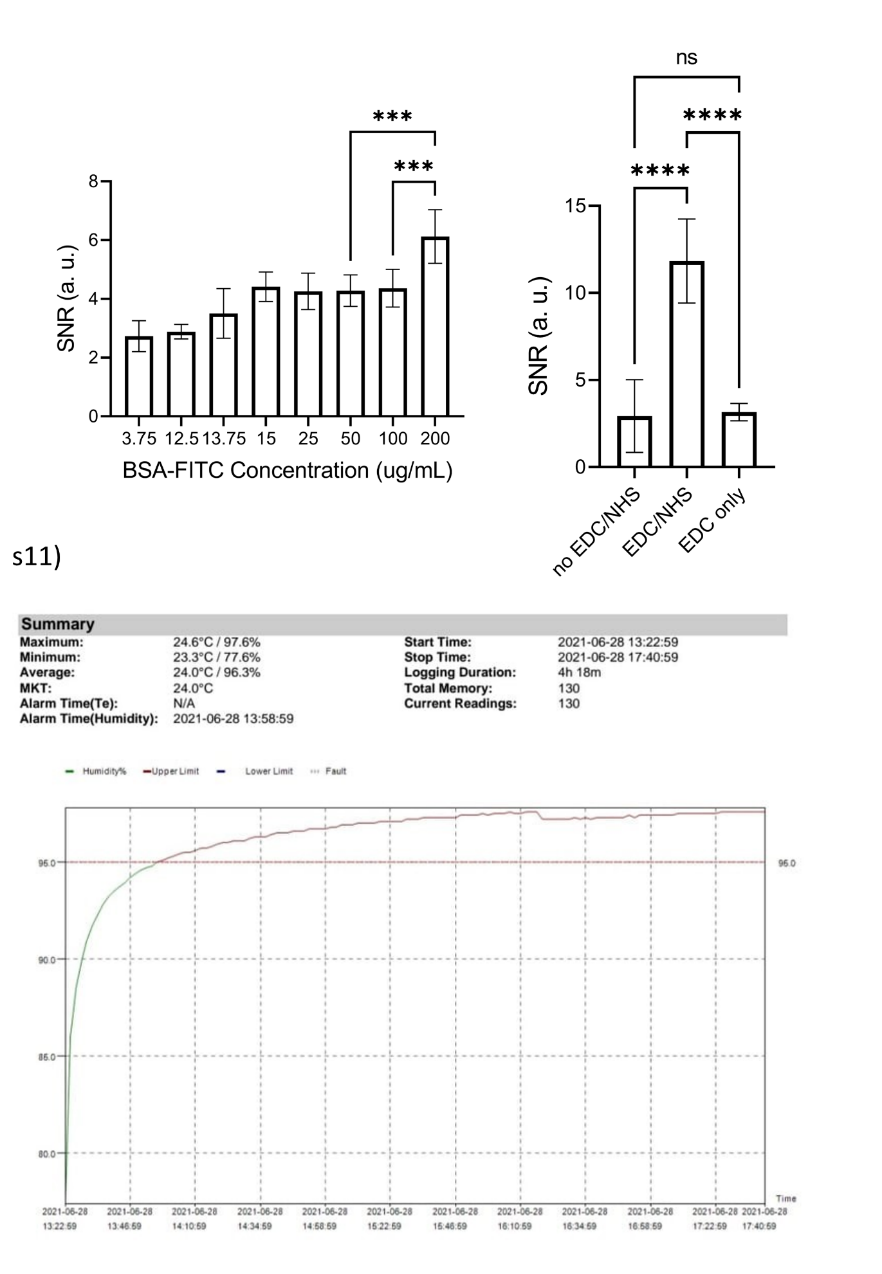


**Figure S17.** SNRs after µCP of BSA-FITC comparing no crosslinker, EDC/NHS combined cross linker and only EDC crosslinker (n = 18). Error bars calculated using the mean of standard deviation. Statistical analysis was conducted using one-way ANOVA followed by Tukey’s post hoc test, with nonsignificant (ns) statistical values where P = 0.5, and 1-star showing significance with P<0.1, 2-star with P<0.01, 3-star with P <0.001 and 4-star with P<0.0001.


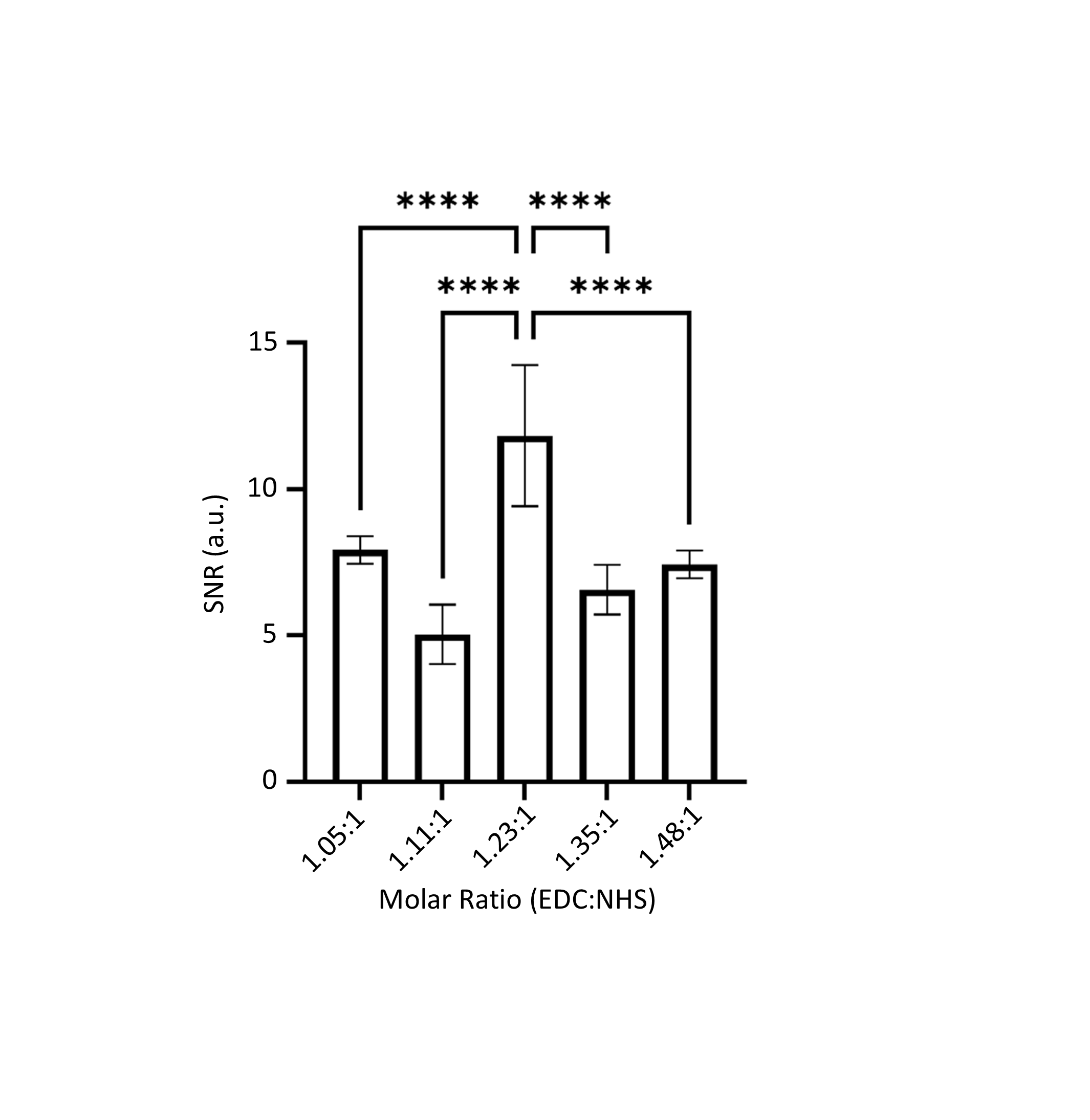


**Figure S18.** SNRs after µCP of BSA-FITC at EDC:NHS molar ratios of 1.05:1, 1.11:1, 1.23:1, 1.35:1, and 1.48:1 (n = 18). Error bars calculated using the mean of standard deviation. Statistical analysis was conducted using one-way ANOVA followed by Tukey’s post hoc test, with nonsignificant (ns) statistical values where P = 0.5, and 1-star showing significance with P<0.1, 2-star with P<0.01, 3-star with P <0.001 and 4-star with P<0.0001.


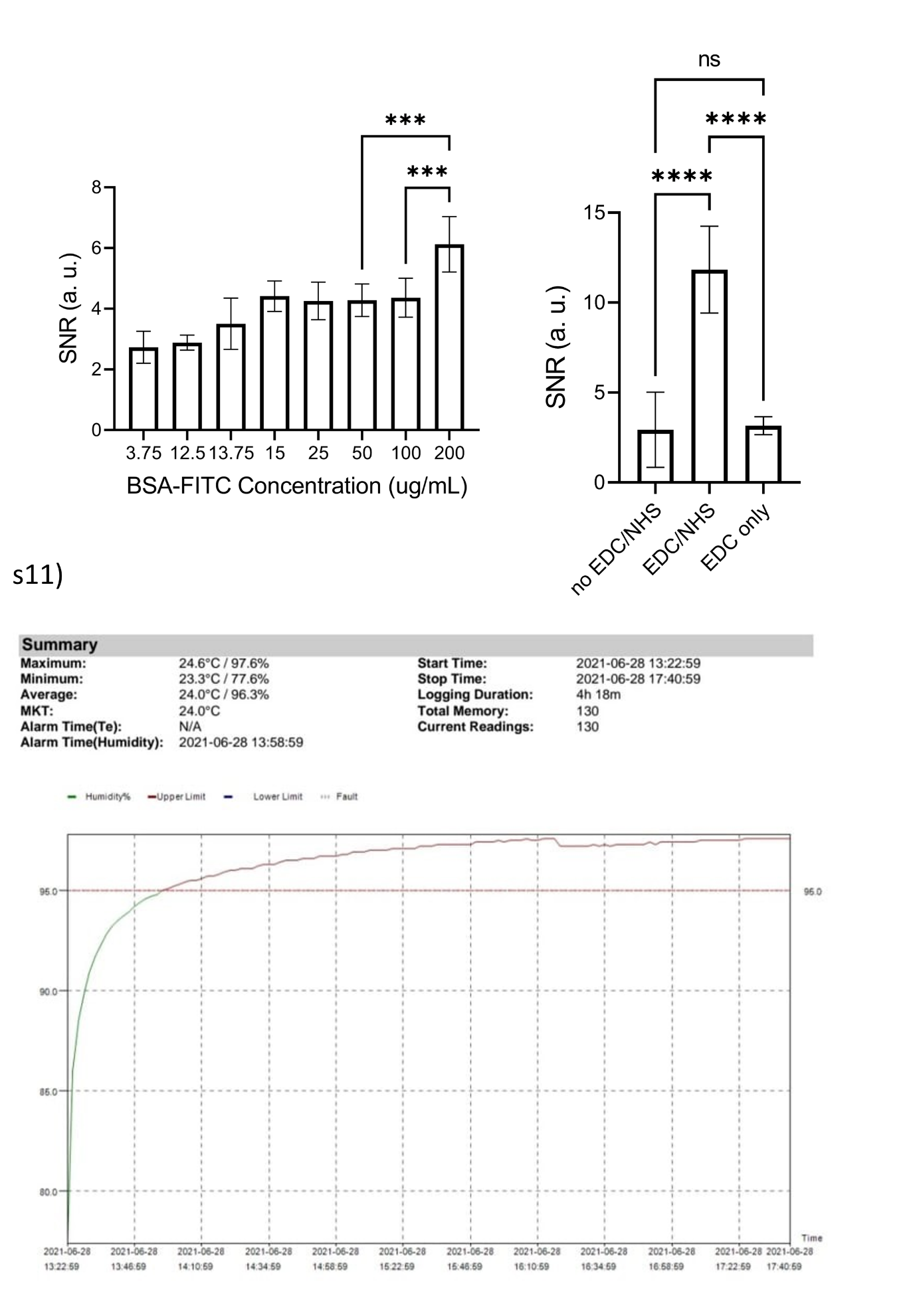


**Figure S19.** SNRs after µCP of BSA-FITC concentrations from 3.75 – 200 µg/mL (n = 12). Error bars calculated using the mean of standard deviation. Statistical analysis was conducted using one-way ANOVA followed by Tukey’s post hoc test, with nonsignificant (ns) statistical values where P = 0.5, and 1-star showing significance with P<0.1, 2-star with P<0.01, 3-star with P <0.001 and 4-star with P<0.0001.


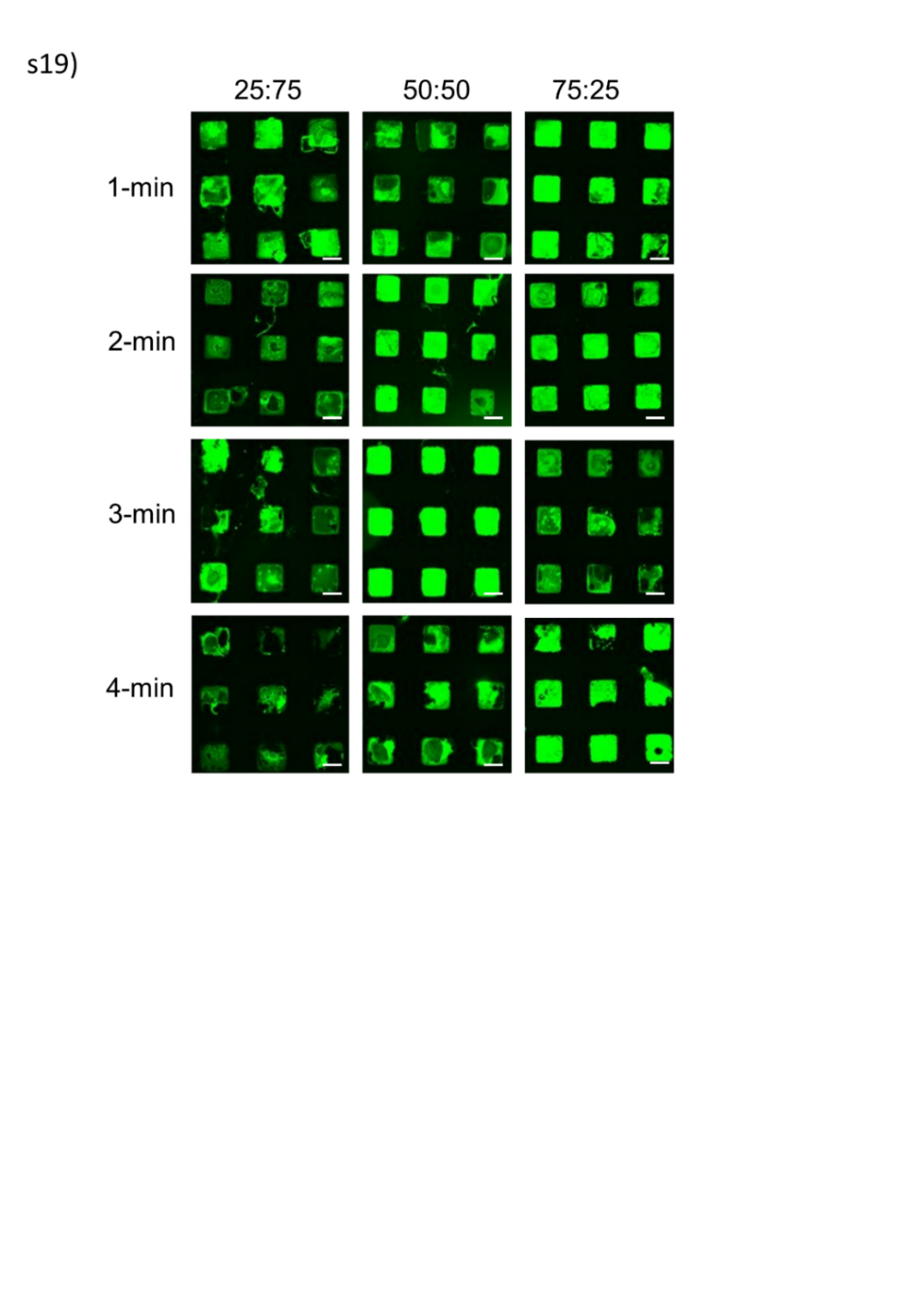


**Figure S20.** Fluorescence images, at 10X magnification, of printed substrates after µCP with bioinks containing EDC/NHS: BSA-FITC ratios of 25:75, 50:50, and 75:25, at dry times of 1-min, 2-min, 3-min and 4-min. Scale bars were 100 µm.


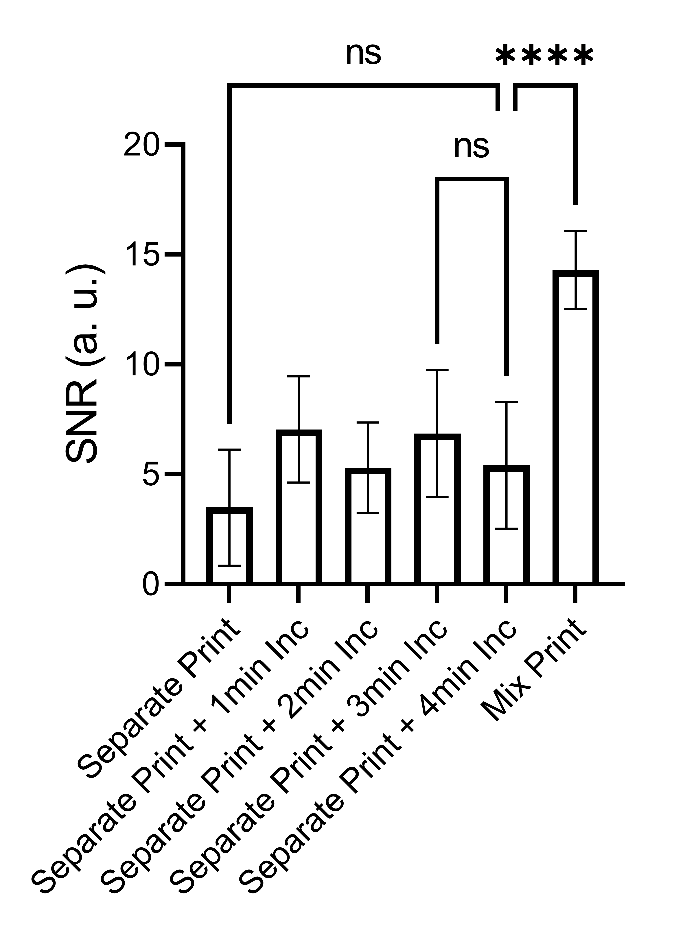


**Figure S21.** SNRs after µCP, where EDC/NHS and BSA-FITC were separately printed, where EDC/NHS diluted in glycerol and print solution were printed with a flat featureless stamp first, followed by a secondary print of BSA-FITC diluted in print solution, using featured stamps. Between these prints, an EDC/NHS incubation (Inc) was introduced, at 0-min, 1-min, 2-min, 3-min and 4-min. These separate prints were compared to mixed printing (n = 18). Error bars calculated using the mean of standard deviation. Statistical analysis was conducted using one-way ANOVA followed by Tukey’s post hoc test, with nonsignificant (ns) statistical values where P = 0.5, and 1-star showing significance with P<0.1, 2-star with P<0.01, 3-star with P <0.001 and 4-star with P<0.0001.


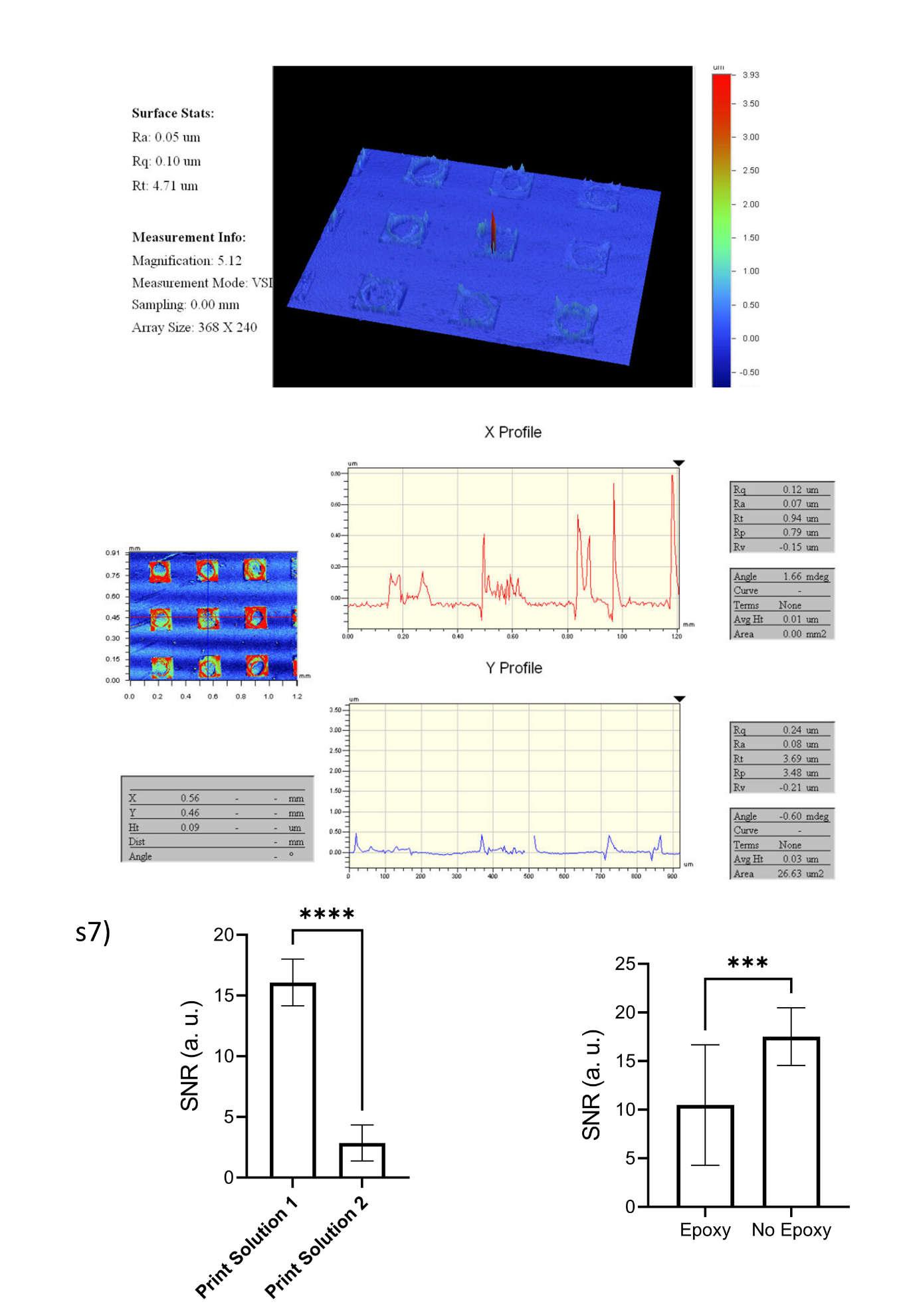


**Figure S22.** 3D projection of profilometry conducted on a BSA-FITC printed substrate (n = 9).


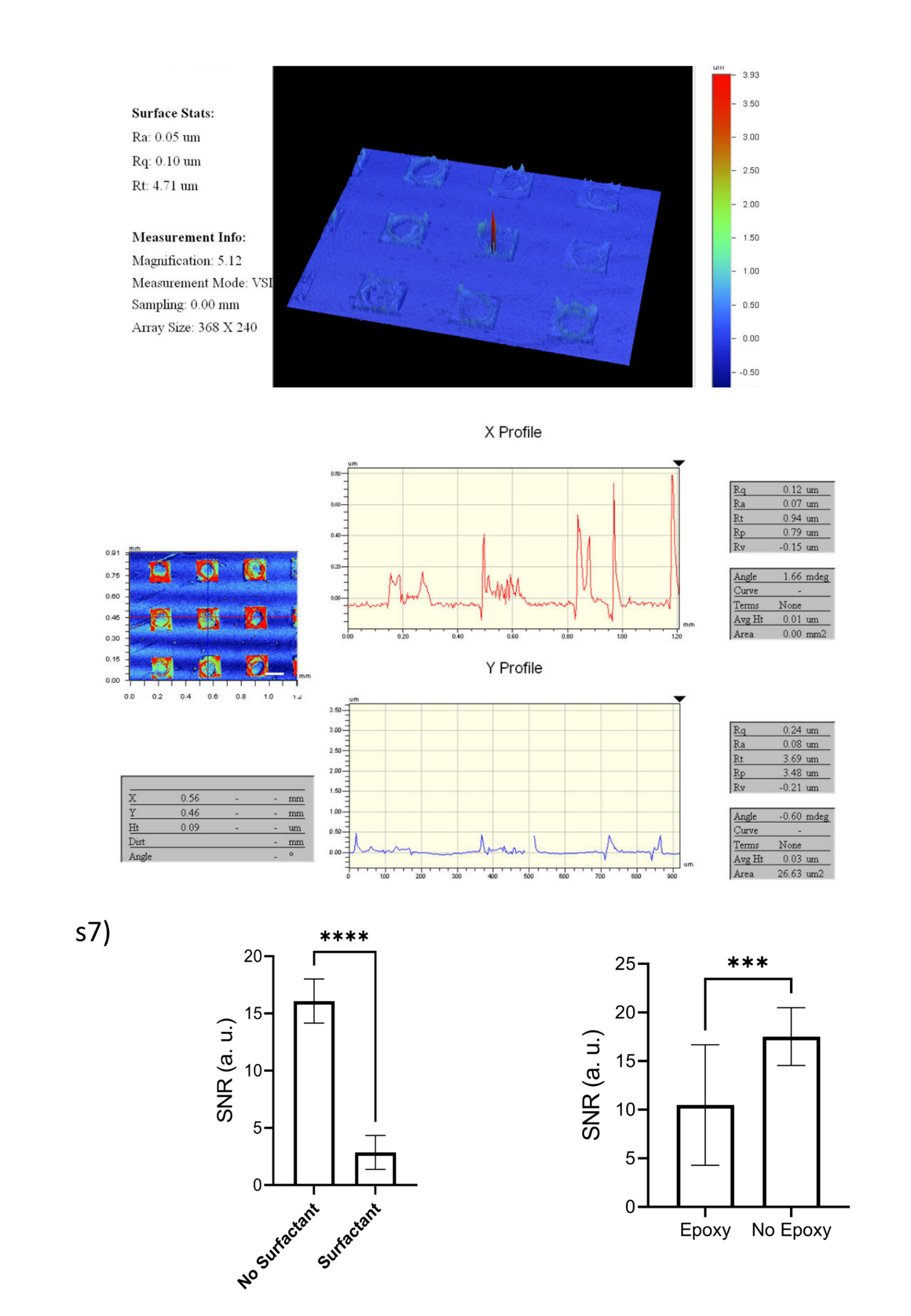


**Figure S23**. Sample X and Y profiles from the profilometry of the printed substrate, with specified parameters and roughness results.


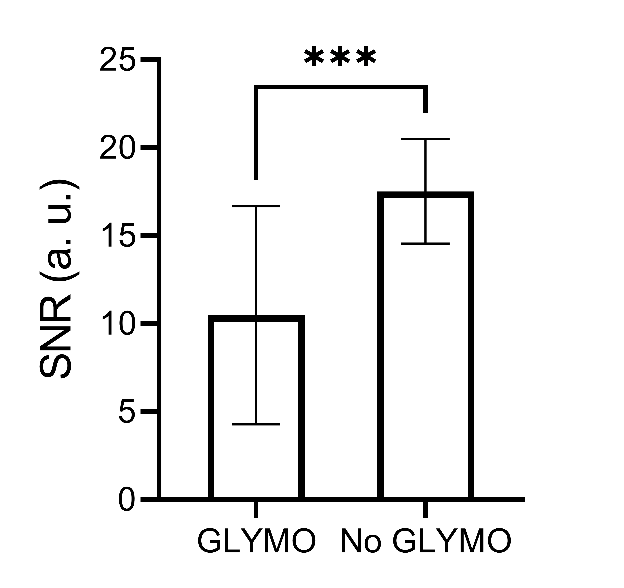


**Figure S24**. SNRs after µCP comparing bioink containing GLYMO to bioink without GLYMO (n = 18). Error bars calculated using the mean of standard deviation. Statistical analysis was conducted using Mann-Whitney U test, nonsignificant (ns) statistical values, P = 0.5, and 1-star showing significance with P<0.1, 2-star with P<0.01, 3-star with P <0.001 and 4-star with P<0.0001.


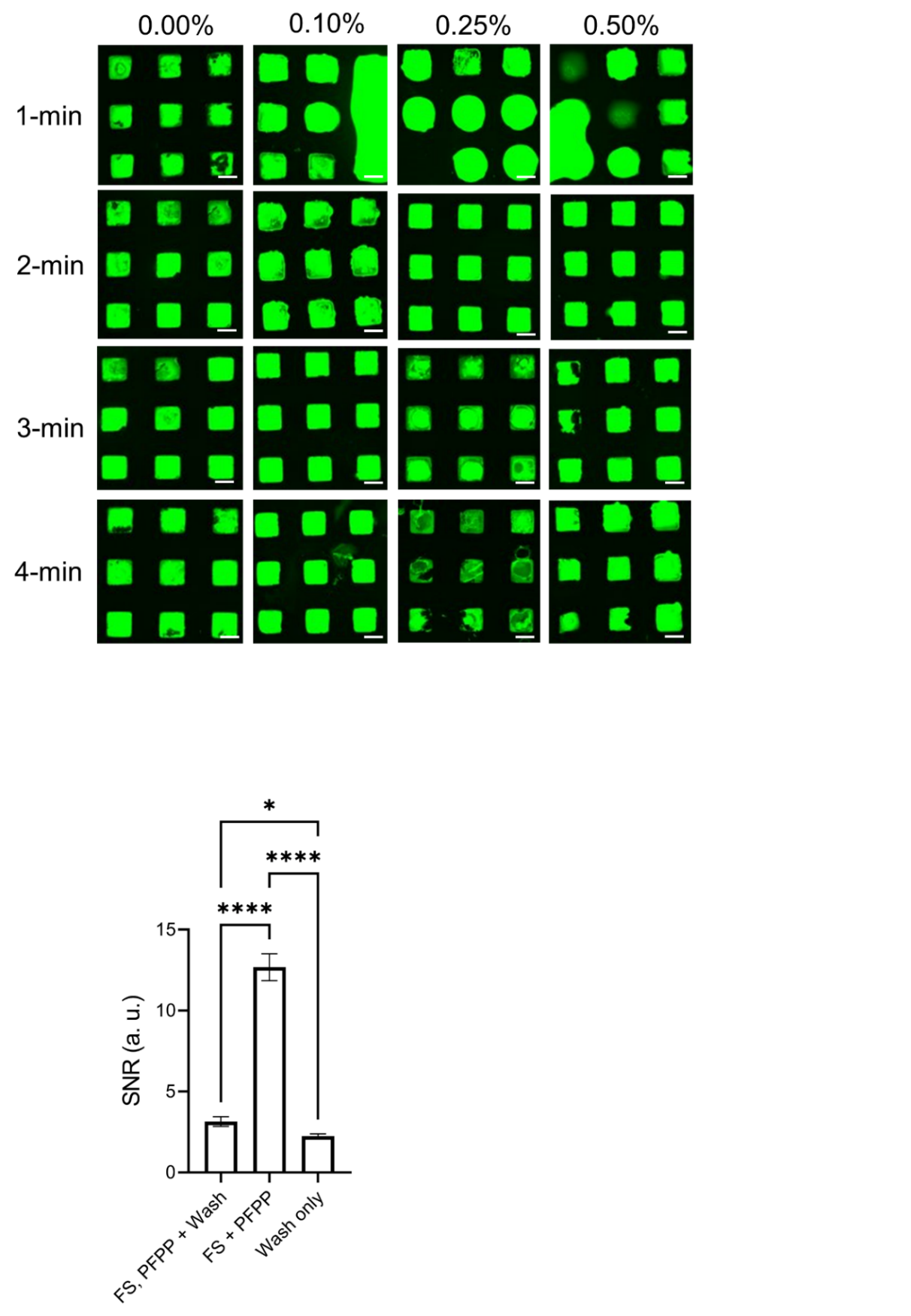


**Figure S25.** Fluorescence images, at 10X magnification, of printed substrates after µCP with bioinks containing glycerol concentrations of 0.00%, 0.10%, 0.25% and 0.50% at dry times of 1-min, 2-min, 3-min and 4-min. Scale bars were 100 µm.


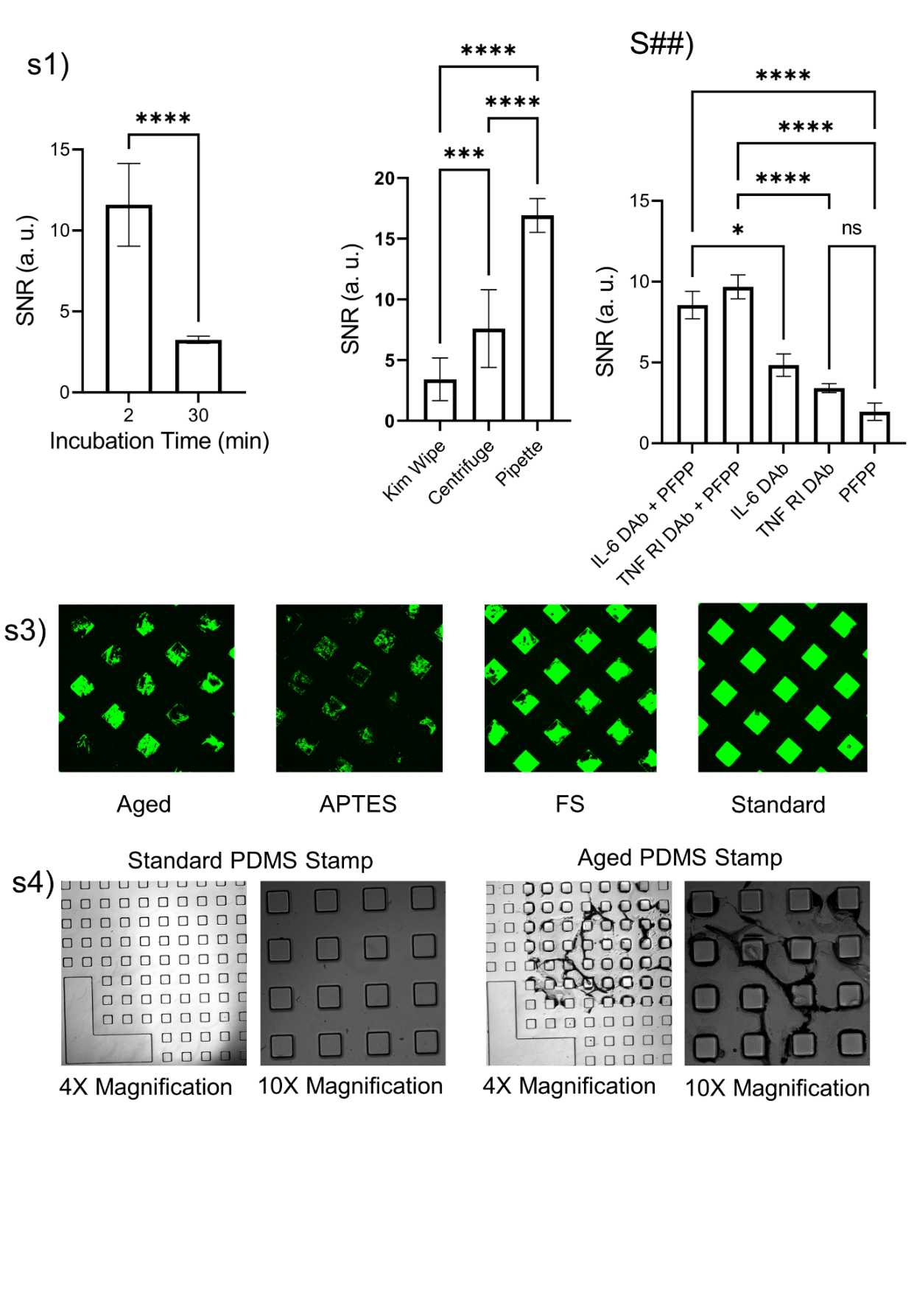


**Figure S26.** SNRs after µCP comparing Kimwipe, centrifuge and pipette droplet removal (n = 18). Error bars calculated using the mean of standard deviation. Statistical analysis was conducted using one-way ANOVA followed by Tukey’s post hoc test, with nonsignificant (ns) statistical values where P = 0.5, and 1-star showing significance with P<0.1, 2-star with P<0.01, 3-star with P <0.001 and 4-star with P<0.0001.


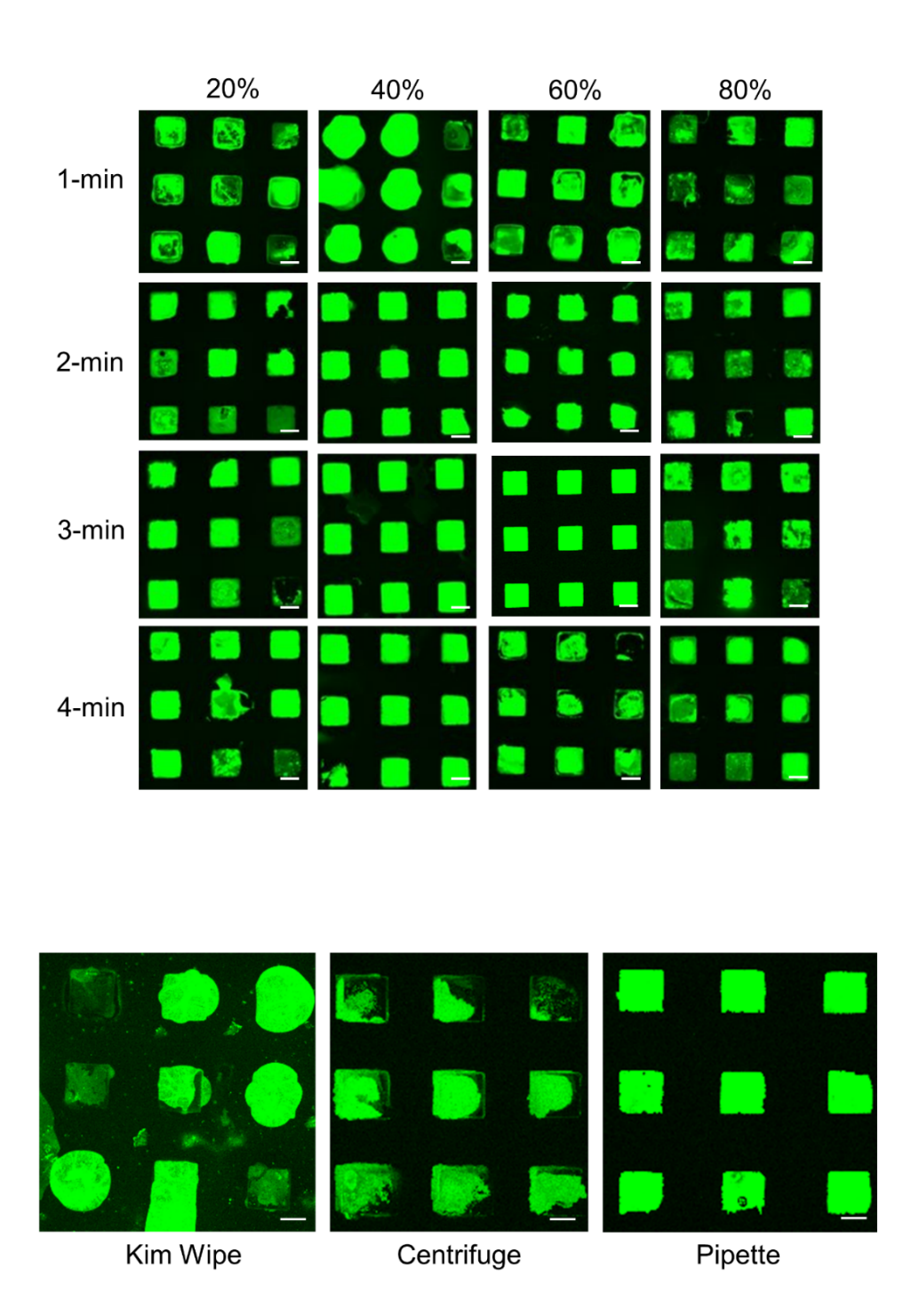


**Figure S27.** Fluorescence images, at 10X magnification, of printed substrates after µCP with Kimwipe, centrifuge and pipette droplet removal. Scale bars were 100 µm.


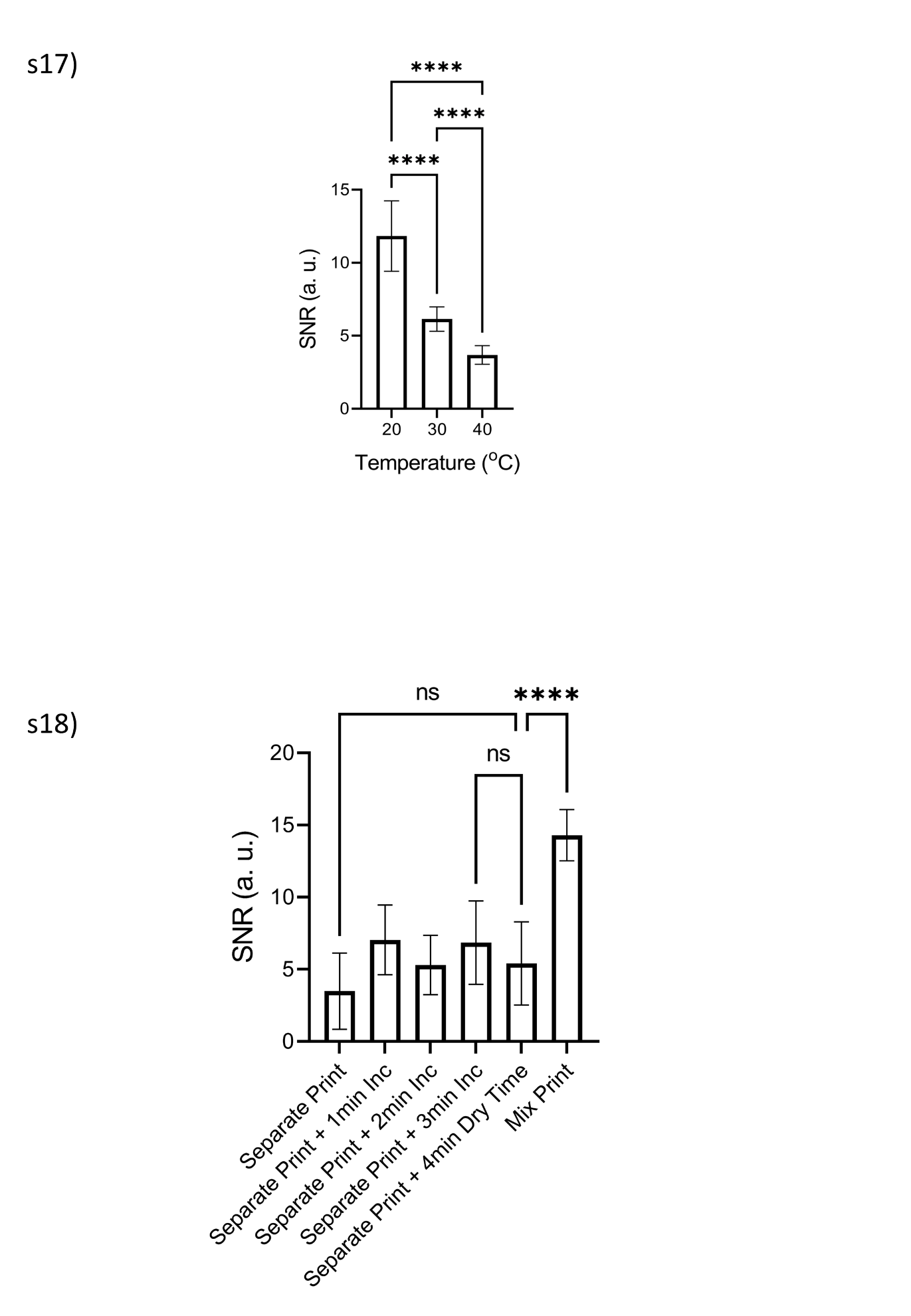


**Figure S28.** SNRs under 20℃, 30℃ and 40℃ environmental temperatures when printing (n = 18). Error bars calculated using the mean of standard deviation. Statistical analysis was conducted using one-way ANOVA followed by Tukey’s post hoc test, with nonsignificant (ns) statistical values where P = 0.5, and 1-star showing significance with P<0.1, 2-star with P<0.01, 3-star with P <0.001 and 4-star with P<0.0001.


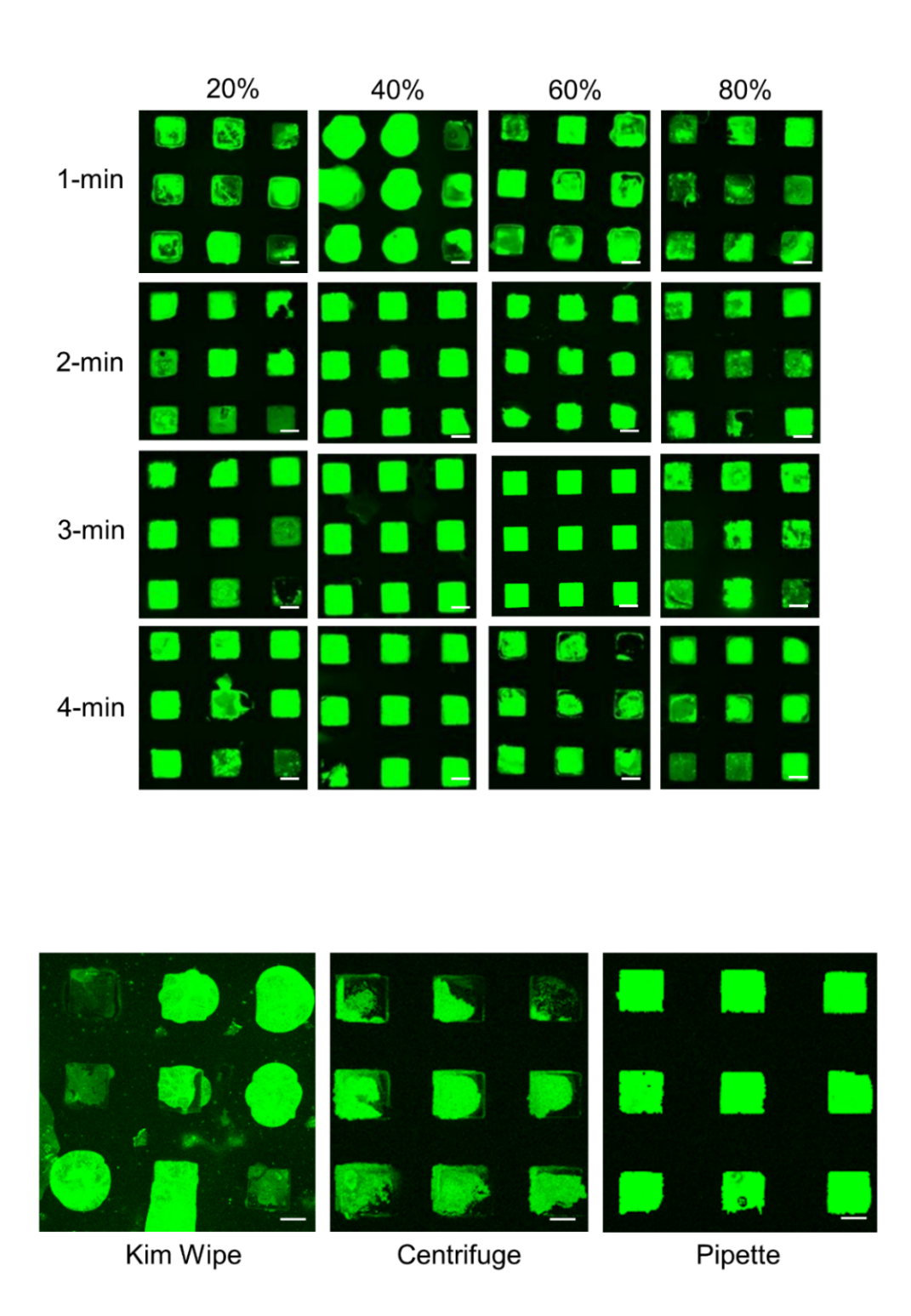


**Figure S29.** Fluorescence images, at 10X magnification, of substrates printed at 20%, 40%, 60% and 80% relative humidity, with 1-min, 2-min, 3-min, and 4-min dry times. Scale bars were 100 µm.


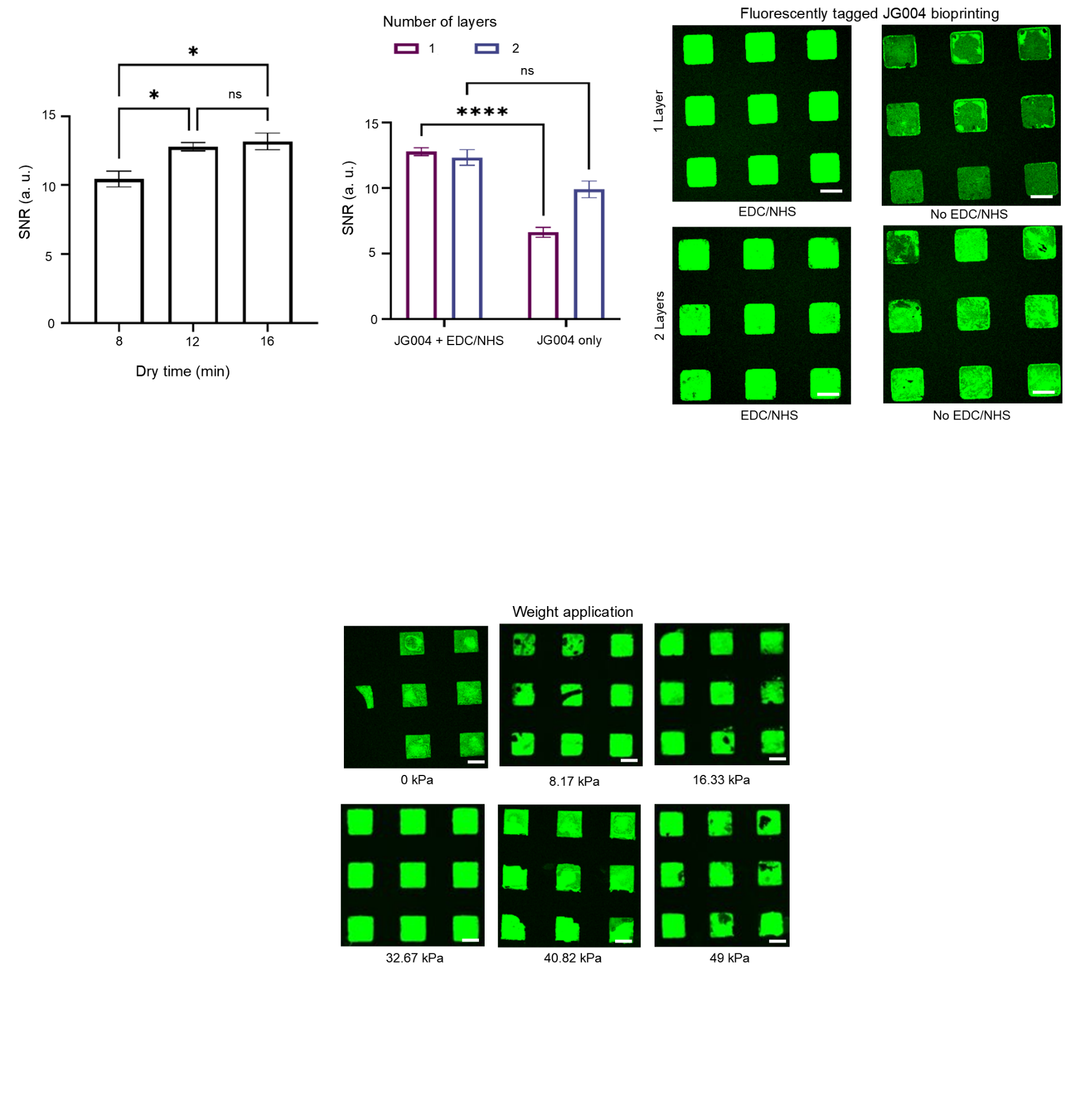


**Figure S30.** Fluorescence images after µCP stamps using applied forces of 0 kPa, 8.17 kPa, 16.33 kPa, 32.67 kPa, 40.82 kPa, 49 kPa, with optimized bioink and external environment conditions. Scale bars were 100μm.


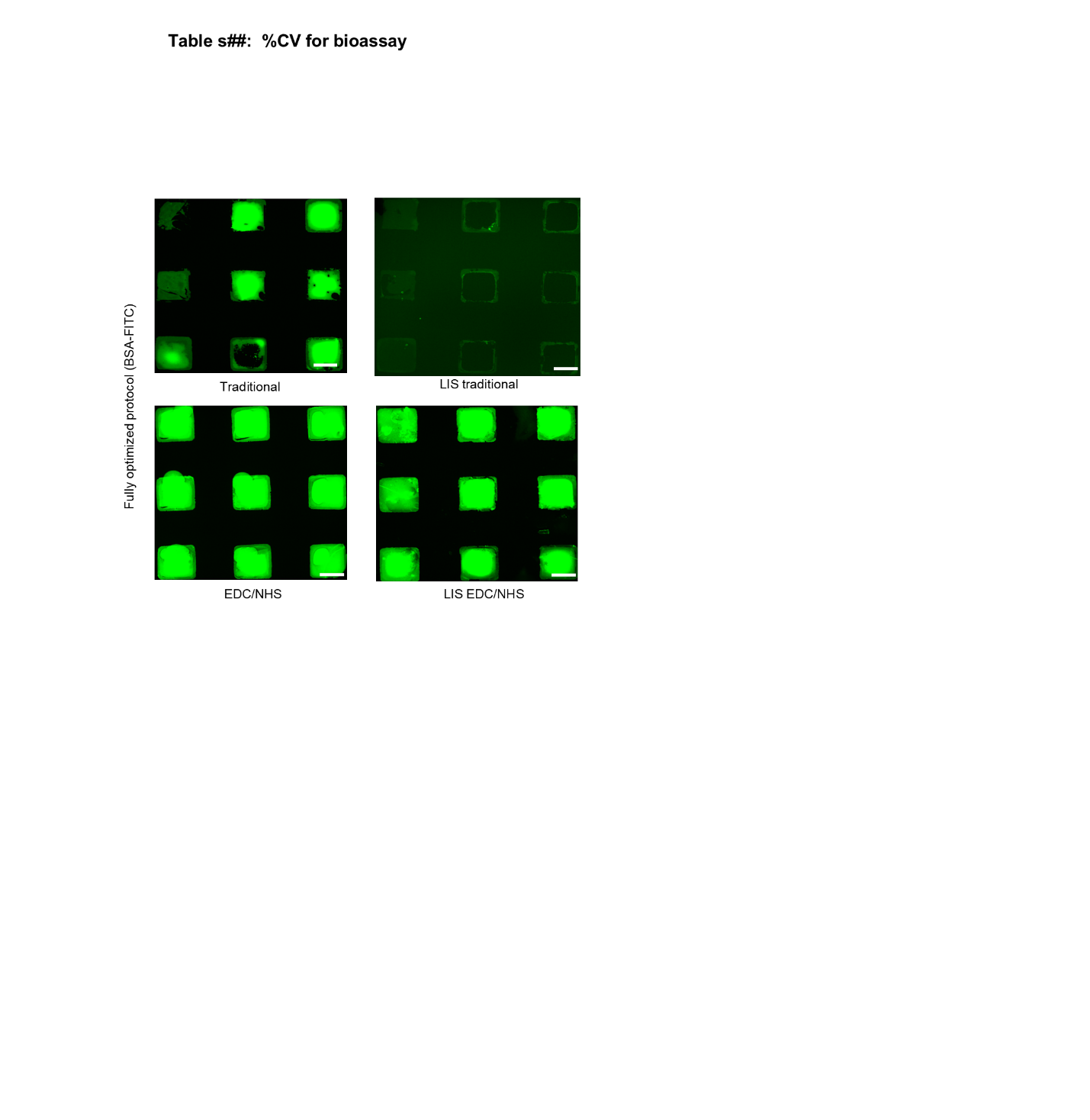


**Figure S31.** Fluorescence microscopy images of traditional bioprinting, utilizing O_2_ plasma and physical force application only, compared to our fully optimized protocol, utilizing CO_2_ and a combination of EDC/NHS with physical force application. Scale bars were 100μm.

**
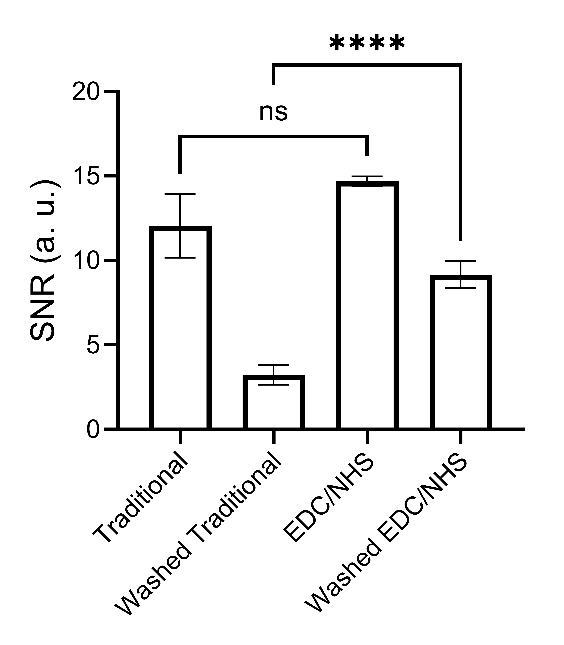
**

**Figure S32.** SNRs of traditional microarrays and dually immobilized microarrays with EDC/NHS, before and after wash three times with wash buffer solution (n = 9). Error bars calculated using standard error of the mean. Statistical analysis was conducted using one-way ANOVA followed by Tukey’s post hoc test, with nonsignificant (ns) statistical values where P = 0.5, and 1-star showing significance with P<0.1, 2-star with P<0.01, 3-star with P <0.001 and 4-star with P<0.0001.


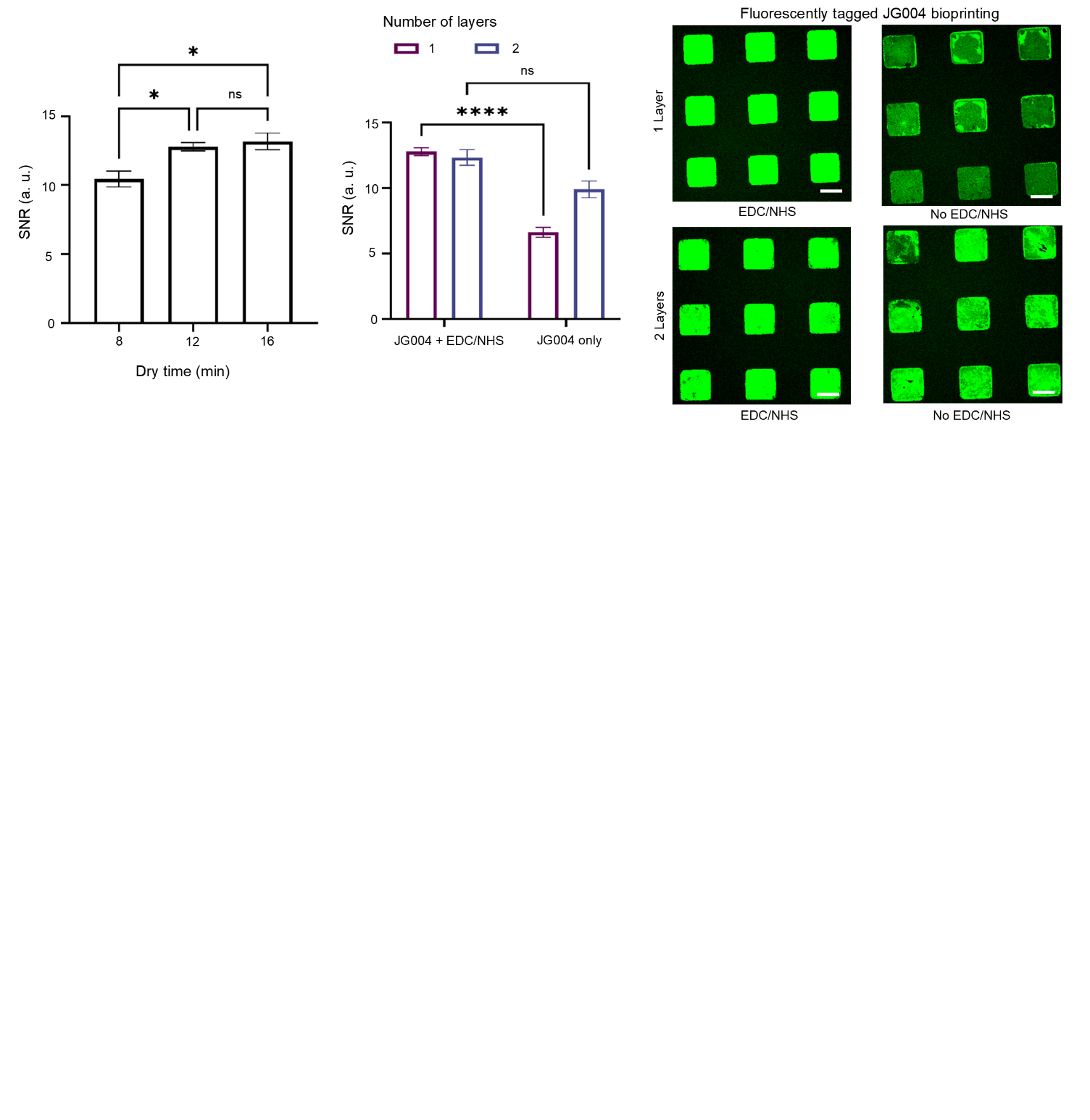


**Figure S33.** SNRs of SYBR-GOLD conjugated JG004 phage microarrays at dry times of 8-min, 12-min and 16-min (n = 9). Error bars calculated using standard error of the mean. Statistical analysis was conducted using one-way ANOVA followed by Tukey’s post hoc test, with nonsignificant (ns) statistical values where P = 0.5, and 1-star showing significance with P<0.1, 2-star with P<0.01, 3-star with P <0.001 and 4-star with P<0.0001.

**
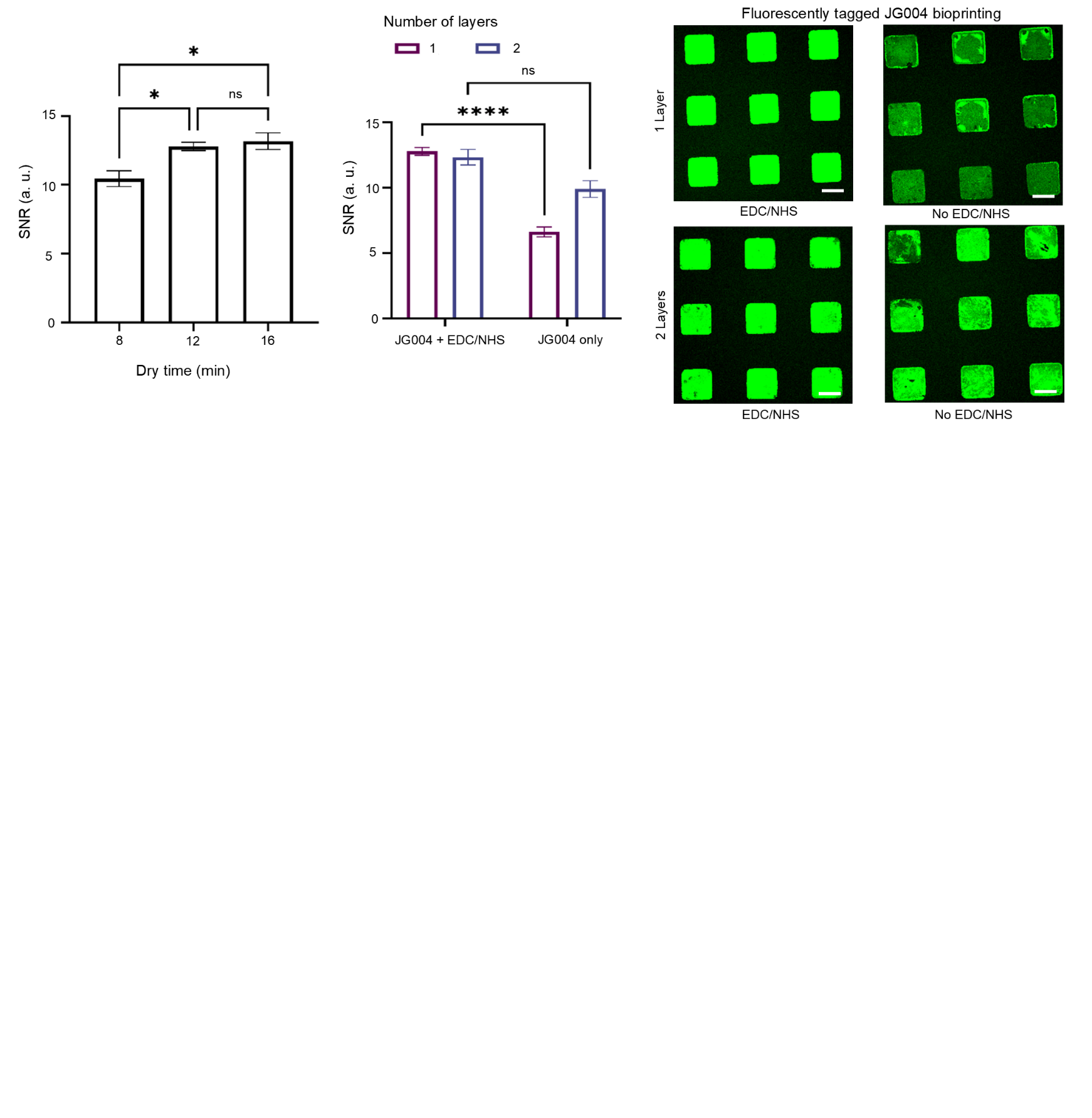
**

**Figure S34.** SNRs of μCP bacteriophage JG004 microarrays, with and without EDC/NHS, comparing 1-layer and 2-layer prints (n = 9). Error bars calculated using standard error of the mean. Statistical analysis was conducted using two-way ANOVA followed by Sadik’s post hoc test, where nonsignificant (ns) statistical values, P = 0.5, and 1-star showing significance with P<0.1, 2-star with P<0.01, 3-star with P <0.001 and 4-star with P<0.0001.

**
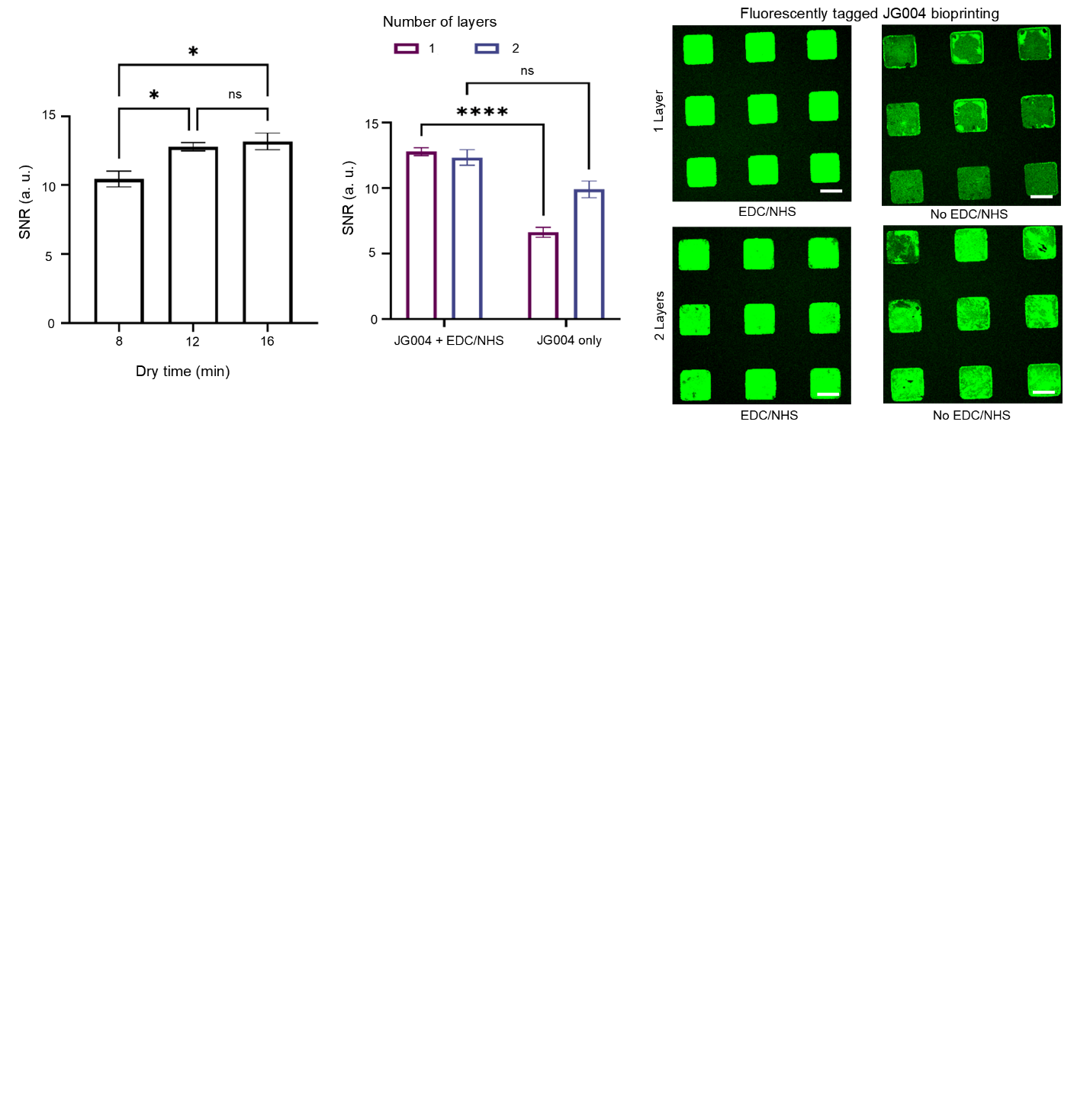
**

**Figure S35.** Fluorescence images of the 1-layer and 2-layer JG004 microarrays. Scale bars were 100 µm.

**Table S1.** **Peak area percentages (%) of chemical bonds from sample high resolution, deconvoluted XPS data of untreated plain, 15-min CO_2_ plasma treated and 15-min fluorosilanized PMMA substrates.**

| Binding energy (eV) | 284.69 | 286.05 | 288.52 | 290.78 | 293.11 |
| --- | --- | --- | --- | --- | --- |
| **Chemical Bond** | **C-C/C-H** | **C-O** | **O-C=O** | **-CF_2_** | **-CF_3_** |
| Peak area % | | | | | |
| Plain PMMA | 58.99 | 23.98 | 17.04 | 0.00 | 0.00 |
| CO_2_ Plasma Treated PMMA | 50.23 | 25.43 | 24.34 | 0.00 | 0.00 |
| FS Treated PMMA | 21.71 | 12.08 | 6.96 | 49.50 | 9.74 |

**Table S2. Comparison of reported µCP protocols in terms of versatility, resolution, cost, and time.**

| **Title** | **Fabrication method** | **Bioinks** | **Features** | **Cost** | **Print Time** | **Versatility** | **Applications** | **Ref** |
| --- | --- | --- | --- | --- | --- | --- | --- | --- |
| Covalent automated microcontact bioprinting of lubricant-infused microarrays for scalable manufacturing | Bioinks containing biorecognition agents, EDC/NHS and glycerol are applied and incubated onto PDMS stamps in 65% humidity, followed by removal, air drying and weight application. | BSA-FITC, monoclonal antibodies, fluorescent and non fluorescent lytic bacteriophage | Squares at a resolution of 150 μm | Low | 6min – 20min | High | High throughput bacterial bioassays ready for industry scale translation | ^N/A^ |
| Facile and rapid microcontact printing of additive-free polydimethylsiloxane for biological patterning diversity | Stamping of spin-coated uncured PDMS bioink, followed by heat curing, to create PDMS negative mold of multiple patterns for cell culturing and microtissue reconstruction | Uncured PDMS | Circles, squares, and hexagons at resolution of 200–400 μm | Low | 8min | High | Droplet microfluidics, tissue reconstruction, 2D and 3D cell culturing | ^[61]^ |
| Combined laser-induced graphene and microcontact printing for processing scalable and stackable micro-stripe patterns toward multifunctional electronic devices | O_2_ plasma treated PDMS stamps spin coated with polyamic acid are printed multiple times to fabricate stacked features, which are then laser treated to form laser-induced graphene (LIG) | Polyamic acid | Lines and squares at resolution of 4.83 - 22.32 μm | Low | 4.5 hours | Low | Miniaturized thin-film graphene electronics and liquid sensors | ^[62]^ |
| Synthesis and microcontact printing of novel nanoflowers of ZnO_Ag_rGO nanocomposite to check microbial adhesion | Application of fluorescently tagged nanocomposite bioinks on glass slide, followed by PDMS stamp inking and application on silanized substrates | Binary Zinc Oxide_Reduced Graphene Oxide (ZnO_rGO); ternary Zinc Oxide_Silver_Reduced Graphene Oxide (ZnO_Ag_rGO) | 3D nanoflowers at size resolution of 70–80 nm | High | 24 hours | Low | Surface modification using to create hierarchy and reduce biofilm formation as coatings for medical devices | ^[63]^ |
| Defined transfer of colloidal particles by electrochemical microcontact printing | Gold-treated PDMS stamps are immersed in electrolyte solution, followed by application of tunable electrochemical potential for bioink transfer | Colloidal nanoparticle suspensions (180nm diameter particles), DNA and polymers containing potential differential | Squares at resolution of 5μm | High | 10 seconds | High | Mesostructured or plasmonic structured fabrication | ^[64]^ |
| Vapor-phase chemical etching of silicon assisted by graphene oxide for microfabrication and microcontact printing | Graphene oxide spin coated onto cycloolefin polymer stamps and stamped onto silicon substrate, followed by vapor deposition in vacuum oven with etching solution | Graphen oxide with etching solution | Circles at resolution of 5μm | Moderate | 24 hours | Low | Silicon etching for nanofabrication of electronics | ^[65]^ |
| Shaping metallic nanolattices: design by microcontact printing from wrinkled stamps | Nanolattice printing using wrinkled PDMS stamps in a two-step printing approach: 1) printing of oligomer PDMS with poly(2-vinylpyridine-*co*-2-hydroxyethyl acrylate) as backfiller; 2) printing of poly(ethylene imine) and functional nanoparticles | Functional nanoparticle and lattices (e.g. gold nanoparticles stabilized using citrate) | Nanolattice stripes at resolution of 0.8 – 3.3 µm wavelength | Moderate | 32 hours | Moderate | Alternative technique to lithography for nanoparticle printing | ^[66]^ |
| Polymer brush-assisted microcontact printing: using a tailor-made polydimethylsiloxane (PDMS) stamp for precise patterning of rough surfaces | PDMS stamps are decorated with brushes *via* photoiniferter reversible addition and fragmentation chain transfer (PI-RAFT) polymerization and printing of APTES containing bioinks | APTES and alkyl silane containing solutions | Lines at a resolution of 4 µm | Moderate | 16hr | Moderate | Bioprinting with brush-decorated PDMS stamps for patterning capillary-active oxide surfaces | ^[67]^ |
| Beta-amyloid enhances vessel formation in organotypic brain slices connected to microcontact prints | Bioinks are spread along PDMS stamp with coverslip, incubated for 15min, followed by transfer onto semipermeable membranes | Beta-amyloid, tau, antibodies and collagen hydrogel bioinks diluted in PBS | Lines at a resolution of 75 µm | Low | 15min | Low | Assessing the effects of printed beta-amyloid in mouse brain tissue samples in Alzheimer’s Disease | ^[68]^ |
| Microcontact printing of biomolecules on various polymeric substrates: limitations and applicability for fluorescence microscopy and subcellular micropatterning assays | 11 different polymer substrates are functionalized and printed on using PDMS stamps | BSA-Cy5, fluorescent antibodies and cytosolic proteins | Squares at a resolution of 3 µm | Low | 1.5hr | High | Creating micropatterns on membrane-like structures for live cell experiments and total internal reflection fluorescence microscopy | ^[69]^ |
| High-resolution continuous microcontact printing on various substrates for organic and oxide electronics | Roll-to-roll device containing circular PDMS stamp onto various polymer substrates | Fluorinated polymer solutions | Lines at a resolution of 10 µm | High | 13min | High | Scaled up electronic and circuit printing | ^[70]^ |
| Sub-micron patterning of metal oxide surfaces via microcontact printing and microtransfer molding of amphiphilic molecules and antifouling application | Use of nanostructured PDMS stamps, fabricated by DVD and CD optical media manufacturing techniques to transfer patterns of alkyl silanes onto silicone, mica and aluminum using | n-octadecylphosphonic acid and n-octadecyltriethoxysilane bioinks | Lines at a resolution of 2 – 150nm | Moderate | 10min | Moderate | Inducing decreased bacterial adhesion for hydrophobic antimicrobial surface fabrication | ^[71]^ |
| Experimental study on flow boiling heat transfer performance of nanowire-printed substrates with porous-like structures | Flat PDMS stamps were used to repeatedly transfer nanoparticles onto substrates under controlled humidity, temperature and gas pressures | Zinc oxide nanoparticles | 3D wires at a resolution of 2.6 - 12 µm | High | 5 – 30hr (depending on length) | Moderate | Fabrication of nanowires for heat transfer engineering applications | ^[72]^ |

**Table S3. Failure Mode and Effects Analysis (FMEA) showing severity, frequency of occurrence, detectability and risk priority number.**

| **Process step** | **Failure mode** | **Failure effect** | **SEV** | **Potential causes** | | **OCC** | **Current process controls** | **DET** | **RPN** |
| --- | --- | --- | --- | --- | --- | --- | --- | --- | --- |
| ***1. Stamp fabrication*** | | | | | | | | | |
| Mixing curing agent and elastomer at 1:10 (V:V) | Incomplete solidification of PDMS stamps (tacky stamps) | Getting PDMS residue on the patterned areas after µCP that could lead to false fluorescence signals | 8 | | Insufficient mixing  Inconsistent measurement of curing agent and elastomer | 1 | Conducting test run of stamping without BSA-FITC. If fluorescence is detected, this indicates PDMS residue. | 1 | 8 |
| Degassing in desiccator | Trapping bubbles inside the stamp | Imprecise patterns (rounded edges and blobs) | 7 | | Insufficient time for degassing | 1 | Visual observation to ensure no air bubbles | 1 | 7 |
| Pouring into the silicon mold | Trapping bubbles inside the stamp | Imprecise patterns (rounded edges and blobs in the pattern) | 8 | | Pouring the PDMS too quickly | 2 | Visual observation of the PDMS after degassing to ensure there are no air bubbles | 1 | 16 |
| Curing the PDMS at 150 °C for 15 min | Incomplete solidification of PDMS stamps (tacky stamps) | Getting PDMS residue on the patterned areas after µCP that could lead to false fluorescence signals | 8 | | Having high volume of PDMS in the mold (uneven distribution of temperature throughout the PDMS) | 1 | Conducting a test run of stamping without BSA-FITC. If fluorescence is detected under the microscope, this is an indicator of PDMS residue. | 1 | 8 |
| Removing the PDMS stamp and cutting it to the desired size | Damage to the patterned surface or stamp mold while cutting the stamp | Imprecise patterns (incomplete or cut off patterns) | 8 | | Having a very high volume of PDMS in mold (results in solidified PDMS that is too thick to cut cleanly) | 1 | Observation under a microscope of the PDMS after cutting out the stamp to ensure there is cut-off or incomplete patterns | 1 | 8 |
| Sonication of stamps for 5 min | Erosion of stamp | Losing the precision of the patterns over time after each sonication | 2 | | Leaving stamps in ethanol for too long; repeated sonication eventually decreases pattern quality | 2 | Conducting a weekly test run of stamping procedure with BSA-FITC to check the quality of the patterns. | 1 | 4 |
| ***2. µCP stamping*** | | | | | | | | | |
| Plasma treatment of substrates with CO_2_ | Insufficient hydrophilic properties of substrate for stamping | Getting faded patterns because of hydrophobic recovery of the substrate and non-covalent attachment of BSA-FITC | 7 | | Insufficient plasma duration; waiting more than 30min after plasma treatment to stamp on substrate | 5 | Measuring hydrophilic properties using contact angle | 2 | 70 |
| Preparation of bioink containing BSA-FITC, glycerol, EDC/NHS | Aggregation of BSA-FITC because of self-crosslinking *via* EDC/NHS chemistry | Low yield of immobilized BSA-FITC after µCP on the surface; inconsistency of the patterns | 5 | | Time sensitivity of EDC/NHS reactions; inconsistent concentration and ratios of reagents | 4 | µCP BSA-FITC and checking the stability and fluorescence intensity | 7 | 140 |
| Adding bioink as sessile drops onto featured PDMS stamps and leaving on for 2 min | Inaccurate placement of droplets | Inconsistent number of replicates measurable, reducing fluorescent signal sensitivity | 2 | | Droplet application not standardized and inconsistent | 2 | Observing the stamp to ensure the droplets are in the right spots. | 1 | 4 |
| Removing the droplets with pipettes | Difficulty removing the whole droplet which results in residue print solution on stamp | Imprecise patterns in a square shape (blobs or rounded edges because of the stamp being too wet) | 8 | | Inconsistent dry time so bioink evaporates; waiting too long between removals for multiple droplets; droplet removal not standardized and inconsistent | 5 | Observing the stamp to ensure the stamp is sufficiently dry; observing the printed substrate to ensure the surface is not wet after µCP | 7 | 280 |
| Air drying the stamp | The print solution drying out on the stamp; the print solution remaining slightly wet on the stamp after drying | Getting faded or inconsistent patterns because of the print solution drying out; getting blobs or rounded edges because of the print solution remaining wet | 8 | | Waiting too long between removal of multiple droplets (first droplet will have dried for longer); droplet removal not standardized and inconsistent; inconsistent humidity, temperature and environmental conditions during air drying process. | 7 | Observing the stamp to ensure the stamp is sufficiently dry; observing the printed substrate to ensure the surface is not wet after µCP | 7 | 392 |
| Placing stamp onto the plasma treated substrate | Movement of the stamp during placement | Imprecise patterns (double square patterns or extra outlines around pattern) | 5 | | Shifting the stamp position during the placement process | 1 | µCP BSA-FITC and checking the uniformity of the patterns | 1 | 5 |
| Force application on the stamp for 1min | Movement of the stamp during weight placement; inconsistency in force distribution due to inaccurate weight placement | Imprecise patterns (double square patterns or extra square outlines surrounding the pattern) | 8 | | Weight is off centered, with uneven distribution; insufficient force applied; placement not standardized and inconsistent. | 8 | µCP BSA-FITC and checking the uniformity of the patterns. | 2 | 128 |
| Removing the weight and the stamp | Movement of the stamp during removal of the weight on top | Imprecise patterns (double square patterns or extra square outlines surrounding the pattern) | 5 | | Weight sliding against stamp during removal; Stamp getting stuck to the weight | 1 | µCP BSA-FITC and checking the uniformity of the patterns. | 1 | 5 |
| ***3. Lubricant infusion*** | | | | | | | | | |
| Incubation of the patterned substrate in controlled humidity under dark conditions for 1hr | Changes in environmental conditions during incubation; non-covalent attachment of BSA-FITC due to the lack of humidity | Photobleaching of the pattern or getting faded/inconsistent patterns because of inefficient incubation | 4 | | Leaving printed substrate in an environment that is too bright or has insufficient humidity | 1 | Measuring the humidity level during the incubation; checking the stability of the printed areas by performing washing steps on a shaker | 1 | 4 |
| Placing the substrate in the vacuumed desiccator with 200μL FS | Incomplete FS CVD procedure | Smearing of the pattern because of washing without proper FS CVD | 3 | | Inconsistent vacuum and pressure during FS CVD; high levels of humidity can rapidly hydrolyze the FS (e.g. during the summer) | 1 | Measuring contact angles after FS CVD | 1 | 3 |
| Assembling the superstructure | Misplacement of superstructure on substrate | Smearing of the pattern because of incomplete washing of entire pattern (the entire pattern might not be aligned to the wells of superstructure) | 1 | | Placement of droplet at superstructure well boundaries might result in portions of the pattern getting cut off; misalignment of superstructure wells to substrate; improperly securing the substrate to the superstructure, resulting in leakage | 1 | Visual observation of the patterns after superstructure alignment | 2 | 2 |
| Lubrication of the surface with 50 μl/well of PFPP | Inaccurate distribution of PFPP in well | Getting concentrated high levels of background noise in certain areas | 1 | | Insufficient amount of lubricant trapped on entire surface (not uniformly spread) | 1 | Visual observation to ensure wells are covered | 1 | 1 |
| Removing the excess PFPP and washing each | Insufficient washing Insufficient amount of lubricant | Smearing of the pattern or inconsistent patterns because of incomplete washing of entire pattern; high background noise | 1 | | Residue lubricant might remain after washing; lubricant evaporates if the surface is not immediately covered | 1 | Visual observation of the lubricant residue | 1 | 1 |
| ***4. Imaging*** | | | | | | | | | |
| Imaging with 300ms exposure using FITC channel at 10X | Placement of sample under microscope and set up of imaging | Fuzzy patterns while imaging | 2 | | Removing superstructure increases risk of touching the patterns while imaging; risk of photobleaching if not imaged quickly | 5 | Checking the focuses and resolution | 1 | 10 |

**The FMEA Process and Application in μCP**

The first step of the FMEA was breaking down the μCP protocol into a step-by-step process. These steps were categorized based on overall function, as follows: 1) the casting and creation of the PDMS stamps with square feature patterns (stamp fabrication); 2) the physical transfer and covalent immobilization of the bioink onto functionalized PMMA substrates (μCP stamping); 3) performing fluorosilanization and creating the lubricant infused surfaces (lubricant infusion); and 4) conducting the fluorescence imaging of the final microarrays (imaging). From here, a list of failure modes, defined as the specific failure method that prevents each step from achieving its function, was generated. Additionally, their failure effects on the final microarrays were determined for each step’s failure mode. From the failure effects, their severity (SEV) on the final microarray quality and uniformity was assigned, from a scale of 1 to 10, with 1 being the least severe and 10 being the most severe. Potential causes of each failure were detailed, highlighting methods in which these causes were detected, which pinpointed areas that optimization may occur. The frequency of failure mode’s occurrence (OCC) was defined by analyzing the causes, from a scale of 1 to 10, with 1 being the least frequent and 10 being the most frequent. Next, the current process controls for detecting and limiting the failure modes were outlined for each step. The overall failure mode detectability (DET), or ease of detection, was selected from a scale of 1 to 10, with 1 being easiest to detect and 10 being the most difficult to detect. Finally, the risk priority number (RPN) was calculated by taking the product of the severity, frequency of occurrence, and detectability (RPN = SEV*OCC*DET) for each step, and the steps with the highest RPN were determined as high risk and required optimization. The five highest risk steps were as follows: plasma treatment of substrates with CO_2_ (RPN = 70); preparation of bioink containing BSA-FITC, glycerol, EDC/NHS (RPN = 140); removing the droplets with pipettes (RPN = 280); air drying the stamp (RPN = 392); and force application on the stamp for 1min (RPN = 128). Note, all the high-risk steps are part of the stamping process, while low risk steps are from stamp fabrication, lubricant infusion and imaging processes. The reason is that the stamping process is highly time sensitive and prone to changes in external environmental factors, such as humidity and temperature. The stamping process is where the immobilization of biorecognition agents takes place, so if any failures were to occur at this stage, immobilization is negatively affected, and the final microarrays’ quality is reduced. In comparison, stamp fabrication occurs before the immobilization with many controls already set in place. PDMS casting is also not a time sensitive or reactive process. Lubricant infusion and imaging are also done post-processing, with much fewer steps involved overall. Thus, all these steps are high severity and can occur more frequently during stamping. Also, bioink preparation, droplet removal, and air drying are difficult to detect, as the failure occurrence is mainly visually detected and only observed after the step is complete. Meanwhile CO_2_ plasma is easier to detect through methods such as contact angle measurements between prints. For weight application, a force sensor can be implemented in the system to ensure sufficient force is applied for physical transfer of biorecognition agents. These analyses thus informed the optimization of the μCP protocol to focus on the time sensitive bioinks and external factors while stamping, as well as provided insight on where automation can be most reliable.

**Table S4. Intra-assay and inter-assay %CVs of EDC/NHS: BSA-FITC bioinks at various dry times, over two wells with 9 replicants each well.**

| **BSA-FTC: EDC/NHS** | **Dry time [min]** | **Well** | **Intra-assay CV [%]** | **Inter-assay CV [%]** |
| --- | --- | --- | --- | --- |
| 25:75 | 1 | 1 | 27.17 | 28.38 |
|  |  | 2 | 29.58 |  |
| 25:75 | 2 | 1 | 47.82 | 34.13 |
|  |  | 2 | 20.44 |  |
| 25:75 | 3 | 1 | 43.55 | 33.05 |
|  |  | 2 | 22.45 |  |
| 25:75 | 4 | 1 | 26.40 | --- |
|  |  | 2 | --- |  |
| 50:50 | 1 | 1 | 19.99 | 14.74 |
|  |  | 2 | 9.49 |  |
| 50:50 | 2 | 1 | 6.06 | 14.24 |
|  |  | 2 | 22.43 |  |
| 50:50 | 3 | 1 | 17.70 | 12.59 |
|  |  | 2 | 5.12 |  |
| 50:50 | 4 | 1 | 13.82 | 15.89 |
|  |  | 2 | 17.95 |  |
| 75:25 | 1 | 1 | 14.19 | 11.42 |
|  |  | 2 | 8.66 |  |
| 75:25 | 2 | 1 | 26.11 | 16.26 |
|  |  | 2 | 6.42 |  |
| 75:25 | 3 | 1 | 15.91 | 23.74 |
|  |  | 2 | 31.57 |  |
| 75:25 | 4 | 1 | 5.04 | 18.59 |
|  |  | 2 | 32.13 |  |

**Table S5. Intra-assay and inter-assay %CVs of glycerol concentrations (%) within bioinks at various dry times, over two wells with 9 replicants each well.**

| **Glycerol [%]** | **Dry time [min]** | **Well** | **Intra-assay CV [%]** | **Inter-assay CV [%]** |
| --- | --- | --- | --- | --- |
| 0% | 1 | 1 | 12.41 | 17.05 |
|  |  | 2 | 21.69 |  |
| 0% | 2 | 1 | 19.69 | 21.21 |
|  |  | 2 | 22.72 |  |
| 0% | 3 | 1 | 13.26 | 11.46 |
|  |  | 2 | 9.66 |  |
| 0% | 4 | 1 | 5.39 | 6.39 |
|  |  | 2 | 11.90 |  |
| 0% | 5 | 1 | 16.64 | 11.19 |
|  |  | 2 | 5.73 |  |
| 0.1% | 1 | 1 | --- | --- |
|  |  | 2 | 26.15 |  |
| 0.1% | 2 | 1 | 7.04 | 19.97 |
|  |  | 2 | 25.89 |  |
| 0.1% | 3 | 1 | 8.81 | 11.19 |
|  |  | 2 | 13.58 |  |
| 0.1% | 4 | 1 | 15.17 | 19.55 |
|  |  | 2 | 21.28 |  |
| 0.1% | 5 | 1 | 8.61 | 21.37 |
|  |  | 2 | 24.48 |  |
| 0.25% | 1 | 1 | 14.25 | 16.21 |
|  |  | 2 | 10.43 |  |
| 0.25% | 2 | 1 | 9.07 | 12.77 |
|  |  | 2 | 13.83 |  |
| 0.25% | 3 | 1 | 8.05 | 6.85 |
|  |  | 2 | 5.65 |  |
| 0.25% | 4 | 1 | 13.82 | 15.89 |
|  |  | 2 | 17.95 |  |
| 0.25% | 5 | 1 | 12.40 | 10.68 |
|  |  | 2 | 8.95 |  |
| 0.5% | 1 | 1 | 27.77 | 39.94 |
|  |  | 2 | 49.19 |  |
| 0.5% | 2 | 1 | 11.39 | 16.31 |
|  |  | 2 | 19.90 |  |
| 0.5% | 3 | 1 | 9.34 | 16.48 |
|  |  | 2 | 17.11 |  |
| 0.5% | 4 | 1 | 15.05 | 17.97 |
|  |  | 2 | 10.18 |  |
| 0.5% | 5 | 1 | 10.44 | 8.24 |
|  |  | 2 | 5.65 |  |

**Table S6. Intra-assay and inter-assay %CVs of droplet removal strategies, over two wells with 9 replicants each well.**

| **Droplet removal** | **Well** | **Intra-assay CV [%]** | **Inter-assay CV [%]** |
| --- | --- | --- | --- |
| Kimwipe | 1 | 33.83 | 51.36 |
|  | 2 | 47.47 |  |
| Centrifuge | 1 | 19.52 | 42.08 |
|  | 2 | 41.62 |  |
| Pipette | 1 | 3.57 | 8.22 |
|  | 2 | 11.41 |  |

**Table S7. Intra-assay and inter-assay %CVs of relative humidity (%) throughout printing at various dry times, over two wells with 9 replicants each well.**

| **Humidity [%]** | **Dry time [min]** | **Well** | **Intra-assay CV [%]** | **Inter-assay CV [%]** |
| --- | --- | --- | --- | --- |
| 20 | 1 | 1 | 8.54 | 6.67 |
|  |  | 2 | 4.80 |  |
| 20 | 2 | 1 | 9.32 | 14.09 |
|  |  | 2 | 18.86 |  |
| 20 | 3 | 1 | 7.40 | 12.90 |
|  |  | 2 | 18.40 |  |
| 20 | 4 | 1 | 12.12 | 8.61 |
|  |  | 2 | 5.09 |  |
| 40 | 1 | 1 | 29.08 | 23.86 |
|  |  | 2 | 19.36 |  |
| 40 | 2 | 1 | 4.60 | 12.27 |
|  |  | 2 | 16.45 |  |
| 40 | 3 | 1 | 16.17 | 15.34 |
|  |  | 2 | 7.41 |  |
| 40 | 4 | 1 | 26.09 | 34.72 |
|  |  | 2 | 39.29 |  |
| 60 | 1 | 1 | 14.25 | 16.21 |
|  |  | 2 | 10.43 |  |
| 60 | 2 | 1 | 5.33 | 16.61 |
|  |  | 2 | 22.70 |  |
| 60 | 3 | 1 | 9.25 | 9.94 |
|  |  | 2 | 11.13 |  |
| 60 | 4 | 1 | 32.40 | 36.03 |
|  |  | 2 | 39.73 |  |
| 80 | 1 | 1 | 15.68 | 17.17 |
|  |  | 2 | 18.67 |  |
| 80 | 2 | 1 | 11.43 | 16.39 |
|  |  | 2 | 21.35 |  |
| 80 | 3 | 1 | 21.15 | 18.77 |
|  |  | 2 | 16.39 |  |
| 80 | 4 | 1 | 26.01 | 18.90 |
|  |  | **2** | 11.79 |  |

**Table S8. Intra-assay and inter-assay %CVs of force applied (kPa) while stamping, over two wells with 9 replicants each well.**

| **Force application [kPa]** | **Well** | **Intra-assay CV [%]** | **Inter-assay CV [%]** |
| --- | --- | --- | --- |
| 0 | 1 | 31.04 | 23.75 |
|  | 2 | 14.01 |  |
| 8.17 | 1 | 22.34 | 18.04 |
|  | 2 | 11.95 |  |
| 16.33 | 1 | 5.69 | 8.91 |
|  | 2 | 10.05 |  |
| 32.67 | 1 | 6.76 | 4.99 |
|  | 2 | 2.47 |  |
| 40.82 | 1 | 5.93 | 10.48 |
|  | 2 | 13.18 |  |
| 49 | 1 | 10.69 | 27.65 |
|  | 2 | 44.60 |  |

**Table S9. Intra-assay and inter-assay %CVs of manual, syringe pump, arbor press and combined automation strategies, over two wells with 9 replicants each well.**

| **Automation strategy** | **Well** | **Intra-assay CV [%]** | **Inter-assay CV [%]** |
| --- | --- | --- | --- |
| Manual | 1 | 9.79 | 7.73 |
|  | 2 | 5.66 |  |
| Syringe Pump | 1 | 28.96 | 22.18 |
|  | 2 | 8.85 |  |
| Arbor Press | 1 | 18.20 | 17.90 |
|  | 2 | 17.60 |  |
| Syringe Pump + Arbor Press | 1 | 7.08​ | 5.18 |
|  | 2 | 3.29​ |  |

**Table S10. Intra-assay and inter-assay %CVs of physical attachment compared to dual immobilization for nonlubricated and lubricant-infused microarrays, over two wells with 9 replicants each well.**

| **Automation strategy** | **Well** | **Intra-assay CV [%]** | **Inter-assay CV [%]** |
| --- | --- | --- | --- |
| Traditional | 1 | 22.15 | 66.12 |
|  | 2 | 26.65 |  |
| LIS Traditional | 1 | 20.15 | 14.60 |
|  | 2 | 5.56 |  |
| EDC/NHS | 1 | 9.69 | 8.41 |
|  | 2 | 6.19 |  |
| LIS EDC/NHS | 1 | 6.72​ | 15.46 |
|  | 2 | 17.59​ |  |
